# An analysis of telehealth in a post-pandemic rural, Midwestern community: increased comfort and a preference for primary care

**DOI:** 10.1186/s12913-025-12413-5

**Published:** 2025-02-18

**Authors:** Chase Salmon, Kameron Bell, Eric Reyes, Ellen Ireland, Robin Danek

**Affiliations:** 1 Indiana University School of Medicine, Terre Haute, IN USA; 2 Rose-Hulman Institute of Technology, Terre Haute, IN USA

**Keywords:** COVID-19 pandemic, Health services accessibility, Internet access, Rural health services, Telemedicine

## Abstract

**Background:**

The COVID-19 pandemic accelerated the adoption of telemedicine, integrating it into mainstream healthcare, especially in underserved and rural areas. This study examines the implementation and perceptions of telehealth in a rural Midwestern community in the post-pandemic period. Rural populations often face unique healthcare challenges. Telehealth has the potential to mitigate these issues by improving healthcare accessibility and patient satisfaction, thus being a vital topic for research.

**Methods:**

A survey was created and conducted from September to October 2023 to evaluate Internet access, telehealth usage, and perceptions among residents of a rural Midwestern county. The county received fiberoptic Internet in November 2021, offering a valuable glimpse at the impact of advanced internet. The survey, distributed both online and in person, garnered 253 valid responses. Statistical analyses, including chi-square tests, were performed using IBM SPSS to explore the relationships between survey responses.

**Results:**

Among the 253 participants, the majority were female (81.4%) with a median age of 50 years. Internet access varied, with 22% of paper survey respondents lacking home Internet compared to 1.3% of online respondents. Telehealth usage for participants increased from 5% pre-pandemic to 42.1% during the pandemic, with 21.8% continuing to use telehealth post-pandemic. Primary care visits were the most common telehealth appointments. Key concerns included a preference for in-person care and perceived lower quality of telehealth services. Despite these concerns, 59.7% of respondents were willing to use telehealth, rising to 67.5% if recommended by a healthcare provider. Comfort with telehealth was significantly linked to perceptions of Internet speed and stability.

**Conclusions:**

While broadband and fiberoptic Internet are associated with better telehealth experiences, other types of Internet also facilitated telehealth usage in our study, indicating that factors beyond access influence patient comfort and willingness to use telehealth. Our findings also reveal significant interest in telehealth for primary care, suggesting rural patients prefer familiar providers for telehealth interactions. Despite increased telehealth interest and usage during the pandemic, a decline post-pandemic indicates potential barriers exist, such as limited availability of healthcare providers. Exploring and addressing these barriers remains crucial for sustaining telehealth adoption and improving healthcare access in rural communities.

**Supplementary Information:**

The online version contains supplementary material available at 10.1186/s12913-025-12413-5.

## Background

Although telemedicine services have been around for years, the declaration of COVID-19 as a global pandemic by the World Health Organization in March 2020 [1] served as a major turning point in the use of telemedicine in healthcare. Telemedicine has many different definitions [2], but is defined by the United States government as primarily online, provider-based health care that utilizes electronic information and telecommunications technology to promote clinical care, public health, and more [3, 4]. These telemedicine services found increased usage during the COVID-19 pandemic, with one study identifying that telehealth increased from making up 0.3% of ambulatory patient contacts pre-pandemic to over 23% of these contacts during the pandemic [5]. Another study examining telemedicine for mental health services found a 45-fold increase in utilization of telemedicine for behavioral health services during the COVID-19 pandemic from their participants [6]. Due to this widespread increase in usage, it is important to understand how telemedicine and healthcare are utilized and impact different populations.

One such population suggested to benefit from an increase in telehealth is rural populations, which tends to be underserved in general and especially specialty care [7, 8]. For example, a study revealed that rural individuals had less access to mental health services than their urban counterparts and had a stronger interest in mental health telemedicine services [9]. Furthermore, rural populations have been shown to have worse mental health outcomes despite a similar prevalence of mental illness to urban populations [10]. The disparities go beyond mental health with the CDC reporting that rural Americans are more likely to die due to five of the highest causes of mortality than urban Americans [7]. Rural Americans also have higher rates of obesity [8], and as a result, increased risk for a multitude of health problems [11]. This is worsened by rural populations having decreased access to health care due to structural urbanism, which can be considered as an overall emphasis by healthcare systems and public health on large urban centers [12].

As previously mentioned, the rise in telemedicine represents a potential avenue for addressing these differences. One examination of telemedicine services in a rural community focusing on occupational, physical, and speech-language therapy reported overall positive feedback and perceptions from participants, which served as a measure of program effectiveness [13]. Other research focused on the importance and effectiveness of telemedicine for the treatment of substance use disorder, proposing it as an aspect of treatment, even post-pandemic [14]. This has extra implications in rural communities due to the increased rate of opioid mortality growth compared to urban communities [15]. Potential benefits must be weighed against potential negative effects that could arise from the digital divide, including the discrepancy in Internet access in some communities, whether due to Internet availability or access to Internet capable devices [16]. Specifically, rural communities have a lack of broadband access, which along with interstate licensing regulations and telehealth parity laws are barriers to telemedicine services [17].

Rural communities also face troubles with clinician comfort providing care via telemedicine and patient confidence and comfort with technological skills, although these troubles can be at least partially addressed with utilization of interprofessional care teams and family counselors [18]. In addition, the values and perceptions of rural individuals utilizing telehealth must be considered; one systematic review found rural individuals have four guiding values in terms of their views on telemedicine and willingness to engage with telemedicine services: familiar relationships, privacy and confidentiality concerns, acceptance of limited access to care, and resourcefulness and frugality [19]. Due to these concerns, it has been proposed telemedicine will feed into the Inverse Care Law that states disadvantaged populations needing greater access to healthcare receive less than more advantaged counterparts [20].

Some of these barriers have begun to be addressed. The creation of the interstate medical licensure compact has worked to simplify the process for clinicians to serve out-of-state patients via telemedicine [17]. Government funding initiatives served to promote the purchase of telemedicine equipment by providers and supported broadband infrastructure and Internet connectivity services in communities [21]. Reimbursement policies facilitating payment to providers and changes in health system policies to safeguard said providers further encouraged the implementation of telemedicine [21]. Voice-only telephone services offered a chance for patients without reliable broadband access to still access telemedicine, reducing accessibility barriers [22]. Evidence suggests, however, that videoconferencing is equal or better than voice-only services in terms of provider-related outcomes, diagnostic accuracy, and reducing healthcare utilization [23]. This implies that non-videoconferencing solutions to discrepancies in telemedicine and internet accessibility can actually worsen health disparities, highlighting the need to critically evaluate the progress of telemedicine throughout the COVID-19 pandemic and the impact of barriers and their potential solutions on patient populations.

Owing to the growing importance of telemedicine and the complexities of rural healthcare and health services, there is a need to monitor the progress of telemedicine in rural communities. Furthermore, data evaluating the state of telemedicine in rural communities post-pandemic are scarce. Even less research has examined the effects of telemedicine initiatives and barrier solutions. The purpose of this study was to examine the use of telemedicine and perceptions of telemedicine among residents in a small, rural Midwestern county following the COVID-19 pandemic.

## Methods

We conducted a survey to evaluate Internet access, telehealth usage, and opinions on telehealth in a rural community. The survey was created specifically for this study and is included in the supplementary files. The community under study was unique in that fiberoptic Internet was introduced to the county in November 2021, roughly midway through the Covid-19 public health emergency. The surveys were made available online and in-person from September to October 2023. In-person surveys were made available at public libraries and offered at a local festival. Online surveys were distributed through social media. In addition, a flyer was included in a local newspaper with a QR code to recruit participants.

### Inclusion criteria

To be eligible for participation, respondents had to reside in the county being studied and be aged 18 years or older. Of the 253 valid participants, 18 completed a paper survey and 235 completed an online survey. Of those completing the paper survey, 22% did not have Internet access at home, compared to only 1.3% of online survey respondents who did not have Internet access at home. Demographic data are shown in (Table 1).

**Table 1:** Participant demographic data

**Gender**	**n (percent)**	**Population Data**
Male	47 (18.6%)	52%
Female	206 (81.4%)	48%
Total N	253	
**Age**		
18 to 24	22 (8.8%)	740 (7.5%)
25 to 34	37 (14.7%)	1058 (10.7%)
35 to 44	42 (16.7%)	1126 (11.4%)
45 to 54	51 (20.3%)	1221 (12.4%)
55 to 64	48 (19.1%)	1483 (15.0%)
65 to 84	49 (19.5%)	1817 (18.4%)
85 +	2 (0.8%)	166 (1.7%)
Total N	251	
**Household Income**		
Less than $30,000	30 (12.0%)	
$30,000—$59,999	62 (24.7%)	
$60,000—$89,999	59 (23.5%)	
$90,000—$119,999	51 (20.3%)	
Greater than $120,000	49 (19.5%)	
Total N	251	

Table revealing demographic data for survey participants. Participants were allowed to skip questions and drop out as desired. Thus, percentages are reported in relation to number of participants who answered each question. Population data obtained from U.S. Census Bureau [24] and STATS Indiana [25]

### Statistical analysis

Quantitative variables were summarized using quartiles and categorical variables were summarized using frequencies and relative frequencies. Chi-square tests of independence were conducted to examine the potential relationships between the responses to questions in the survey. Statistical analysis was performed with IBM SPSS.

### Ethics approval

This study was approved by the Institutional Review Board of Indiana University under protocol number 20288. Informed consent was obtained from participants through the means of the survey introduction, stating: “Participation in this survey is completely voluntary. Participants are free to withdraw at any time throughout the survey. No compensation will be provided for participation in this study. All information will be kept confidential and anonymous, and no personal identifiable information will be collected.” This served to inform participants of the study and any risks/benefits associated with participation. Continuation of the study represented consent.

## Results

Two hundred seventy individuals participated in the survey. Eight participants were excluded because they were not residents of the county under study, and another nine participants opened the online survey but quit before answering a question, resulting in 253 participants. The majority of the participants were female (81.4%), with a median age of 50 years. Demographic data are shown in (Table 1). Due to the voluntary nature of the study, the participants were allowed to drop out at any time and/or skip questions. Therefore, the reported percentages were based on the number of respondents to each question.

When asked where they were completing the survey, 51 (*N* = 253, 20.2%) were online in a public setting such as a library, 17 (6.7%) were in public using a paper survey at a local festival, 184 (72.7%) were online from home, and one (0.4%) completed the survey on paper at home. Only seven respondents (*N* = 231, 3.0%) stated that they did not have Internet access at home, and 142 (*N* = 224, 63.4%) reported having either fiberoptic or broadband Internet access. Of note, 92 (*N* = 224, 41.1%) individuals had not upgraded their Internet in over two years, with a majority of these participants having broadband or fiberoptic connections.

Of those surveyed only 12 (*N* = 242, 5%) individuals utilized telehealth before March 2020, but this number increased to 98 (*N* = 233, 42.1%) during the Covid-19 pandemic. Since the end of the federal Covid-19 public health emergency in May 2023, 53 (*N* = 238, 21.8%) respondents reported having utilized telehealth during this time. Following the introduction of fiberoptic Internet to the county of study in November 2021, two-thirds of the participants said their usage of telehealth was roughly the same. For all three time periods, the most common type of telehealth appointment was a primary care visit with 41.7% (pre-Covid), 47.6% (during Covid), and 34.0% (post-Covid) of respondents that had a telehealth appointment during those respective periods having had a primary care visit. This was reflected in the responses to the question regarding the types of services that respondents would consider for telehealth appointments (Table 2).

**Table 2:** This multiple response question set yielded a total of 504 responses from the 203 question participants

Telehealth Services Participants would Consider as Patients	*Would Not Consider* *n* =	*Would Consider* *n* =	*Percentage of Respondents Considering Service*
Behavioral/Mental Health Therapy	118	85	42%
Physical Therapy	170	33	16%
Surgery Follow-up	138	65	32%
Primary Care Visit	76	127	63%
Substance Abuse Treatment Program	189	14	7%
Specialty Care	168	35	17%
Medication Adjustment Appointment	70	133	66%
None	196	7	3%
Other	198	5	2%
Total Number of Respondents (N)	203		

The three most common concerns regarding telehealth (*N* = 231) were preference for in-person care (124, 53.7%), belief that telehealth had a lower quality of care (80, 34.6%), and lack of quality Internet service (42, 18.2%). In contrast, 51 (22.1%) participants reported no concerns regarding telehealth. The three most common perceived benefits of telehealth (*N* = 230) were reduced exposure to sick individuals (*n* = 145, 63.0%), avoidance/minimization of travel (*n* = 144, 62.6%), and improved access to primary care (*n* = 79, 34.3%).

Overall, a majority (*N* = 231, *n* = 138, 59.7%) of the participants were willing to consider a telehealth appointment. An additional 28.1% (*n* = 65) responded “maybe”. However, if suggested by a healthcare provider, the number of participants who would utilize telehealth increased to 67.5% (*N* = 231, *n* = 156).

**Fig. 1:**
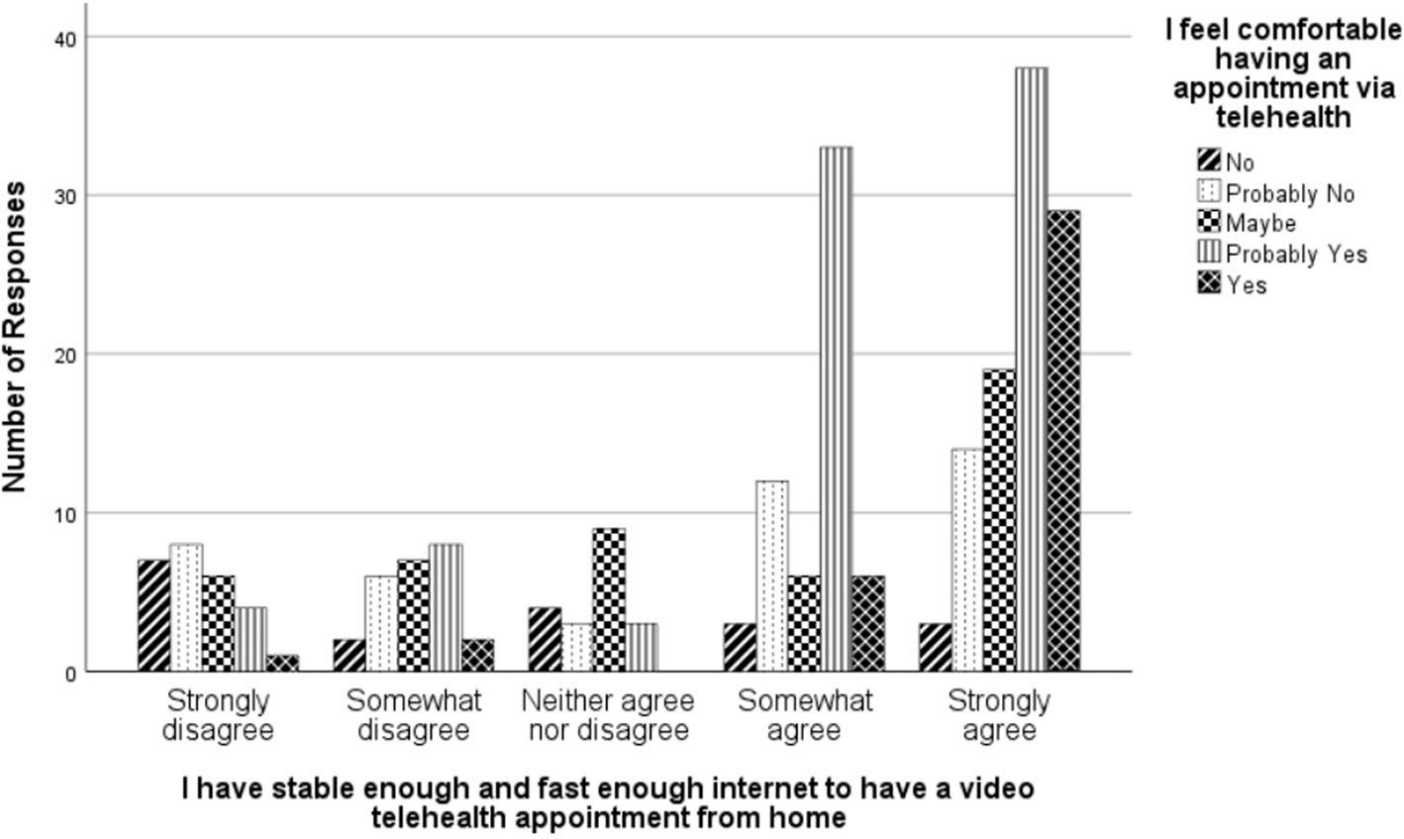
Telehealth comfort in relation to internet stability and speed. Bar chart comparing comfort with having a telehealth appointment to feelings of stable enough Internet and fast enough Internet to have a video telehealth appointment. The chart indicates higher feelings of both fast and stable Internet connections are associated with higher levels of comfort having a telehealth appointment

Our analysis revealed a significant relationship between feelings of Internet stability/speed and comfort with having a telehealth appointment (*N* = 233, *p* < 0.001). As shown in (Fig. 1), those with increased stability/speed were more likely to be comfortable with a telehealth appointment.

We also found evidence that participants’ feelings about their Internet speed and stability were related to participants’ consideration of telehealth if suggested by a provider (*N* = 233, *p* < 0.001); specifically, those with increased Internet speed and stability were more likely to consider telehealth when suggested by a healthcare provider.

**Fig. 2:**
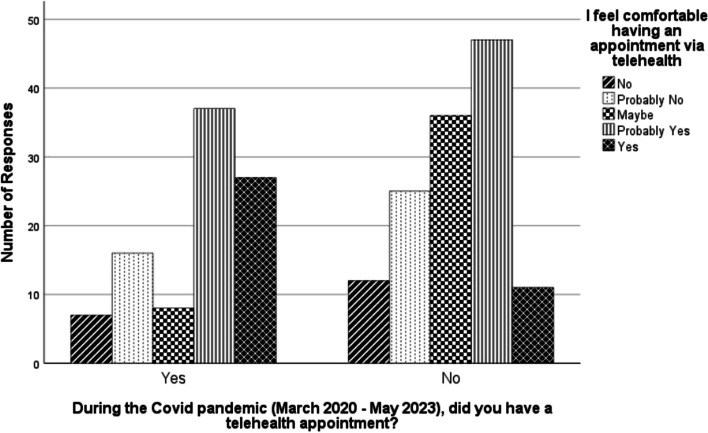
Telehealth comfort in relation to having a prior telehealth appointment. Bar chart representing the relationship between having had a telehealth appointment during the Covid-19 pandemic and feeling comfortable having a telehealth appointment. The chart suggests having a previous telehealth appointment during the pandemic is associated with higher levels of comfort having a telehealth appointment

We found evidence of a relationship between participants’ consideration of a telemedicine appointment and whether they had a telehealth appointment during the COVID-19 pandemic (*N* = 226, *p* < 0.001). Of the 95 respondents who said they had a telehealth appointment during the pandemic, 74 reported that they would consider having a telehealth appointment and 13 stated that they might consider a telemedicine appointment. Similarly, having a telehealth appointment during the pandemic was associated with a higher likelihood of feeling comfortable having a telehealth appointment (*N* = 226, *p* < 0.001). This is illustrated in(Fig. 2).

## Discussion

This study found that participants’ comfort with telehealth appointments was significantly associated with their perceptions of speed and stability of their Internet service. Additionally, willingness to use telemedicine when recommended by a provider was similarly linked to participants’ confidence in having fast and stable Internet. Notably, while 60% of respondents reported having broadband or fiberoptic Internet, 70% believed their Internet was fast and stable enough for telehealth. This discrepancy suggests that rural residents perceive alternative Internet options as sufficient for telehealth, highlighting that broadband and fiberoptic connections, while often considered superior, are not the only adequate Internet avenues for telemedicine. This highlights the notion that factors other than broadband access influence rural telemedicine usage. This takeaway is further supported by previous research exploring Rural Nursing Theory and rural health-seeking profiles [19]. Despite these nuances, we do believe efforts to improve rural Internet access are still endeavors worth pursuit and will only serve to bolster rural telemedicine.

A key finding of this study is the widespread interest in telehealth among participants, with an even larger proportion indicating they would use telemedicine if recommended by their provider. However, only 42.1% (*n* = 98) reported using telehealth during the pandemic, and just 21.8% (*n* = 53) had a telemedicine appointment since May 2023. This highlights a persistent disconnect between interest in and utilization of telemedicine, a pattern consistent with pre-pandemic findings indicating a larger interest than true usage [26]. Historically, this discrepancy has been attributed primarily to limited broadband access [16, 17, 20], though our findings suggest this explanation may be incomplete. In addition, high satisfaction rates among rural telehealth users, as found in prior studies, indicate that dissatisfaction with telemedicine services is unlikely to explain this gap [13].

One potential contributor to this disconnect is the shortage of healthcare providers in rural areas, including primary care providers [12]. Participants expressed a strong interest in telemedicine for primary care visits. Rural primary care providers may be preoccupied with in-person care demands, and thus less inclined or able to offer telehealth appointments. Especially in the post-pandemic period, providers may simply be offering less telemedicine options. This underscores the need for further research into the role of providers in bridging the gap between telehealth interest and usage, particularly in the evolving post-COVID-19 landscape.

Contrary to our hypothesis, participants expressed greater interest in telemedicine for primary care visits rather than specialty care. While specialty care has historically been a primary focus of telehealth in rural areas [19], it ranked fifth out of seven options for telemedicine use in our survey. Participants’ lower interest in telehealth for specialty care, considered with the findings regarding concerns about the quality of telemedicine and a preference for in-person care, suggests that rural residents may prefer to travel for specialty care to receive a perceived higher quality of healthcare and to build trusted patient-provider relationships. Previous research has highlighted the importance of trust and familiarity in rural healthcare interactions, as well as rural patient beliefs that these traits are harder to build via telehealth [19]. This warrants further investigation to better understand rural patients’ preferences and decision-making processes regarding telehealth for specialty care.

Participants’ interest in telemedicine for primary care visits may stem from a desire to use telehealth for routine or minor concerns. Responses to the open-ended question, “What is your overall opinion on telehealth in Martin County?” reflect this sentiment, with participants suggesting telehealth’s utility for managing “minor” illnesses like “colds or flu” and for straightforward needs, such as “antibiotic prescriptions for sinusitis”. These findings align with previous research mentioned earlier indicating that rural patients value relationships with trusted primary care providers and may prefer telemedicine interactions with familiar providers [19].

An interesting trend noted in this study is the increase in telehealth usage among participants, from 5% (*n* = 12) prior to the COVID-19 pandemic to 42.1% (*n* = 98) during the pandemic, supporting previous findings [5, 6]. While usage has decreased to 21.8% (*n* = 53) in the post-pandemic period, this remains higher than pre-pandemic levels. Thus, this suggests a sustained shift in telehealth adoption. This trend may reflect satisfaction with telemedicine during the pandemic, as supported by the found association between comfort with telehealth and prior telemedicine usage. While not explored in this study, the drop in telemedicine usage from the pandemic to post-pandemic periods may indicate a general decline in healthcare visits or a waning interest in telehealth. If telemedicine is to serve as an avenue for improved rural healthcare, the increased usage will need to persist. Therefore, further monitoring is needed to assess the sustainability of these changes.

The findings of this study can also be looked at more broadly to explore telemedicine in other contexts. Disaster-stricken areas, much like rural regions, often face provider shortages and limited resources, compounded by increased patient volume [7, 12, 27]. Primary receiving hospitals may also be forced to provide critical care for which they are not adequately prepared [27]. Challenges such as bandwidth constraints in disaster settings align with this study’s finding that Internet options beyond broadband can support telehealth, supporting prior work recommending low-bandwidth telemedicine solutions to address equity concerns in resource-limited areas [27]. In addition, this can also help address ethical concerns regarding equitable availability of telehealth in disaster areas [28].

Furthermore, this study’s results contribute to understanding similarities and differences in telemedicine preferences and perceptions between rural and urban populations. In agreement with previous studies, this study found that prior usage of telemedicine was related to an increased interest in future virtual visits, which has been noted in both rural and urban populations [29]. Prior research indicates that rural patients are more likely to use telemedicine for primary care than for mental health care, a trend observed in this study but not observed in urban populations [30]. In addition, a different study found that a rural RUCC decreased the likelihood of a patient having a psychiatric telemedicine appointment compared to urban counterparts [31]. These distinctions underscore the importance of understanding the differences between rural and urban populations regarding telehealth, and similarly, the importance to tailoring telemedicine in rural communities to meet their unique needs and desires.

Future research should focus on understanding the disconnect between telehealth interest and usage. Evidence continues to suggest that factors other than internet and technology accessibility play a pivotal role in the observed disconnect, yet work exploring other factors is lacking. Furthermore, rural interests in telemedicine need to be explored, particularly regarding primary versus specialty care. Investigating rural patients’ preferences for telehealth in non-primary care contexts and exploring the factors contributing to the decline in telehealth usage post-pandemic could provide valuable insights. Additionally, studies should address the implications of ongoing findings for reducing rural health disparities and enhancing telemedicine’s ability to bolster efforts in achieving equitable healthcare access.

This study has limitations. Inconsistent participation, with respondents skipping questions or dropping out of the survey, may have introduced bias. Additionally, the disproportionate number of online responses compared to paper surveys is notable in a study focused on telehealth. Future research should aim to balance data collection methods to ensure a more representative sample. A notable strength of this study is its focus on a community that transitioned to fiberoptic Internet midway through the COVID-19 pandemic, offering unique insights into the effects of improved Internet access on telehealth adoption.

## Conclusions

Since the start of the Covid-19 pandemic, telehealth has seen a rise in usage and study. One area of increased focus is telehealth in rural communities. There are beliefs that telemedicine could reduce discrepancies between rural and urban communities and serve to increase healthcare access to the rural population. Others caution that telemedicine could worsen the gap between rural and urban areas. As we progress from the Covid-19 pandemic, it is important to monitor telemedicine in rural communities and examine its impact on rural communities.

This study found that while telehealth usage has remained elevated post-pandemic compared to its prior usage, there remains a large discrepancy between interest in telehealth and its usage. Unexpectedly, rural individuals were more interested in primary care appointments for telehealth than specialty care appointments. In addition, while much focus on barriers to telehealth has been centered on Internet access, our study found that a majority of participants felt that their Internet was stable enough and fast enough for video telehealth. Furthermore, even as better Internet options such as fiberoptic become available in the county of study, we found that many people are not upgrading their service and the introduction of these services does not impact telehealth usage. Therefore, it would be beneficial for future work to explore barriers other than Internet access and delve further into the discrepancy between telehealth interest and usage.

## Supplementary Information

Supplementary Material 1.

Supplementary Material 2.

## Data Availability

All collected survey data is available through contacting the corresponding author, Chase Salmon, and is included in the supplementary information files.
