# Supplementary material for: An analysis of telehealth in a post-pandemic rural, Midwestern community: increased comfort and a preference for primary care
Source: BMC Health Serv Res. 2025 Feb 18;25:270. doi: 10.1186/s12913-025-12413-5 (PMC11837642; doi:10.1186/s12913-025-12413-5)
Supplement: Supplementary file 1 — Supplementary Material 1. [file 12913_2025_12413_MOESM1_ESM.pdf]

INDIANA UNIVERSITY

Survey Introduction

Hello!

Thank you for taking the time to participate in this survey. This project is being completed as a requirement for a student-led public health research project at the Indiana University School of Medicine. This survey will contain questions regarding telehealth usage, views on telehealth, and internet access. Questions on this survey will focus on the time period during the COVID pandemic as well as the post-pandemic timeframe. Lastly, some questions utilize November 2021 as a timestamp as this marks the beginning of fiber optic internet

availability in Martin County.

To be eligible for this study, participants must currently reside in Martin County, IN, and you must be 18 or older.

Participation in this survey is completely voluntary. Participants are free to withdraw at any time throughout the survey. No compensation will be provided for participation in this study.

All information will be kept confidential and anonymous, and no personal identifiable information will be collected. Please answer questions honestly and to the best of your ability. Any questions regarding the survey can be directed to Chase Salmon via email at chmsalmo@iu.edu

Thank you!
Chase M Salmon

Demographics

The first section focuses on collecting information about who you are (i.e. age, gender, etc.) Please select your best answer for each question.

Do you live in Martin County, IN?

- ○ Yes
- ○ No

What is your age? (Participants must be over the age of 18)

[ ]

Which of the following best describes your gender?

- Male
- Female
- Non-binary / third gender
- Prefer not to say

What is your household's annual income?

- less than $30,000
- $30,000 - $59,999
- $60,000 - $89,999
- $90,000 - $119,999
- greater than $120,000

How are you completing this survey?

- Public setting on your phone/computer etc.
- Public setting on paper

- At home on your phone/computer etc.
- At home on paper

Internet Access

This section will focus on your internet access. Please select your best answer for each question.

Do you have internet access in your household (wifi, mobile plan/cell phone data, etc.)

- Yes
- No

How would you describe your internet usage?

- Multiple times daily
- Once a day
- Multiple times a week
- Once a week
- Multiple times a month
- Once a month
- Less than monthly

Who is your internet service provider?

- Cell phone company
- Hughes Net
- Viasat
- Space Exploration Technologies
- T-Mobile
- Frontier Communications
- Cable One
- RTC
- ALTIUS
- Greenwell

- Smithville
- Unsure
- Other (please specify)

What type of internet service do you have?

- Fiber Optic
- Broadband (DSL or Cable)
- Satellite
- Cell phone data only
- Cellular modem
- Unsure
- Other (please specify)

When did you last upgrade your internet service (change providers or improve speed)?

- Within the last month
- Within the last 6 months
- Within the last year
- Within the last two years
- Over two years ago

Telehealth Usage

This section will collect information regarding your usage of telehealth. Telehealth includes video appointments from home on your own device, appointments using video services from your doctor's office to see another provider, and appointments that occurred over the phone.

The Covid pandemic is considered to be from March 2020 - May 2023.

Please answer honestly and to the best of your ability.

Before March 2020 (the start of the COVID pandemic), did you have a telehealth appointment?

- Yes
- No

How many telehealth appointments did you have before March 2020?

- 1-5
- 6-10
- 11-15
- 16-20
- 21+

What type of telehealth services did you utilize before March 2020?
Select all that apply

- ☐ Video appointment from home
- ☐ Video from physician's office to another provider
- ☐ Telephone appointment

What appointments did you have via telehealth before March 2020?
Select all that apply

- ☐ Behavioral/Mental health therapy
- ☐ Physical therapy (guided exercises, post-operation therapy, etc.)
- ☐ Surgery follow-up
- ☐ Primary Care visit (family medicine, internal medicine, etc.)
- ☐ Substance abuse treatment program
- ☐ Specialty care (cardiologist, pulmonologist, nephrologist, etc)
- ☐ Appointment to discuss and adjust medications only

☐ Other (please specify) ____________

During the Covid pandemic (March 2020 - May 2023), did you have a telehealth appointment?

○ Yes

○ No

From March 2020 - May 2023, how many telehealth appointments did you have?

○ 1-5

○ 6-10

○ 11-15

○ 16-20

○ 21+

What type of telehealth services did you utilize from March 2020 - May 2023?
Select all that apply

- ☐ Video appointment from home
- ☐ Video from physician's office to another provider
- ☐ Telephone appointment

What appointments did you have via telehealth from March 2020 - May 2023
Select all that apply

- ☐ Behavioral/Mental health therapy
- ☐ Physical therapy (guided exercises, post-operation therapy, etc.)
- ☐ Surgery follow-up
- ☐ Primary Care visit (family medicine, internal medicine, etc.)
- ☐ Substance abuse treatment program
- ☐ Specialty care (cardiologist, pulmonologist, nephrologist, etc)
- ☐ Appointment to discuss and adjust medications only

☐ [ ] Other (please specify)

Since the introduction of fiber optic internet to Martin County in November 2021, how did your telehealth usage change?

- ○ Utilized telehealth more before November 2021
- ○ Utilized telehealth roughly the same before and after November 2021
- ○ Utilized telehealth more since November 2021

Since May 2023 (post-pandemic), have you had a telehealth appointment?

- ○ Yes
- ○ No

Since May 2023, how many telehealth appointments have you

had?

- ○ 1-5
- ○ 6-10
- ○ 11-15
- ○ 16-20
- ○ 21+

Since May 2023, what type of telehealth services have you utilized?
Select all that apply

- ☐ Video appointment from home
- ☐ Video from physician's office to another provider
- ☐ Telephone appointment

Since May 2023, what appointments have you had via telehealth?

Select all that apply

- ☐ Behavioral/Mental health therapy
- ☐ Physical therapy (guided exercises, post-operation therapy, etc.)
- ☐ Surgery follow-up
- ☐ Primary Care visit (family medicine, internal medicine, etc.)
- ☐ Substance abuse treatment program
- ☐ Specialty care (cardiologist, pulmonologist, nephrologist, etc)
- ☐ Appointment to discuss and adjust medications only
- ☐ [ ] Other (please specify)

Telehealth Opinions

This is the last section of the survey. It will focus on your opinions of telehealth. Some questions will ask you how much you agree with given statements. Please answer honestly.

I feel comfortable having an appointment via telehealth

- Strongly disagree
- Somewhat disagree
- Neither agree nor disagree
- Somewhat agree
- Strongly agree

I have stable enough and fast enough internet to have a video telehealth appointment from home

- Strongly disagree
- Somewhat disagree
- Neither agree nor disagree
- Somewhat agree
- Strongly agree

If suggested by my healthcare provider, I would use telehealth for an appointment

- Strongly disagree
- Somewhat disagree
- Neither agree nor disagree
- Somewhat agree
- Strongly agree

Would you ever consider having a telehealth appointment?

- No
- Maybe
- Yes

What services would you consider using telehealth for? Select all that apply

- Behavioral/Mental health therapy

- ☐ Physical therapy (guided exercises, post-operation therapy, etc.)
- ☐ Surgery follow-up
- ☐ Primary Care visit (family medicine, internal medicine, etc.)
- ☐ Substance abuse treatment program
- ☐ Specialty care (cardiologist, pulmonologist, nephrologist, etc)
- ☐ Appointment to discuss and adjust medications only
- ☐ None
- ☐ ______ Other (please specify)

What concerns do you have regarding the use of telehealth? Select all that apply

- ☐ I do not have quality internet service
- ☐ I do not have internet capable devices (smartphone, iPad, laptop/computer, etc.)
- ☐ I prefer in-person care
- ☐ I think telehealth would have a lower quality of care
- ☐ I lack knowledge/information on telehealth programs
- ☐ I have a lack of privacy at home

- ☐ None
- ☐ [ ] Other (please specify)

What potential benefits do you associate with the use of telehealth?
Select all that apply

- ☐ Improved access to primary care
- ☐ Improved access to specialty care
- ☐ I can avoid people I know seeing me in the waiting room
- ☐ I can avoid/minimize travel
- ☐ There is reduced exposure to sick individuals
- ☐ I can access different providers
- ☐ None
- ☐ [ ] Other (please specify)

OPTIONAL
What is your overall opinion on utilizing telehealth in Martin County? You can list concerns, benefits, complaints, or personal experiences.

Powered by Qualtrics
