# Supplementary material for: An analysis of telehealth in a post-pandemic rural, Midwestern community: increased comfort and a preference for primary care
Source: BMC Health Serv Res. 2025 Feb 18;25:270. doi: 10.1186/s12913-025-12413-5 (PMC11837642; doi:10.1186/s12913-025-12413-5)
Supplement: Supplementary file 2 — Supplementary Material 2. [file 12913_2025_12413_MOESM2_ESM.zip › Telehealth Survey Responses.pdf]

StartDate	EndDate	Status	IPAddress	Progress	Duration (ii	Finished	RecordedD	ResponseI(	RecipientLa	RecipientFi	RecipientE	ExternalRe
Start Date	End Date	Response T	IP Address	Progress	Duration (ii	Finished	Recorded D	Response I	Recipient L	Recipient F	Recipient E	External Da
########	########	IP Address	159.218.8 2.205	100	210	True	########	R_qIaqK1 VEPejBglz				

########	########	IP Address	216.176.1 00.185	100	179	True	########	R_1rewGw jbZfsgFqa

########	########	IP Address	47.243.18<br>3.142	100	93	True	########	R_3exepK<br>u02qc7AU<br>q

########	########	IP Address	173.47.43.215	100	241	True	########	R_1pVFy3UbqYiugMD

########	########	IP Address	67.61.140<br>.126	100	623	True	########	R_1QMVn<br>VG0Tuoie<br>7n

########	########	IP Address	66.244.11<br>3.72	100	284	True	########	R_2zSF8ic<br>UdGEuGgj

########	########	IP Address	172.56.249.222	100	301	True	########	R_2QL55aM316pR3Dw

########	########	IP Address	174.202.103.85	100	225	True	########	R_3rVtNBuRaj2943P

########	########	IP Address	174.202.102.202	100	423	True	########	R_3nloj3fGVQJd4ui

########	########	IP Address	184.16.11 8.66	100	208	True	########	R_PYxvRiE Ns2Sc8lb

########	########	IP Address	50.104.27.83	100	196	True	########	R_1NDldoTHUa5JCYJ

########	########	IP Address	50.104.28.58	100	447	True	########	R_3HIbLKw483tyANQ

########	########	IP Address	50.104.16<br>7.70	100	635	True	########	R_2AEHp<br>Dlvbg9m9<br>jr

########	########	IP Address	173.47.56.251	100	413	True	########	R_1OoY5D7fQ8wmHjd

########	########	IP Address	67.45.32.58	100	1482	True	########	R_1QryOcNAtkIUMER

########	########	IP Address	50.104.31.213	100	325	True	########	R_1kTGuEHMPHtj1Nc
########	########	IP Address	50.127.30.236	100	341	True	########	R_3yHbUaNuQJ3nS7v

########	########	IP Address	174.202.101.132	100	312	True	########	R_pmjfEn30egSabnP
########	########	IP Address	216.176.123.194	100	1491	True	########	R_3HZatxr3NxKITZm

########	########	IP Address	166.205.1<br>41.13	100	467	True	########	R_2Qm5Z<br>P7Gy79RJ<br>nQ

########	########	IP Address	50.127.53.2	100	217	True	########	R_2t9UGVBUfKlV2Jj

########	########	IP Address	216.176.1 22.240	100	131	True	########	R_2ar9Ku W4x8DwG gJ
########	########	IP Address	216.176.1 00.211	100	213	True	########	R_xtKOUJ anaDK6zF T

########	########	IP Address	192.174.9 9.130	100	1421	True	########	R_1oBmb vok2fa44 MO

########	########	IP Address	168.75.198.182	100	301	True	########	R_DP5SDgoIOLZNqpz

########	########	IP Address	50.104.28.58	100	406	True	########	R_3Dqp2Eck6mWmPGM

########	########	IP Address	174.202.1 05.163	100	359	True	########	R_3iJailok hBvotjj
########	########	IP Address	168.75.20 4.145	100	11	True	########	R_3PmyZ QRDhGQ6 bMB

########	########	IP Address	216.176.1 19.218	100	194	True	########	R_3RpVEC PAW6Ogw Xy

########	########	IP Address	192.174.1<br>05.75	100	287	True	########	R_OGVpy<br>nkUgna9a<br>7L
########	########	IP Address	168.75.19<br>8.52	100	144	True	########	R_2WUAn<br>Tb2M32ze<br>0p

########	########	IP Address	174.202.97.44	100	52	True	########	R_87Ha5QWDFdKqYql
########	########	IP Address	216.176.110.87	100	220	True	########	R_3oNsLXGvqgFvyMo

########	########	IP Address	98.97.11.83	100	113	True	########	R_55a8sAXIb25nddf

########	########	IP Address	216.176.122.161	100	234	True	########	R_O3Ev1YjvUrQYW0p
########	########	IP Address	216.176.100.214	100	229	True	########	R_shDXBFJIEVibbMd
########	########	IP Address	173.47.57.218	100	164	True	########	R_OBSsc7u4VyP5Snf

########	########	IP Address	174.202.1 04.133	100	194	True	########	R_1GvOD pN0a10ZP N9

########	########	IP Address	174.202.9<br>8.241	100	586	True	########	R_22LE83<br>ZhBOcSB<br>hX

########	########	IP Address	174.202.105.15	100	2319	True	########	R_ufX8UivTxbmYe9X
########	########	IP Address	173.47.58.100	100	181	True	########	R_2S2ZWJ5mSfahS5r

########	########	IP Address	172.56.248.14	100	234	True	########	R_3dNgwpRq7XmTc4I

########	########	IP Address	192.174.1<br>26.42	100	318	True	########	R_eKISfQK<br>YdPjQwDv

########	########	IP Address	98.97.12.248	100	304	True	########	R_2Sxg9s6HgLaCC4E

########	########	IP Address	192.174.98.143	100	699	True	########	R_5BVu0BuKJnUbnLX

########	########	IP Address	216.176.124.143	100	246	True	########	R_2zSGo6SYETOCRH6

########	########	IP Address	216.176.124.84	100	1384	True	########	R_2taHFxGwZOpBCp4

########	########	IP Address	172.78.23<br>7.172	100	154	True	########	R_3MKFYp<br>PoatNAcM<br>U
########	########	IP Address	24.142.76<br>.238	100	369	True	########	R_3g74lqy<br>4ALFKmM<br>R

########	########	IP Address	216.176.100.5	100	686	True	########	R_2rYCu3xUGW9BqF

########	########	IP Address	192.174.101.30	100	282	True	########	R_3nqMkZB1xYmrwkr

########	########	IP Address	216.176.125.42	100	334	True	########	R_1mJIKgy17QPDlTM
########	########	IP Address	75.211.219.89	100	284	True	########	R_3EEC94hbcOePd77

########	########	IP Address	192.174.9<br>8.44	100	280	True	########	R_1OBjz3<br>XaizJT2K1

########	########	IP Address	216.176.101.145	100	232	True	########	R_3242gh1mc76PhqL

########	########	IP Address	168.75.19 8.80	100	182	True	########	R_eSb7b3 7FS9tUHg 5
########	########	IP Address	174.202.9 9.183	100	228	True	########	R_1IQlAgq Zi30gGfI

########	########	IP Address	184.16.18 5.164	100	1925	True	########	R_2YFenS wuDApX5I s

########	########	IP Address	174.202.99.156	100	416	True	########	R_3EglMKOtX2qmE0D

########	########	IP Address	216.176.101.21	100	332	True	########	R_3JxcwwlAYZStSVN

########	########	IP Address	76.181.4.48	100	222	True	########	R_2xRXGUCj7qcK6KH

########	########	IP Address	50.102.196.123	100	201	True	########	R_9YzlQjBwzs7w5Mt

########	########	IP Address	168.75.19 8.133	100	562	True	########	R_1kZZoH lynPoiVpq

########	########	IP Address	216.176.100.29	100	459	True	########	R_1f3iM6v0pub1xbM

########	########	IP Address	216.176.118.159	100	157	True	########	R_2tahdxq3KcxYI7h

########	########	IP Address	216.176.119.74	100	470	True	########	R_1GIdQ3go5ByXq9N
########	########	IP Address	192.174.98.47	100	190	True	########	R_3I9PSu2PNW3hecJ

########	########	IP Address	192.174.97.56	100	389	True	########	R_Pu3qRecy15wjskN

########	########	IP Address	216.176.101.145	100	504	True	########	R_3qByQ8lWNAunbVf

########	########	IP Address	168.75.19<br>8.198	100	299	True	########	R_OPSAzS<br>tKOiK9wh<br>b

########	########	IP Address	216.176.1 01.152	100	191	True	########	R_2SxzuY S3flcNfJN
########	########	IP Address	50.104.1. 193	100	164	True	########	R_2SGHy GoGC8z4 TCf

########	########	IP Address	216.176.122.155	100	245	True	########	R_6D0mRmgoWm26RRD
########	########	IP Address	107.127.49.23	100	238	True	########	R_3OA2iHqUvOGeMgN

########	########	IP Address	184.16.18<br>6.204	100	584	True	########	R_3NKRql<br>ZvDXpU5<br>UF
########	########	IP Address	174.202.9<br>8.81	100	185	True	########	R_1C30R6<br>egbu4OAh<br>v
########	########	IP Address	168.75.19<br>8.66	100	138	True	########	R_1N8Gc<br>4E59Vyfi3<br>P

########	########	IP Address	216.176.1 22.6	100	269	True	########	R_3nPl1X pTFU0sDJ Y
########	########	IP Address	172.59.81 .205	100	31	True	########	R_27etqE KC4OkifY o

########	########	IP Address	216.176.1 01.134	100	318	True	########	R_2XiIfOV 58HBmhY z
########	########	IP Address	192.174.9 9.130	100	241	True	########	R_UL6Mly dJPu2oS3f

########	########	IP Address	168.75.19 8.169	100	222	True	########	R_40yL4z QbXzBm2 Fb
########	########	IP Address	166.199.1 52.45	100	207	True	########	R_21vtbOf 9aXH9bSJ
########	########	IP Address	192.174.1 01.188	100	273	True	########	R_RPukZk YLzLb2C7 T

########	########	IP Address	174.202.106.220	100	452	True	########	R_pN0xU9FK6082AVP

########	########	IP Address	174.202.9 8.124	100	314	True	########	R_RQqBY bdiCM4K wvL

########	########	IP Address	168.75.198.197	100	811	True	########	R_saFYjpwwqnJNDJn

########	########	IP Address	173.47.58.30	100	181	True	########	R_1JRciA7ywboTWlf
########	########	IP Address	75.245.200.189	100	387	True	########	R_2CHqjxOIaYhRNwT

########	########	IP Address	76.11.172.154	100	371	True	########	R_2Tueold5RpduYzM
########	########	IP Address	216.176.123.239	100	205	True	########	R_1PXLrRbhQMzGpMO

########	########	IP Address	216.176.101.5	100	310	True	########	R_1kHe7XwNx8w6Kmj

########	########	IP Address	173.47.57.131	100	422	True	########	R_pcGU429icqpsPOF
########	########	IP Address	216.176.100.94	100	130	True	########	R_1CyIOgRvEVNyYYh

########	########	IP Address	168.75.19 8.92	100	913	True	########	R_31LdoD imvxt7jFl

########	########	IP Address	24.142.76.43	100	463	True	########	R_2P7DbNDx1kLvsnH

########	########	IP Address	75.107.57<br>.190	100	329	True	########	R_AKwRLT<br>eulTdO6f<br>D
########	########	IP Address	174.239.1<br>16.233	100	291	True	########	R_TcHZo3<br>pny0LQ6c<br>N

########	########	IP Address	174.202.104.86	100	345	True	########	R_24uuALSju8FR8M7

########	########	IP Address	168.75.198.169	100	234	True	########	R_3FOjEsiIJnccMr0
########	########	IP Address	168.75.198.241	100	323	True	########	R_WpShND5wfxf3DyN

########	########	IP Address	216.176.1<br>22.160	100	336	True	########	R_2z5Ovw<br>ckqnjO6e<br>R

########	########	IP Address	216.176.100.61	100	910	True	########	R_3qUMhv106OedE0

########	########	IP Address	216.176.1 24.78	100	228	True	########	R_3dX1wk uGQGok4l S

########	########	IP Address	174.202.103.159	100	314	True	########	R_W2l25pNZHqlmjYd

########	########	IP Address	216.176.1<br>01.38	100	263	True	########	R_cUSqqf<br>RBVVnlqjn

########	########	IP Address	216.176.125.145	100	254	True	########	R_1EX9qgtuz1ndMsb
########	########	IP Address	50.121.31.172	100	666	True	########	R_DiyFQsmWY0T9dBL

########	########	IP Address	50.121.28.238	100	203	True	########	R_31MoLIXt4GpduXG

########	########	IP Address	50.104.4.22	100	187	True	########	R_1pxli2I0Ptxcq6k

########	########	IP Address	168.75.198.139	100	285	True	########	R_1gkEglzALytI86a
########	########	IP Address	50.104.21.52	100	13	True	########	R_UMGDw281Wj72mWt

########	########	IP Address	216.176.1<br>00.156	100	233	True	########	R_bOhdO<br>9llpHnzm<br>CZ

########	########	IP Address	216.176.101.87	100	414	True	########	R_1j8TL3QK95tPVfU

########	########	IP Address	174.202.1 01.87	100	1137	True	########	R_PBWgNl Mr4pavKQ F

########	########	IP Address	174.202.104.236	100	425	True	########	R_dhw1SKQdoLj69Il
########	########	IP Address	107.77.207.107	100	246	True	########	R_yEyMdYDfMiGtlC1

########	########	IP Address	216.176.1 23.40	100	432	True	########	R_1GE2K M1El55S7 3l

########	########	IP Address	67.45.32.157	100	878	True	########	R_3O69g8ll2oSAVQ4

########	########	IP Address	50.121.103.58	100	522	True	########	R_2XiFBBcK7mkSVKE

########	########	IP Address	168.75.19 8.37	100	353	True	########	R_yEZBAlQbWlp8gPT
########	########	IP Address	216.176.1 00.44	100	119	True	########	R_25LOBWX4uOik0mU

########	########	IP Address	173.47.57.158	100	221	True	########	R_21pwagpBRCrAhn7
########	########	IP Address	216.176.124.84	100	358	True	########	R_sNk6Di2NyV8T5jH

########	########	IP Address	72.14.106<br>.222	100	369	True	########	R_32Mgh1<br>f3ulxBuRh
########	########	IP Address	192.161.2<br>16.189	100	216	True	########	R_1hMeVY<br>9Y2SEBM<br>6W

########	########	IP Address	168.75.19 8.69	100	395	True	########	R_T6iqUh oDOmsrm Fj
########	########	IP Address	216.176.1 01.222	100	353	True	########	R_1gbCJjX A7xU2ShJ
########	########	IP Address	174.202.1 00.187	100	38	True	########	R_26aK3H vsjj9tbQc

########	########	IP Address	174.202.1 00.187	100	296	True	########	R_2To4w1 U3DEChA 7Q
########	########	IP Address	76.34.16. 33	100	19	True	########	R_1FErHb vAvV9vj56

########	########	IP Address	76.34.16.33	100	153	True	########	R_2Y55o4CvbySwWpU

########	########	IP Address	174.202.9<br>7.46	100	377	True	########	R_1rwC56<br>jinondCqI

########	########	IP Address	162.1.159.115	100	192	True	########	R_31ShqcyCRLwA1CL

########	########	IP Address	168.75.19<br>8.208	100	281	True	########	R_1rAonsf<br>U6RuEUR<br>m
########	########	IP Address	67.7.47.1	100	264	True	########	R_T02xeZt<br>kQ7sJ2gx

########	########	IP Address	108.147.92.76	100	246	True	########	R_1NwPXPkQYtBp2f4
########	########	IP Address	216.176.119.14	100	507	True	########	R_3Eo6CRceOtsAnvU

########	########	IP Address	67.61.140.126	100	265	True	########	R_3KUQVdb9BWV7XXl

########	########	IP Address	50.127.30<br>.229	100	381	True	########	R_OfYyAa<br>7vWxzgCh<br>X

########	########	IP Address	174.202.103.231	100	309	True	########	R_3ps5B2hD8g0PxTX

########	########	IP Address	174.202.98.156	100	239	True	########	R_1EXqfnePwF5IOyO
########	########	IP Address	174.202.100.43	100	389	True	########	R_1qWEKtRm5uGOE83

########	########	IP Address	184.17.59.4	100	903	True	########	R_umqRAb5xUDRfiA9

########	########	IP Address	216.176.101.11	100	343	True	########	R_1GZJTTvIqYHbxLe
########	########	IP Address	174.202.97.248	100	380	True	########	R_6W3CWKVpaiBQPu1

########	########	IP Address	216.176.1 22.125	100	368	True	########	R_3I0oxJd 13D0UmU 6
########	########	IP Address	50.127.32 .65	100	428	True	########	R_WutQB Pj4Uxzwe 9b

########	########	IP Address	216.176.100.112	100	280	True	########	R_2uTzPqjPyAmsEr9
########	########	IP Address	162.1.159.119	100	215	True	########	R_tMNrRqyFYGLd7zz

########	########	IP Address	192.195.230.53	100	256	True	########	R_3EhCrZ6G8AYPaBY
########	########	IP Address	173.47.43.106	100	389	True	########	R_1owokZUIuGrYG2w

########	########	IP Address	216.176.1<br>19.110	100	432	True	########	R_3qW49<br>wM2kXhy<br>8XF

########	########	IP Address	168.75.19<br>8.90	100	335	True	########	R_pzNeos<br>aCLiLNmd<br>r
########	########	IP Address	174.202.1<br>05.41	100	235	True	########	R_3DoPhf<br>ZMc9hVQ<br>Zz

########	########	IP Address	216.176.1 25.82	100	403	True	########	R_393Ga CoZ8mGX oeR

########	########	IP Address	107.77.23 3.211	100	10044	True	########	R_3QFfet RcE4uDzl O

########	########	IP Address	75.211.21 7.6	100	433	True	########	R_1n26N wDVXHUD Xq5

########	########	IP Address	174.202.1 02.140	100	415	True	########	R_Op0mjd rUhWP6Kj L
########	########	Survey Preview		100	3017	True	########	R_3O9frm 3OjTPnAX G
########	########	IP Address	216.21.21 8.45	100	217	True	########	R_2fwjtO3 KHVzsxtJ

########	########	IP Address	216.176.1 01.26	100	307	True	########	R_3dENG nuL6tkCHj H
########	########	IP Address	216.176.1 24.169	100	320	True	########	R_2qxdxS y8GM4cx MP

########	########	IP Address	67.45.32.186	100	577	True	########	R_0MPsuVGYobxw5fr

########	########	IP Address	216.176.1<br>22.160	100	362	True	########	R_2CwbLo<br>6TsEcT9G<br>0
########	########	IP Address	216.252.8<br>.139	100	21	True	########	R_2zzNoL<br>HxIEQxPu<br>8

########	########	IP Address	172.78.25 3.95	100	580	True	########	R_3gYoF0 P1nRfbnc H

########	########	IP Address	216.176.101.187	100	339	True	########	R_6nVjFrqjQx1FvfX
########	########	IP Address	184.16.138.116	100	417	True	########	R_1LMcLbIFbSTAWQS

########	########	IP Address	216.176.100.27	100	316	True	########	R_3ndXOMyrqckAtr9

########	########	IP Address	216.176.101.78	100	430	True	########	R_PvS7hCCCNFUD2tb

########	########	IP Address	174.202.97.176	100	427	True	########	R_10Pkp30tjY4oVuu

########	########	IP Address	165.139.1<br>91.130	100	815	True	########	R_3QXnG<br>WyrFFX9X<br>o4

########	########	IP Address	184.16.110.145	100	230	True	########	R_1OlS9X0V1bOBiuu
########	########	IP Address	184.53.32.32	100	303	True	########	R_2RNX13mxcojR8hm

########	########	IP Address	168.75.19 8.69	100	482	True	########	R_vrATnq MLiyMJdV D

########	########	IP Address	168.75.19<br>8.74	100	239	True	########	R_zdQ3sb<br>LfdvAo40h

########	########	IP Address	216.176.1 22.80	100	394	True	########	R_3iR3mv fkmZ9k8y E

########	########	IP Address	216.176.1 24.120	100	355	True	########	R_Td3RKb VbzHAc1A 5

########	########	IP Address	192.174.101.177	100	553	True	########	R_BFm4tsi2fSkqZpf

########	########	IP Address	172.243.1 01.41	100	634	True	########	R_2Qu0ds fMJu20Kw Y
########	########	IP Address	174.202.1 00.207	100	313	True	########	R_2bHnD Elys6eLviL

########	########	IP Address	174.202.100.10	100	349	True	########	R_3CDe0MWGr5aCpB7

########	########	IP Address	172.77.90.124	100	282	True	########	R_2sYeTam3LZ3KvJT
########	########	IP Address	174.202.97.227	100	189	True	########	R_1M3TLJqWVn7UsBb

########	########	IP Address	168.75.198.107	100	196	True	########	R_12a51OdGWI7xriA

########	########	IP Address	216.176.1 25.31	100	297	True	########	R_2Ez5pdI XyVP1STq

########	########	IP Address	98.97.8.253	100	218	True	########	R_31QwPI8vFDHjjRG

########	########	IP Address	166.205.124.80	100	474	True	########	R_10x5dIHiHhFkh1p

########	########	IP Address	184.20.40.72	100	256	True	########	R_20MOcrhkg3kYjNG

########	########	IP Address	107.77.209.158	100	284	True	########	R_30wwhACUchFF0i2
########	########	IP Address	184.62.189.39	100	493	True	########	R_3sndh9ZwsRFqQtJ

########	########	IP Address	174.202.107.133	100	661	True	########	R_308gAkQQaQr5Jmn

########	########	IP Address	174.202.103.3	100	342	True	########	R_2CHwlYwWpKsz51k

########	########	IP Address	184.16.87.146	100	1435	True	########	R_2bZxu94dpjUGCqq

########	########	IP Address	168.75.19 8.237	100	402	True	########	R_2dseWf bJJVH4iPn

########	########	IP Address	216.176.100.6	100	520	True	########	R_3ixx9GFLFqrE1ZD

########	########	IP Address	184.16.13 5.199	100	710	True	########	R_AyAt8Ez 6TPP5zvb

########	########	IP Address	192.174.96.219	100	175	True	########	R_0BV7e8omHxZOPjX
########	########	IP Address	174.202.104.181	100	359	True	########	R_1I6hWyNM0NlSypt
########	########	IP Address	50.121.8.214	100	273	True	########	R_3C8CZqm06ipKakx

########	########	IP Address	50.104.20.26	100	422	True	########	R_3HTAlKL7CbNrEPn
########	########	IP Address	192.174.96.219	100	249	True	########	R_2uOpnQklIOPzH26

########	########	IP Address	216.176.100.149	100	408	True	########	R_33figFuBmbYXZdC
########	########	IP Address	174.202.97.245	8	12	False	########	R_0IIR0TWeewQbwDD
########	########	IP Address	50.104.13.133	67	93	False	########	R_uxMH2yVOIBaeMBX

########	########	IP Address	24.142.76.234	89	208	False	########	R_2xPqspJC3E0PesA
########	########	IP Address	50.102.200.108	25	288	False	########	R_ByTc0V6mVto0QiR
########	########	IP Address	75.211.219.89	53	294	False	########	R_0dkpdNT63JNQbbr
########	########	IP Address	174.202.98.125	78	160	False	########	R_2CpKlqaubTOtRRe
########	########	IP Address	216.176.101.197	25	92	False	########	R_1DOlWeTsFI5XMYL
########	########	IP Address	184.16.92.89	53	148	False	########	R_1Nwtti2pubv9Urm

########	########	IP Address	168.75.19 8.67	19	51	False	########	R_3HtxdQ fBDxTXlW 2
########	########	IP Address	216.176.1 24.207	3	27	False	########	R_s4HFU M4c3Z4m t9L
########	########	IP Address	38.124.12 7.150	67	1466	False	########	R_1f2AWb zMe4uXQ bJ
########	########	IP Address	216.176.1 01.152	100	234	True	########	R_27a3oM arzYMCW dG

########	########	IP Address	159.218.128.18	78	184	False	########	R_3qmU1OAesmgy4t
########	########	IP Address	168.75.198.77	78	128	False	########	R_puETunIP6oUDN1T
########	########	IP Address	24.142.76.252	53	163	False	########	R_1cZ0BAuT642YgDk
########	########	IP Address	108.147.92.57	75	254	False	########	R_1DNg28AUCiLphfn

########	########	IP Address	50.127.92.193	25	43	False	########	R_22Mt1SerU07ATxu
########	########	IP Address	50.121.42.141	100	723	True	########	R_2BycfDEvKkLXrPE
########	########	IP Address	174.202.106.142	25	120	False	########	R_1i9Z7VaNUPDSavt
########	########	IP Address	174.202.105.190	6	16	False	########	R_s55gzjHmTjAAmnn
########	########	IP Address	216.176.107.39	25	50	False	########	R_zUTYTXuCF85RT33

########	########	IP Address	192.174.9 9.91	100	306	True	########	R_AjwkXJe Kl3szkkh

########	########	IP Address	216.176.1 23.230	100	577	True	########	R_1kSIELB fGBCmUT 2
########	########	IP Address	208.184.2 49.20	3	18	False	########	R_2xShVv 1Kg2OxHV Y

########	########	IP Address	216.176.101.35	100	184	True	########	R_3GBrpsMcLRLU3tl
########	########	IP Address	216.176.102.229	8	26	False	########	R_30xl5iS8XKU5lmq

########	########	IP Address	216.176.1 00.36	100	218	True	########	R_3hDQd KpoluguG 77
########	########	IP Address	216.176.1 16.92	25	60	False	########	R_CdzqD9 kBkKoAjW 9
########	########	IP Address	174.202.9 7.203	25	54	False	########	R_21teuH Ujc9koCh 0

########	########	IP Address	216.176.101.108	100	235	True	########	R_1rBbS3KfgnSzvKe
########	########	IP Address	23.247.137.76	6	18	False	########	R_9oWl3ZImEGr9psJ
########	########	IP Address	174.202.104.222	100	17	True	########	R_129yFLvN1FbHetu
########	########	IP Address	192.174.96.178	100	599	True	########	R_1pXSWRrFScSlpNs

########	########	IP Address	174.214.48.96	8	23	False	########	R_3FQlmmXhzT5qr2x
########	########	IP Address	216.21.220.25	25	75	False	########	R_270PGoOZXVsT0iH
########	########	IP Address	172.78.243.14	39	82	False	########	R_1rBLKBUY54fQwrG
########	########	IP Address	67.45.32.122	78	285	False	########	R_1GUoL5hI33wJDfC

########	########	IP Address	174.202.1<br>02.209	100	521	True	########	R_1GwSvJ<br>w7TMxdyp<br>8
########	########	IP Address	168.75.19<br>8.91	25	28	False	########	R_ALJqum<br>unyt7F3gd

########	########	IP Address	38.86.84.110	100	283	True	########	R_1QDykYcq3NAIri4

########	########	IP Address	174.202.9<br>8.53	100	192	True	########	R_brYN3af<br>ZAE0IwbT

########	########	IP Address	172.78.23<br>7.218	100	679	True	########	R_1QtEcS<br>FHqrqmgT<br>1

########	########	IP Address	155.190.1<br>9.6	100	184	True	########	R_1q8w4R<br>3V6OYXX<br>AF

########	########	IP Address	174.202.9<br>9.116	100	617	True	########	R_ONkkC<br>Sw32UA6<br>kpj

########	########	IP Address	199.10.16.202	100	1519	True	########	R_4Gc8hzB948mhNyF

########	########	IP Address	192.174.103.92	100	302	True	########	R_12FkxZU6QnVQOmV

########	########	IP Address	24.142.76.108	100	426	True	########	R_25WS6oq0FRT22tx
########	########	IP Address	174.202.100.155	89	158	False	########	R_qUEcgD65CVdJVyp

########	########	IP Address	63.134.14 1.71	100	223	True	########	R_2U5fV WHjPPvj9 HD
########	########	IP Address	63.134.14 1.71	100	167	True	########	R_24HGTb m3yKIZSY H

########	########	IP Address	63.134.14<br>1.71	100	206	True	########	R_25Gwx<br>m9C7yWh<br>Gbs

########	########	IP Address	63.134.14<br>1.71	100	324	True	########	R_1nTpq3<br>H8ezmDD<br>ST

########	########	IP Address	63.134.14 1.71	100	246	True	########	R_yUVzMR QUXV4G0 Rr

########	########	IP Address	63.134.14<br>1.71	100	211	True	########	R_21jFr50<br>d65AZC40

########	########	IP Address	63.134.14<br>1.71	100	159	True	########	R_1igkMUi<br>JLxcz0W8

########	########	IP Address	63.134.14 1.71	100	245	True	########	R_OIFJ1Ce2noSLA1b
########	########	IP Address	63.134.14 1.71	100	267	True	########	R_2fKawbIoDRdJvtB

########	########	IP Address	63.134.141.71	100	194	True	########	R_1LbPue2X75mfg2c
########	########	IP Address	63.134.141.71	100	262	True	########	R_Ybufwj4p9jKaoSt
########	########	IP Address	63.134.141.71	100	386	True	########	R_3eoXeJFD5D9ztfj

########	########	IP Address	63.134.14 1.71	100	100	True	########	R_3FLjqs2 frvRixWZ
########	########	IP Address	63.134.14 1.71	100	214	True	########	R_211j6JX dRvcnYMy
########	########	IP Address	63.134.14 1.71	100	307	True	########	R_STwJCP sh2KTS3a 9

########	########	IP Address	63.134.14<br>1.71	100	159	True	########	R_PUtYbQ<br>Kzbg7Bm<br>DL

########	########	IP Address	63.134.14<br>1.71	100	183	True	########	R_24xgVH<br>D6EQ1l3<br>WD

########	########	IP Address	63.134.14<br>1.71	100	159	True	########	R_7WFcl<br>Wnh41K8<br>sBr
########	########	IP Address	103.188.2<br>34.171	42	481	False	########	R_4KHkzG<br>8Pvynvlyd

LocationLa	LocationLo	Distributio	UserLangu	Q4	Q5	Q6	Q7	Q35	Q13	Q37	Q10	Q10_13_TE
Location La	Location Lo	Distributio	User Langu	Do you live	What is you	Which of th	What is you	How are yo	Do you hav	How would	Who is you	Who is you
38.6717	-87.5128	anonymous	EN	Yes	24	Male	greater than $120,000	At home on your phone/computer etc.	Yes	Multiple times daily	Frontier Communications	

38.6624	-86.916	anonymous	EN	Yes	35	Female	$60,000 - $89,999	At home on your phone/computer etc.	Yes	Multiple times daily	RTC

22.2842	114.1759	anonymous	EN	Yes	24	Male	$60,000 - $89,999	Public setting on your phone/computer etc.	Yes	Multiple times daily	Viasat

38.6529	-87.1699	anonymous	EN	Yes	55	Female	greater than $120,000	At home on your phone/computer etc.	Yes	Multiple times daily	RTC

38.7715	-87.3222	anonymous	EN	Yes	49	Female	$90,000 - $119,999	Public setting on your phone/computer etc.	Yes	Multiple times daily	Viasat

38.6752	-86.7747	anonymous	EN	Yes	55	Female	$90,000 - $119,999	At home on your phone/computer etc.	Yes	Multiple times daily	Smithville

39.7684	-86.158	anonymous	EN	Yes	64	Female	$30,000 - $59,999	At home on your phone/computer etc.	Yes	Multiple times daily	T-Mobile

39.2051	-85.9324	anonymous	EN	Yes	29	Female	greater than $120,000	At home on your phone/computer etc.	Yes	Multiple times daily	Other (please specify)	Sparklight

39.1215	-86.5784	anonymous	EN	Yes	25	Female	$60,000 - $89,999	Public setting on your phone/computer etc.	Yes	Multiple times daily	T-Mobile

40.7908	-85.8327	anonymous	EN	Yes	56	Female	$90,000 - $119,999	At home on your phone/computer etc.	Yes	Multiple times daily	Frontier Communications

39.5177	-87.1238	anonymous	EN	Yes	42		Female	$90,000 - $119,999	At home on your phone/computer etc.	Yes	Multiple times daily	Frontier Communications

39.5177	-87.1238	anonymous	EN	Yes	53	Male	greater than $120,000	At home on your phone/computer etc.	Yes	Multiple times daily	Frontier Communications

39.0021	-85.627	anonymous	EN	Yes	58	Female		Public setting on your phone/computer etc.	Yes	Multiple times daily	Frontier Communications

38.6529	-87.1699	anonymous	EN	Yes	23	Female	$90,000 - $119,999	At home on your phone/computer etc.	Yes	Multiple times daily	Other (please specify)	Sparklight

33.7485	-84.3871	anonymous	EN	Yes	42	Female	$30,000 - $59,999	At home on your phone/computer etc.	Yes	Multiple times daily	Hughes Net

39.5177	-87.1238	anonymous	EN	Yes	63	Female	$90,000 - $119,999	At home on your phone/computer etc.	Yes	Multiple times daily	Other (please specify)	Frontier
41.0147	-85.0974	anonymous	EN	Yes	69	Female	$30,000 - $59,999	At home on your phone/computer etc.	Yes	Multiple times daily	Frontier Communications	

39.2051	-85.9324	anonymous	EN	Yes	43	Female	greater than $120,000	At home on your phone/computer etc.	Yes	Multiple times daily	Cell phone company
38.7735	-86.6285	anonymous	EN	Yes	73	Female	$30,000 - $59,999	At home on your phone/computer etc.	Yes	Multiple times daily	RTC

41.8719	-87.6589	anonymous	EN	Yes	26	Female	greater than $120,000	At home on your phone/computer etc.	Yes	Multiple times daily	RTC

41.3458	-85.4155	anonymous	EN	Yes	39	Female	$90,000 - $119,999	Public setting on your phone/computer etc.	Yes	Multiple times daily	Frontier Communications

38.7735	-86.6285	anonymous	EN	Yes		Female	$90,000 - $119,999	At home on your phone/computer etc.	Yes	Multiple times daily	RTC
38.6624	-86.916	anonymous	EN	Yes	35	Female	$30,000 - $59,999	At home on your phone/computer etc.	Yes	Multiple times daily	RTC

38.6529	-87.1699	anonymous	EN	Yes	50	Female	$90,000 - $119,999	At home on your phone/computer etc.	Yes	Multiple times daily	Frontier Communications

38.6624	-86.916	anonymous	EN	Yes	66	Female	$60,000 - $89,999	At home on your phone/computer etc.	Yes	Multiple times daily	RTC

39.5177	-87.1238	anonymous	EN	Yes	51	Female	greater than $120,000	At home on your phone/computer etc.	Yes	Multiple times daily	Frontier Communications

37.9606	-87.5333	anonymous	EN	Yes	51	Female	$90,000 - $119,999	At home on your phone/computer etc.	Yes	Multiple times daily	Frontier Communications
38.6529	-87.1699	anonymous	EN	No							

38.6529	-87.1699	anonymous	EN	Yes	50	Female	$60,000 - $89,999	At home on your phone/computer etc.	Yes	Multiple times daily	RTC

38.6529	-87.1699	anonymous	EN	Yes	47	Female	$30,000 - $59,999	At home on your phone/computer etc.	Yes	Multiple times daily	Other (please specify)	Sparklight
38.6624	-86.916	anonymous	EN	Yes	30	Female	$60,000 - $89,999	At home on your phone/computer etc.	Yes	Multiple times daily	RTC	

39.2318	-85.8884	anonymous	EN	No								
38.655	-87.0509	anonymous	EN	Yes	43	Female	$90,000 - $119,999	At home on your phone/computer etc.	Yes	Multiple times daily	RTC	

41.8874	-87.6318	anonymous	EN	Yes	34	Female	greater than $120,000	At home on your phone/computer etc.	Yes	Multiple times daily	Other (please specify)	Starlink

38.6752	-86.7747	anonymous	EN	Yes	67	Female	less than $30,000	At home on your phone/computer etc.	Yes	Multiple times daily	RTC
38.6624	-86.916	anonymous	EN	Yes	37	Male	greater than $120,000	At home on your phone/computer etc.	Yes	Multiple times daily	RTC
38.6529	-87.1699	anonymous	EN	Yes	27	Female	$60,000 - $89,999	At home on your phone/computer etc.	Yes	Multiple times daily	Cable One

39.2051	-85.9324	anonymous	EN	Yes	22	Male	$30,000 - $59,999	Public setting on your phone/computer etc.	Yes	Multiple times daily	RTC

39.2051	-85.9324	anonymous	EN	Yes	29	Male	greater than $120,000	Public setting on your phone/computer etc.	Yes	Multiple times daily	Frontier Communications

37.9606	-87.5333	anonymous	EN	Yes	66	Female	$30,000 - $59,999	At home on your phone/computer etc.	Yes	Multiple times daily	Frontier Communications
38.6529	-87.1699	anonymous	EN	Yes	39	Male	greater than $120,000	At home on your phone/computer etc.	Yes	Multiple times daily	Cell phone company

39.7684	-86.158	anonymous	EN	Yes	28	Male	$90,000 - $119,999	At home on your phone/computer etc.	Yes	Multiple times daily	T-Mobile

38.6717	-87.5128	anonymous	EN	Yes	47	Female	greater than $120,000	At home on your phone/computer etc.	Yes	Multiple times daily	Other (please specify)	Private

41.8874	-87.6318	anonymous	EN	Yes	27	Female	greater than $120,000	At home on your phone/computer etc.	Yes	Multiple times daily	Other (please specify)	Starlink

38.6529	-87.1699	anonymous	EN	Yes	60	Female	\$30,000 - \$59,999	At home on your phone/computer etc.	Yes	Multiple times daily	Other (please specify)	Sparklight

38.7735	-86.6285	anonymous	EN	Yes	58	Female	$60,000 - $89,999	At home on your phone/computer etc.	Yes	Multiple times daily	RTC

38.6752	-86.7747	anonymous	EN	Yes	65	Female	$30,000 - $59,999	At home on your phone/computer etc.	Yes	Multiple times daily	RTC

38.745	-86.4803	anonymous	EN	Yes	31	Female	$60,000 - $89,999	At home on your phone/computer etc.	Yes	Multiple times daily	Frontier Communications
38.6529	-87.1699	anonymous	EN	Yes	65	Female	$60,000 - $89,999	At home on your phone/computer etc.	Yes	Multiple times daily	RTC

38.6624	-86.916	anonymous	EN	Yes	68	Female	$60,000 - $89,999	At home on your phone/computer etc.	Yes	Multiple times daily	RTC

38.6529	-87.1699	anonymous	EN	Yes	64	Female	$60,000 - $89,999	At home on your phone/computer etc.	Yes	Multiple times daily	Other (please specify)	Sparklight

38.7735	-86.6285	anonymous	EN	Yes	51	Female	$30,000 - $59,999	At home on your phone/computer etc.	Yes	Multiple times daily	RTC
39.5324	-87.3275	anonymous	EN	Yes	68	Female	$60,000 - $89,999	At home on your phone/computer etc.	Yes	Multiple times daily	Cell phone company

38.6529	-87.1699	anonymous	EN	Yes	41	Female	$60,000 - $89,999	At home on your phone/computer etc.	Yes	Multiple times daily	Other (please specify)	Spark light

38.6624	-86.916	anonymous	EN	Yes	57	Female	greater than $120,000	At home on your phone/computer etc.	Yes	Multiple times daily	RTC

38.6624	-86.916	anonymous	EN	Yes	46	Female	greater than $120,000	At home on your phone/computer etc.	Yes	Multiple times daily	RTC
38.6717	-87.5128	anonymous	EN	Yes	50	Female	$30,000 - $59,999	At home on your phone/computer etc.	Yes	Multiple times daily	Cell phone company

41.1204	-85.8688	anonymous	EN	Yes	51	Female	$90,000 - $119,999	At home on your phone/computer etc.	Yes	Multiple times daily	Frontier Communications

38.6717	-87.5128	anonymous	EN	Yes	65	Male	greater than $120,000	At home on your phone/computer etc.	Yes	Multiple times daily	Cell phone company

38.6624	-86.916	anonymous	EN	Yes	44	Male	greater than $120,000	At home on your phone/computer etc.	Yes	Multiple times daily	RTC

39.4057	-87.4026	anonymous	EN	Yes	29		Female	$90,000 - $119,999	Public setting on your phone/computer etc.	Yes	Multiple times daily	Frontier Communications

38.7613	-85.4066	anonymous	EN	Yes	64	Female	less than $30,000	At home on your phone/computer etc.	Yes	Multiple times daily	Frontier Communications

38.6624	-86.916	anonymous	EN	Yes	71	Female	$60,000 - $89,999	At home on your phone/computer etc.	Yes	Multiple times daily	RTC

38.6624	-86.916	anonymous	EN	Yes	75		Female	less than $30,000	At home on your phone/computer etc.	Yes	Multiple times daily	RTC

38.6529	-87.1699	anonymous	EN	Yes	18	Female	$60,000 - $89,999	At home on your phone/computer etc.	Yes	Multiple times daily	RTC

38.6624	-86.916	anonymous	EN	Yes	57	Female	$90,000 - $119,999	At home on your phone/computer etc.	Yes	Multiple times daily	RTC
38.6529	-87.1699	anonymous	EN	Yes	34	Male	greater than $120,000	At home on your phone/computer etc.	Yes	Multiple times daily	Cable One

38.6529	-87.1699	anonymous	EN	Yes	46		Female	$60,000 - $89,999	At home on your phone/computer etc.	Yes	Multiple times daily	Other (please specify)	Sparklite

38.6624	-86.916	anonymous	EN	Yes	56	Male	greater than $120,000	At home on your phone/computer etc.	Yes	Multiple times daily	RTC

38.6624	-86.916	anonymous	EN	Yes	35	Female	greater than $120,000	At home on your phone/computer etc.	Yes	Multiple times daily	RTC	

38.6624	-86.916	anonymous	EN	Yes	71	Female	$30,000 - $59,999	At home on your phone/computer etc.	Yes	Multiple times a week	RTC
39.5177	-87.1238	anonymous	EN	Yes	42	Male	greater than $120,000	At home on your phone/computer etc.	Yes	Multiple times daily	Frontier Communications

38.9483	-86.6129	anonymous	EN	Yes	35	Female	$60,000 - $89,999	At home on your phone/computer etc.	Yes	Multiple times daily	RTC
35.2296	-80.843	anonymous	EN	Yes	49	Female	$30,000 - $59,999	At home on your phone/computer etc.	No		

41.3479	-85.1398	anonymous	EN	Yes	55	Female	$30,000 - $59,999	At home on your phone/computer etc.	Yes	Multiple times daily	Frontier Communications	
39.2051	-85.9324	anonymous	EN	Yes	24	Male	$30,000 - $59,999	Public setting on your phone/computer etc.	Yes	Multiple times daily	Other (please specify)	Frontier
38.6624	-86.916	anonymous	EN	Yes	44	Female	greater than $120,000	At home on your phone/computer etc.	Yes	Multiple times daily	RTC	

38.745	-86.4803	anonymous	EN	Yes	38	Female	greater than $120,000	At home on your phone/computer etc.	Yes	Multiple times daily	RTC
38.2539	-85.76	anonymous	EN	No							

38.6624	-86.916	anonymous	EN	Yes	19	Female	less than $30,000	At home on your phone/computer etc.	Yes	Multiple times daily	Unsure
38.6529	-87.1699	anonymous	EN	Yes	52	Male	$90,000 - $119,999	At home on your phone/computer etc.	Yes	Multiple times daily	RTC

38.6624	-86.916	anonymous	EN	Yes	54	Male	$60,000 - $89,999	Public setting on your phone/computer etc.	Yes	Multiple times daily	RTC
41.8486	-87.6288	anonymous	EN	Yes	44	Male	$90,000 - $119,999	Public setting on your phone/computer etc.	Yes	Multiple times daily	Cell phone company
38.6624	-86.916	anonymous	EN	Yes	52	Female	$60,000 - $89,999	At home on your phone/computer etc.	Yes	Multiple times daily	Cable One

39.2998	-85.4918	anonymous	EN	Yes	55	Female	greater than $120,000	At home on your phone/computer etc.	Yes	Multiple times daily	Cell phone company

39.2051	-85.9324	anonymous	EN	Yes	61	Female	$60,000 - $89,999	At home on your phone/computer etc.	Yes	Multiple times daily	Unsure

38.6624	-86.916	anonymous	EN	Yes	63	Male	$90,000 - $119,999	At home on your phone/computer etc.	Yes	Multiple times daily	RTC

38.6529	-87.1699	anonymous	EN	Yes	23	Female	greater than $120,000	At home on your phone/computer etc.	Yes	Multiple times daily	RTC
39.4057	-87.4026	anonymous	EN	Yes	65	Female	$90,000 - $119,999	At home on your phone/computer etc.	Yes	Multiple times daily	Cell phone company

38.745	-86.4803	anonymous	EN	Yes	57	Female	$60,000 - $89,999	At home on your phone/computer etc.	Yes	Multiple times daily	Other (please specify)	Spark light
40.9554	-85.011	anonymous	EN	Yes	38	Female	$60,000 - $89,999	At home on your phone/computer etc.	Yes	Multiple times daily	RTC	

38.6624	-86.916	anonymous	EN	Yes	24	Female	$60,000 - $89,999	At home on your phone/computer etc.	Yes	Multiple times daily	Frontier Communications

38.6529	-87.1699	anonymous	EN	Yes	30	Female	less than $30,000	At home on your phone/computer etc.	Yes	Multiple times daily	Other (please specify)	Sparklight
38.6624	-86.916	anonymous	EN	Yes	41	Male	greater than $120,000	At home on your phone/computer etc.	Yes	Multiple times daily	RTC	

38.6624	-86.916	anonymous	EN	Yes	27	Male	greater than $120,000	Public setting on your phone/computer etc.	Yes	Multiple times daily	RTC

38.6529	-87.1699	anonymous	EN	Yes	41		Female	greater than $120,000	At home on your phone/computer etc.	Yes	Multiple times daily	Unsure

39.1554	-90.815	anonymous	EN	Yes	52	Female	$60,000 - $89,999	Public setting on your phone/computer etc.	Yes	Multiple times daily	Other (please specify)	Exceed
41.8502	-87.6736	anonymous	EN	Yes	43	Female	greater than $120,000	At home on your phone/computer etc.	Yes	Multiple times daily	RTC	

39.2051	-85.9324	anonymous	EN	Yes	71	Female	less than $30,000	At home on your phone/computer etc.	No

38.6624	-86.916	anonymous	EN	Yes	51	Female	$30,000 - $59,999	At home on your phone/computer etc.	Yes	Multiple times daily	RTC
38.6624	-86.916	anonymous	EN	Yes	69	Female	less than $30,000	At home on your phone/computer etc.	Yes	Multiple times daily	RTC

38.6752	-86.7747	anonymous	EN	Yes	54	Female	$90,000 - $119,999	At home on your phone/computer etc.	Yes	Multiple times daily	RTC

38.6624	-86.916	anonymous	EN	Yes	79	Female	$30,000 - $59,999	At home on your phone/computer etc.	Yes	Multiple times daily	RTC

38.6752	-86.7747	anonymous	EN	Yes	45	Female	$30,000 - $59,999	At home on your phone/computer etc.	Yes	Multiple times daily	RTC	

39.2051	-85.9324	anonymous	EN	Yes	27	Female	$60,000 - $89,999	At home on your phone/computer etc.	No

38.6624	-86.916	anonymous	EN	Yes	30	Female	$90,000 - $119,999	At home on your phone/computer etc.	Yes	Multiple times daily	RTC

40.9554	-85.011	anonymous	EN	Yes	52	Female	$30,000 - $59,999	At home on your phone/computer etc.	Yes	Multiple times daily	RTC
39.3724	-86.1801	anonymous	EN	Yes	62	Male	$90,000 - $119,999	At home on your phone/computer etc.	Yes	Multiple times a month	Frontier Communications

39.5177	-87.1238	anonymous	EN	Yes	61	Female	$30,000 - $59,999	At home on your phone/computer etc.	Yes	Multiple times daily	Frontier Communications

39.5177	-87.1238	anonymous	EN	Yes	42	Female	greater than $120,000	Public setting on your phone/computer etc.	Yes	Multiple times daily	Frontier Communications

38.6624	-86.916	anonymous	EN	Yes	66	Female	$30,000 - $59,999	At home on your phone/computer etc.	Yes	Multiple times daily	RTC
39.1732	-87.3941	anonymous	EN	No							

38.6624	-86.916	anonymous	EN	Yes	33	Female	greater than $120,000	At home on your phone/computer etc.	Yes	Multiple times daily	RTC

38.6624	-86.916	anonymous	EN	Yes	27	Female	$30,000 - $59,999	Public setting on your phone/computer etc.	Yes	Multiple times a week	RTC

39.2051	-85.9324	anonymous	EN	Yes	68	Female	$60,000 - $89,999	At home on your phone/computer etc.	Yes	Multiple times daily	Cell phone company

39.2051	-85.9324	anonymous	EN	Yes	76		Female	less than $30,000	Public setting on your phone/computer etc.	Yes	Multiple times daily	Cell phone company
41.8764	-87.6133	anonymous	EN	Yes	23		Female	$30,000 - $59,999	At home on your phone/computer etc.	Yes	Multiple times daily	Cell phone company

40.9554	-85.011	anonymous	EN	Yes	56	Female	$30,000 - $59,999	At home on your phone/computer etc.	Yes	Multiple times daily	RTC	

33.7485	-84.3871	anonymous	EN	Yes	29	Female	greater than $120,000	At home on your phone/computer etc.	Yes	Multiple times daily	Hughes Net

39.5177	-87.1238	anonymous	EN	Yes	54	Female	$60,000 - $89,999	At home on your phone/computer etc.	Yes	Multiple times daily	Frontier Communications	

38.6624	-86.916	anonymous	EN	Yes	55	Female	$90,000 - $119,999	Public setting on your phone/computer etc.	Yes	Multiple times daily	RTC
38.6624	-86.916	anonymous	EN	Yes	24	Female	$60,000 - $89,999	At home on your phone/computer etc.	Yes	Multiple times daily	RTC

38.6529	-87.1699	anonymous	EN	Yes	45	Male	$30,000 - $59,999	Public setting on your phone/computer etc.	Yes	Multiple times daily	Greenwell
38.6752	-86.7747	anonymous	EN	Yes	36	Male	$60,000 - $89,999	At home on your phone/computer etc.	Yes	Multiple times daily	RTC

36.6089	-88.3031	anonymous	EN	Yes	21	Female	$60,000 - $89,999	At home on your phone/computer etc.	Yes	Multiple times a month	Frontier Communications
38.745	-86.4803	anonymous	EN	Yes	29	Female	$30,000 - $59,999	Public setting on your phone/computer etc.	Yes	Multiple times daily	Frontier Communications

38.6624	-86.916	anonymous	EN	Yes	66	Male	greater than $120,000	At home on your phone/computer etc.	Yes	Multiple times daily	RTC
38.6624	-86.916	anonymous	EN	Yes	74	Male	$30,000 - $59,999	Public setting on your phone/computer etc.	Yes	Multiple times daily	RTC
39.2051	-85.9324	anonymous	EN	No							

39.2051	-85.9324	anonymous	EN	Yes	26	Male	$30,000 - $59,999	Public setting on your phone/computer etc.	Yes	Multiple times daily	Cell phone company
38.0086	-87.5746	anonymous	EN	No							

38.0086	-87.5746	anonymous	EN	Yes	19	Female	greater than $120,000	Public setting on your phone/computer etc.	Yes	Multiple times daily	RTC

39.2318	-85.8884	anonymous	EN	Yes	18		Female	$90,000 - $119,999	Public setting on your phone/computer etc.	Yes	Multiple times daily	Cell phone company

39.786	-86.1589	anonymous	EN	Yes	36	Female	$60,000 - $89,999	Public setting on your phone/computer etc.	Yes	Multiple times daily	Cell phone company	

38.6624	-86.916	anonymous	EN	Yes	78		Female	$30,000 - $59,999	Public setting on your phone/computer etc.	Yes	Multiple times daily	RTC
41.1622	-85.4724	anonymous	EN	Yes	52		Female	$90,000 - $119,999	Public setting on your phone/computer etc.	Yes	Multiple times daily	Frontier Communications

41.8486	-87.6288	anonymous	EN	Yes	69	Female	$30,000 - $59,999	Public setting on your phone/computer etc.	Yes	Multiple times daily	Other (please specify)	Sparklight
38.6624	-86.916	anonymous	EN	Yes	47	Female	greater than $120,000	At home on your phone/computer etc.	Yes	Multiple times daily	RTC	

38.7715	-87.3222	anonymous	EN	Yes	38	Female	$60,000 - $89,999	Public setting on your phone/computer etc.	Yes	Multiple times daily	Unsure

41.0147	-85.0974	anonymous	EN	Yes	56	Female	less than $30,000	Public setting on your phone/computer etc.	Yes	Multiple times daily	Other (please specify)	Dish master

39.2051	-85.9324	anonymous	EN	Yes	39	Female	$60,000 - $89,999	Public setting on your phone/computer etc.	Yes	Multiple times daily	Other (please specify)	New wave

39.2051	-85.9324	anonymous	EN	Yes	53	Female	greater than $120,000	Public setting on your phone/computer etc.	Yes	Multiple times daily	Cell phone company
39.2051	-85.9324	anonymous	EN	Yes	66	Female	greater than $120,000	At home on your phone/computer etc.	Yes	Multiple times daily	Cell phone company

39.6421	-85.1439	anonymous	EN	Yes	51		Female	$60,000 - $89,999	Public setting on your phone/computer etc.	Yes	Multiple times daily	Other (please specify)	Sparklight

38.6624	-86.916	anonymous	EN	Yes	37	Female	$90,000 - $119,999	At home on your phone/computer etc.	Yes	Multiple times daily	RTC
39.2318	-85.8884	anonymous	EN	Yes	64	Male	$30,000 - $59,999	At home on your phone/computer etc.	Yes	Multiple times daily	Cell phone company

38.6752	-86.7747	anonymous	EN	Yes	74	Female	$30,000 - $59,999	At home on your phone/computer etc.	Yes	Multiple times daily	RTC
40.8771	-85.5045	anonymous	EN	Yes	75	Female	less than $30,000	At home on your phone/computer etc.	Yes	Multiple times daily	Frontier Communications

38.6624	-86.916	anonymous	EN	Yes	60	Female	$30,000 - $59,999	At home on your phone/computer etc.	Yes	Multiple times daily	RTC
39.786	-86.1589	anonymous	EN	Yes	37	Female	greater than $120,000	Public setting on your phone/computer etc.	Yes	Multiple times daily	RTC

37.9606	-87.5333	anonymous	EN	Yes	21	Female	less than $30,000	Public setting on your phone/computer etc.	Yes	Multiple times daily	Cell phone company	
38.6529	-87.1699	anonymous	EN	Yes	85	Female	less than $30,000	At home on your phone/computer etc.	Yes	Multiple times daily	Other (please specify)	Sparklight

38.6624	-86.916	anonymous	EN	Yes	56	Female	$30,000 - $59,999	At home on your phone/computer etc.	Yes	Multiple times daily	RTC

38.6624	-86.916	anonymous	EN	Yes	68	Female	$90,000 - $119,999	At home on your phone/computer etc.	Yes	Multiple times a week	RTC
37.9606	-87.5333	anonymous	EN	Yes	18	Female	less than $30,000	Public setting on your phone/computer etc.	Yes	Multiple times daily	RTC

38.6752	-86.7747	anonymous	EN	Yes	26	Female	$30,000 - $59,999	At home on your phone/computer etc.	Yes	Multiple times daily	RTC

37.751	-97.822	anonymous	EN	Yes	26	Female	less than $30,000	Public setting on your phone/computer etc.	Yes	Multiple times daily	Cell phone company

39.4956	-87.4651	anonymous	EN	Yes	59	Female	$90,000 - $119,999	At home on your phone/computer etc.	Yes	Multiple times daily	Other (please specify)	Verizon

39.1215	-86.5784	anonymous	EN	Yes	38	Female	$90,000 - $119,999	Public setting on your phone/computer etc.	Yes	Multiple times daily	Hughes Net	
38.6717	-87.5128	preview	EN									
38.538	-86.6121	anonymous	EN	Yes	38	Male	$30,000 - $59,999	Public setting on your phone/computer etc.	Yes	Multiple times daily	Other (please specify)	Verizon

38.6624	-86.916	anonymous	EN	Yes	36	Female	$90,000 - $119,999	At home on your phone/computer etc.	Yes	Multiple times daily	RTC
38.7735	-86.6285	anonymous	EN	Yes	48	Female	greater than $120,000	At home on your phone/computer etc.	Yes	Multiple times daily	RTC

33.7485	-84.3871	anonymous	EN	Yes	51	Female	$90,000 - $119,999	At home on your phone/computer etc.	Yes	Multiple times daily	Hughes Net

38.6752	-86.7747	anonymous	EN	Yes	26	Female	$30,000 - $59,999	At home on your phone/computer etc.	Yes	Multiple times daily	RTC
38.6717	-87.5128	anonymous	EN	No							

41.6524	-85.0143	anonymous	EN	Yes	71	Female	$30,000 - $59,999	At home on your phone/computer etc.	Yes	Multiple times daily	Frontier Communications

38.6624	-86.916	anonymous	EN	Yes	68	Female	$90,000 - $119,999	At home on your phone/computer etc.	Yes	Once a day	RTC
41.2291	-85.3295	anonymous	EN	Yes	67	Female	$90,000 - $119,999	Public setting on your phone/computer etc.	Yes	Multiple times daily	RTC

38.6624	-86.916	anonymou s	EN	Yes	67	Female	$60,000 - $89,999	At home on your phone/co mputer etc.	Yes	Multiple times daily	RTC

38.6624	-86.916	anonymous	EN	Yes	80	Female	$30,000 - $59,999	At home on your phone/computer etc.	Yes	Multiple times daily	RTC

39.2318	-85.8884	anonymous	EN	Yes	52	Female	greater than $120,000	At home on your phone/computer etc.	Yes	Multiple times daily	RTC

38.0461	-87.2579	anonymous	EN	Yes	64	Female	$90,000 - $119,999	Public setting on your phone/computer etc.	Yes	Multiple times daily	Frontier Communications

41.2291	-85.3295	anonymous	EN	Yes	73	Male	$60,000 - $89,999	At home on your phone/computer etc.	Yes	Multiple times a week	Frontier Communications
38.2539	-85.76	anonymous	EN	Yes	54	Female	$90,000 - $119,999	At home on your phone/computer etc.	Yes	Multiple times daily	Hughes Net

38.6624	-86.916	anonymous	EN	Yes	65	Female	$60,000 - $89,999	At home on your phone/computer etc.	Yes	Multiple times daily	Frontier Communications

38.6624	-86.916	anonymous	EN	Yes	49	Male	$30,000 - $59,999	At home on your phone/computer etc.	Yes	Multiple times daily	RTC

38.6752	-86.7747	anonymous	EN	Yes	51	Female	$60,000 - $89,999	At home on your phone/computer etc.	Yes	Multiple times daily	RTC

38.7735	-86.6285	anonymous	EN	Yes	64	Female	$30,000 - $59,999	At home on your phone/computer etc.	Yes	Multiple times daily	RTC

38.6624	-86.916	anonymous	EN	Yes	55	Female	greater than $120,000	At home on your phone/computer etc.	Yes	Multiple times daily	Other (please specify)	Sparklight

39.6598	-87.4225	anonymous	EN	Yes	46	Female	less than $30,000	At home on your phone/computer etc.	Yes	Multiple times daily	Viasat
39.2051	-85.9324	anonymous	EN	Yes	48	Female	$90,000 - $119,999	At home on your phone/computer etc.	Yes	Multiple times daily	T-Mobile

39.2051	-85.9324	anonymous	EN	Yes	20	Female	$30,000 - $59,999	At home on your phone/computer etc.	Yes	Multiple times daily	Frontier Communications

39.1515	-85.1305	anonymous	EN	Yes	71	Female	$90,000 - $119,999	Public setting on your phone/computer etc.	Yes	Multiple times daily	Frontier Communications
39.2318	-85.8884	anonymous	EN	Yes	23	Female	$30,000 - $59,999	At home on your phone/computer etc.	Yes	Once a day	Cell phone company

38.6624	-86.916	anonymous	EN	Yes	25	Female	$30,000 - $59,999	At home on your phone/computer etc.	Yes	Multiple times daily	RTC

38.7735	-86.6285	anonymous	EN	Yes	47	Female	$90,000 - $119,999	At home on your phone/computer etc.	Yes	Multiple times daily	RTC

41.8874	-87.6318	anonymous	EN	Yes	64	Female	$60,000 - $89,999	At home on your phone/computer etc.	Yes	Multiple times daily	Other (please specify)	Starlink

41.8719	-87.6589	anonymous	EN	Yes	59	Female	$90,000 - $119,999	At home on your phone/computer etc.	Yes	Multiple times daily	Other (please specify)

38.5848	-90.2996	anonymous	EN	Yes	52	Male	$60,000 - $89,999	At home on your phone/computer etc.	Yes	Multiple times daily	Viasat

41.8764	-87.6133	anonymous	EN	Yes	56	Male	$30,000 - $59,999	At home on your phone/computer etc.	Yes	Multiple times daily	Cell phone company
34.627	-79.6893	anonymous	EN	Yes	70	Female	$60,000 - $89,999	At home on your phone/computer etc.	Yes	Multiple times daily	Viasat

38.061	-87.5501	anonymous	EN	Yes	56		Female	less than $30,000	At home on your phone/computer etc.	Yes	Multiple times daily	Other (please specify)	Icg wireless

39.2051	-85.9324	anonymous	EN	Yes	32	Female	$90,000 - $119,999	At home on your phone/computer etc.	Yes	Multiple times daily	Other (please specify)	Bridgemaxx

41.4379	-85.021	anonymous	EN	Yes	64	Male	$30,000 - $59,999	At home on your phone/computer etc.	Yes	Multiple times daily	Frontier Communications

38.6624	-86.916	anonymous	EN	Yes	42	Female	less than $30,000	At home on your phone/computer etc.	Yes	Multiple times daily	RTC

38.6624	-86.916	anonymous	EN	Yes	34	Female	greater than $120,000	At home on your phone/computer etc.	Yes	Multiple times daily	RTC

41.3458	-85.4155	anonymous	EN	Yes	55	Female	$90,000 - $119,999	At home on your phone/computer etc.	Yes	Multiple times daily	Frontier Communications

38.6529	-87.1699	anonymous	EN	Yes	23	Male	$30,000 - $59,999	At home on your phone/computer etc.	Yes	Multiple times daily	Other (please specify)	Sparklight
39.2051	-85.9324	anonymous	EN	Yes	64	Female	$60,000 - $89,999	At home on your phone/computer etc.	Yes	Multiple times daily	T-Mobile	
39.4732	-87.4033	anonymous	EN	Yes		Female	$90,000 - $119,999	At home on your phone/computer etc.	Yes	Multiple times daily	Frontier Communications	

39.4956	-87.4651	anonymous	EN	Yes	48	Female	$90,000 - $119,999	Public setting on your phone/computer etc.	Yes	Multiple times daily	Frontier Communications	
38.6529	-87.1699	anonymous	EN	Yes	54	Female	$90,000 - $119,999	At home on your phone/computer etc.	Yes	Multiple times daily	Other (please specify)	Sparklight

38.6624	-86.916	anonymous	EN	Yes	65	Female	less than $30,000	At home on your phone/computer etc.	Yes	Multiple times daily	RTC
		anonymous	EN	Yes							
		anonymous	EN	Yes	67	Female	$60,000 - $89,999	At home on your phone/computer etc.	Yes	Multiple times daily	Frontier Communications

anonymous	EN	Yes	54	Female	less than $30,000	At home on your phone/computer etc.	Yes	Multiple times daily	Other (please specify)	Sparklight
anonymous	EN	Yes	45	Female	greater than $120,000	At home on your phone/computer etc.	Yes			
anonymous	EN	Yes	68	Male	$30,000 - $59,999	Public setting on your phone/computer etc.	Yes	Multiple times daily	Other (please specify)	Verizon
anonymous	EN	Yes	42	Female	$30,000 - $59,999	At home on your phone/computer etc.	Yes	Multiple times daily	Cell phone company	
anonymous	EN	Yes	24	Female	$30,000 - $59,999	At home on your phone/computer etc.	Yes			
anonymous	EN									

		anonymous	EN	Yes	31	Female	$60,000 - $89,999	At home on your phone/computer etc.			
		anonymous	EN								
		anonymous	EN	Yes	30	Male	$60,000 - $89,999	At home on your phone/computer etc.	Yes	Multiple times daily	RTC
38.6624	-86.916	anonymous	EN	Yes	71	Female	less than $30,000	At home on your phone/computer etc.	Yes	Multiple times daily	RTC

anonymous	EN	Yes	19	Female	$90,000 - $119,999	Public setting on your phone/computer etc.	Yes	Multiple times daily	Other (please specify)	Wagler Internet
anonymous	EN	Yes	26	Male	$60,000 - $89,999	At home on your phone/computer etc.	Yes	Multiple times daily	RTC	
anonymous	EN	Yes	26	Female	$30,000 - $59,999	At home on your phone/computer etc.	Yes	Multiple times daily	Other (please specify)	Spark light
anonymous	EN	Yes	56	Female		At home on your phone/computer etc.	Yes	Multiple times daily	Cell phone company	

		anonymous	EN	Yes	39	Female	$30,000 - $59,999	At home on your phone/computer etc.	Yes		
39.4057	-87.4026	anonymous	EN	Yes	53	Female	$30,000 - $59,999	At home on your phone/computer etc.	Yes	Multiple times daily	Frontier Communications
		anonymous	EN	Yes	72	Female	less than $30,000	At home on your phone/computer etc.	Yes		
		anonymous	EN								
		anonymous	EN	Yes	30	Female	$30,000 - $59,999	Public setting on your phone/computer etc.	Yes		

38.6529	-87.1699	anonymous	EN	Yes	58	Female	$30,000 - $59,999	At home on your phone/computer etc.	Yes	Multiple times daily	Other (please specify)	Sparklight

38.7735	-86.6285	anonymous	EN	Yes	46	Female	greater than $120,000	At home on your phone/computer etc.	Yes	Multiple times daily	RTC
		anonymous	EN								

38.6624	-86.916	anonymous	EN	Yes	59	Female	$60,000 - $89,999	At home on your phone/computer etc.	Yes	Multiple times daily	RTC
		anonymous	EN	Yes							

38.6624	-86.916	anonymous	EN	Yes	64	Female	$60,000 - $89,999	At home on your phone/computer etc.	Yes	Multiple times daily	RTC
		anonymous	EN	Yes	25	Female	$90,000 - $119,999	Public setting on your phone/computer etc.	Yes		
		anonymous	EN	Yes	45	Female	$30,000 - $59,999	Public setting on your phone/computer etc.	Yes		

38.6624	-86.916	anonymous	EN	Yes	59	Male	$90,000 - $119,999	At home on your phone/computer etc.	Yes	Multiple times daily	RTC	
		anonymous	EN									
39.2947	-85.2292	qr	EN	No								
38.3317	-87.3562	anonymous	EN	Yes	68	Female	less than $30,000	At home on your phone/computer etc.	Yes	Multiple times daily	Other (please specify)	Sparklight

anonymous	EN								
anonymous	EN	Yes	25	Female	$30,000 - $59,999	Public setting on your phone/computer etc.	Yes		
anonymous	EN	Yes	38	Female	$60,000 - $89,999	At home on your phone/computer etc.	Yes	Multiple times daily	Frontier Communications
anonymous	EN	Yes	76	Female	$30,000 - $59,999	At home on your phone/computer etc.	Yes	Multiple times daily	Hughes Net

38.8729	-86.486	anonymous	EN	Yes	60	Female	less than $30,000	At home on your phone/computer etc.	Yes	Multiple times daily	Cell phone company
		anonymous	EN	Yes	37	Female	$60,000 - $89,999	At home on your phone/computer etc.	Yes		

38.538	-86.6121	anonymous	EN	Yes	47	Female	greater than $120,000	At home on your phone/computer etc.	Yes	Multiple times daily	Smithville

39.1397	-86.5141	anonymous	EN	Yes	46	Female	greater than $120,000	Public setting on your phone/computer etc.	Yes	Multiple times daily	Space Exploration Technologies

38.745	-86.4803	anonymous	EN	Yes	42	Female	$60,000 - $89,999	At home on your phone/computer etc.	Yes	Multiple times daily	Other (please specify)	Frontier Communications and Cell phone Data

40.7157	-74	anonymous	EN	Yes	51	Female	$90,000 - $119,999	Public setting on your phone/computer etc.	Yes	Multiple times daily	Smithville

39.6159	-85.43	qr	EN	Yes	48	Female	$60,000 - $89,999	At home on your phone/computer etc.	Yes	Once a day	Other (please specify)	Verizon

39.6195	-86.1552	anonymous	EN	Yes	28		Female	$90,000 - $119,999	Public setting on your phone/computer etc.	Yes	Multiple times daily	Other (please specify)	Sparklight

38.4792	-87.2834	anonymous	EN	Yes	82	Male	$60,000 - $89,999	At home on your phone/computer etc.	Yes	Multiple times daily	Cable One

38.4792	-87.2834	anonymous	EN	Yes	36	Female	$30,000 - $59,999	At home on your phone/computer etc.	Yes	Multiple times daily	RTC
		anonymous	EN	Yes	37	Female	$90,000 - $119,999	At home on your phone/computer etc.	Yes	Multiple times daily	RTC

39.4642	-87.3531	anonymous	EN	Yes	26	Male	$60,000 - $89,999	Public setting on paper	Yes	Multiple times daily	Other (please specify)	starlink
39.4642	-87.3531	anonymous	EN	Yes	58	Female	less than $30,000	Public setting on paper	Yes	Multiple times daily	Cell phone company	

39.4642	-87.3531	anonymous	EN	Yes	40	Female	greater than $120,000	Public setting on paper	Yes	Multiple times daily	RTC

39.4642	-87.3531	anonymous	EN	Yes	44	Female	$90,000 - $119,999	Public setting on paper	Yes	Multiple times daily	Frontier Communications

39.4642	-87.3531	anonymous	EN	Yes	62	Male	less than $30,000	Public setting on paper	Yes	Multiple times daily	Cell phone company

39.4642	-87.3531	anonymous	EN	Yes	50	Male	$30,000 - $59,999	Public setting on paper	Yes	Multiple times daily	T-Mobile

39.4642	-87.3531	anonymous	EN	Yes	70	Male	$60,000 - $89,999	Public setting on paper	Yes	Multiple times daily	Frontier Communications

39.4642	-87.3531	anonymous	EN	Yes	72	Female	greater than $120,000	Public setting on paper	Yes	Multiple times daily	RTC
39.4642	-87.3531	anonymous	EN	Yes	44	Male	$60,000 - $89,999	Public setting on paper	No		

39.4642	-87.3531	anonymous	EN	Yes	62	Female	less than $30,000	Public setting on paper	Yes	Multiple times daily	T-Mobile		
39.4642	-87.3531	anonymous	EN	Yes	68	Female	less than $30,000	Public setting on paper	Yes	Multiple times daily	Viasat		
39.4642	-87.3531	anonymous	EN	Yes	63	Male	less than $30,000	At home on paper	Yes	Multiple times daily	Other (please specify)	AT&T	

39.4642	-87.3531	anonymous	EN	Yes	90	Female	less than $30,000	Public setting on paper	No		
39.4642	-87.3531	anonymous	EN	Yes	47	Female	$60,000 - $89,999	Public setting on paper	Yes	Multiple times daily	Cell phone company
39.4642	-87.3531	anonymous	EN	Yes	22	Female	$60,000 - $89,999	Public setting on paper	Yes	Multiple times daily	Frontier Communications

39.4642	-87.3531	anonymous	EN	Yes	25	Male	greater than $120,000	Public setting on paper	Yes	Multiple times daily	Other (please specify)	EarthLink

39.4642	-87.3531	anonymou s	EN	Yes	56	Male	$30,000 - $59,999	Public setting on paper	No

39.4642	-87.3531	anonymous	EN	Yes	68	Female	less than $30,000	Public setting on paper	No
		anonymous	EN						

Q11	Q11_7_TEX	Q36	Q32	Q33	Q34	Q35	Q35_8_TEX	Q16	Q15	Q18	Q35	Q35_8_TEX
What type (	What type (	When did y	Before Mar	How many	What type (	What appo	What appo	During the	From Marcl	What type (	What appo	What appo
Broadband (DSL or Cable)		Over two years ago	No					No				

Fiber Optic		Within the last two years	No						Yes	1-5	Video appointment from home	Behavioral/Mental health therapy		

Cell phone data only		Within the last month	Yes	1-5	Video from physician's office to another provider	Surgery follow-up		Yes	6-10	Video from physician's office to another provider	Surgery follow-up

Fiber Optic		Within the last year	No					Yes	1-5	Telephone appointment	Specialty care (cardiologist, pulmonologist, nephrologist, etc)

Satellite	Over two years ago	No		Yes	1-5	Video from physician's office to another provider,Telephone appointment	Behavioral/Mental health therapy

Broadband (DSL or Cable)		Over two years ago	No	Yes	1-5	Video appointment from home,Telephone appointment	Primary Care visit (family medicine, internal medicine, etc.),Appointment to discuss and adjust medications only

Cellular modem			Within the last two years	Yes	1-5	Video appointment from home	Specialty care (cardiologist, pulmonologist, nephrologist, etc)		Yes	1-5	Video appointment from home	Other (please specify)	For disability

Unsure	Over two years ago	No	No

Other (please specify)	Hot spot	Within the last two years	No	No

Fiber Optic	Over two years ago	No	No

Broadband (DSL or Cable)		Over two years ago	No					Yes	6-10	Video appointment from home,Telephone appointment	Primary Care visit (family medicine, internal medicine, etc.),Appointment to discuss and adjust medications only

Unsure	Over two years ago	No	No

Broadband (DSL or Cable)	Over two years ago	No	No

Broadband (DSL or Cable)	Over two years ago	No	No

Satellite	Over two years ago	No	No

Unsure	Over two years ago	No					No				
Cellular modem	Within the last year	No						1-5		Other (please specify)	None

Cell phone data only	Over two years ago	No	No
Fiber Optic	Over two years ago	No	No

Cellular modem		Over two years ago	No					Yes	16-20	Video appointment from home,Telephone appointment	Behavioral/Mental health therapy,Primary Care visit (family medicine, internal medicine, etc.),Appointment to discuss and adjust medications only

Broadband (DSL or Cable)		Over two years ago	No					Yes	21+	Video appointment from home,Telephone appointment	Behavioral/Mental health therapy,Primary Care visit (family medicine, internal medicine, etc.)

Fiber Optic		Over two years ago	No					No			
Fiber Optic		Within the last year	No					Yes	1-5	Video appointment from home	Appointment to discuss and adjust medications only

Broadband (DSL or Cable)		Within the last two years	No					Yes	1-5	Video appointment from home	Specialty care (cardiologist, pulmonologist, nephrologist, etc)

Fiber Optic	Within the last two years	No		Yes	1-5	Video appointment from home	Primary Care visit (family medicine, internal medicine, etc.)

Broadband (DSL or Cable)		Over two years ago	No					Yes	1-5	Telephone appointment	Specialty care (cardiologist, pulmonologist, nephrologist, etc)

Unsure		Over two years ago	Yes		Other (please specify)	None	Yes	6-10	Video appointment from home,Telephone appointment	Primary Care visit (family medicine, internal medicine, etc.),Specialty care (cardiologist, pulmonologist, nephrologist, etc)

Fiber Optic	Over two years ago	No	No

Fiber Optic		Within the last year	No					No				
Fiber Optic		Within the last year	No					Yes	1-5	Telephone appointment	Other (please specify)	Urgent care type visit

Fiber Optic		Over two years ago	No					Yes	1-5	Video appointment from home	Specialty care (cardiologist, pulmonologist, nephrologist, etc)

Satellite	Within the last month	No		Yes	1-5	Video appointment from home	Primary Care visit (family medicine, internal medicine, etc.)

Fiber Optic	Within the last year	No	No
Fiber Optic	Within the last month	No	No
Satellite	Within the last two years	No	No

Fiber Optic		Within the last two years	No					Yes	1-5	Video appointment from home	Other (please specify)	Dermatologist

Fiber Optic	Within the last year	No	No

Broadband (DSL or Cable)		Within the last 6 months	No					Yes	1-5	Video appointment from home	Primary Care visit (family medicine, internal medicine, etc.)
Fiber Optic		Over two years ago	No					No			

Cellular modem	Within the last year	No		Yes	1-5	Video appointment from home,Telephone appointment	Behavioral/Mental health therapy,Surgery follow-up,Specialty care (cardiologist, pulmonologist, nephrologist, etc)

Broadband (DSL or Cable)	Over two years ago	No	No

Unsure	Within the last 6 months	No	No

Broadband (DSL or Cable)	Over two years ago	No	No

Fiber Optic		Within the last two years	No					Yes	1-5	Telephone appointment	Primary Care visit (family medicine, internal medicine, etc.)

Fiber Optic		Within the last two years	No					Yes	1-5	Telephone appointment	Primary Care visit (family medicine, internal medicine, etc.)

Unsure	Within the last year	No	No
Fiber Optic	Within the last year	No	No

Fiber Optic	Within the last two years	No	No

Unsure	Over two years ago	No	No

Fiber Optic		Within the last year	No					Yes	1-5	Video appointment from home	Specialty care (cardiologist, pulmonologist, nephrologist, etc)
Cellular modem		Over two years ago	No					No			

Broadband (DSL or Cable)	Over two years ago	No		Yes	1-5	Video appointment from home	Primary Care visit (family medicine, internal medicine, etc.)

Fiber Optic	Within the last year	No	Yes	1-5	Telephone appointment	Specialty care (cardiologist, pulmonologist, nephrologist, etc)

Fiber Optic		Within the last year	No					No			
Cell phone data only		Over two years ago	No					Yes	1-5	Video appointment from home	Specialty care (cardiologist, pulmonologist, nephrologist, etc)

Broadband (DSL or Cable)		Within the last two years	No						Yes	1-5	Video appointment from home	Specialty care (cardiologist, pulmonologist, nephrologist, etc),Appointment to discuss and adjust medications only		

Cellular modem		Within the last two years	No					Yes	1-5	Video appointment from home	Specialty care (cardiologist, pulmonologist, nephrologist, etc)

Fiber Optic	Within the last two years	No		Yes	1-5	Video appointment from home	Primary Care visit (family medicine, internal medicine, etc.)

Unsure	Within the last month	No		Yes	1-5	Telephone appointment	Behavioral/Mental health therapy,Primary Care visit (family medicine, internal medicine, etc.),Appointment to discuss and adjust medications only

Unsure	Over two years ago	No	No

Fiber Optic	Within the last year	No		6-10	Video appointment from home,Telephone appointment	Primary Care visit (family medicine, internal medicine, etc.),Specialty care (cardiologist, pulmonologist, nephrologist, etc),Appointment to discuss and adjust medications only

Unsure	Within the last year	No	No

Fiber Optic		Over two years ago	No				Yes	1-5	Video appointment from home,Telephone appointment	Behavioral/Mental health therapy,Primary Care visit (family medicine, internal medicine, etc.),Appointment to discuss and adjust medications only

Fiber Optic		Within the last two years	No					Yes	1-5	Video appointment from home	Specialty care (cardiologist, pulmonologist, nephrologist, etc)
Broadband (DSL or Cable)		Over two years ago	No					No			

Broadband (DSL or Cable)		Within the last two years	No					Yes	1-5	Telephone appointment	Primary Care visit (family medicine, internal medicine, etc.)	

Fiber Optic	Within the last two years	No	No

Unsure	Within the last two years	No	No

Unsure	Within the last year	No	No
Broadband (DSL or Cable)	Over two years ago	No	No

Broadband (DSL or Cable)	Within the last year	No		1-5
		No	No	

Broadband (DSL or Cable)		Over two years ago	No					Yes	1-5	Video appointment from home	Specialty care (cardiologist, pulmonologist, nephrologist, etc)
Unsure		Over two years ago	No						1-5	Telephone appointment	Appointment to discuss and adjust medications only
Fiber Optic		Within the last two years	No					No			

Fiber Optic		Over two years ago	No					Yes	6-10	Video appointment from home	Primary Care visit (family medicine, internal medicine, etc.)

Cellular modem		Within the last month	No					Yes	1-5	Telephone appointment	Behavioral /Mental health therapy,Primary Care visit (family medicine, internal medicine, etc.)
Fiber Optic		Within the last year	No					No			

Fiber Optic	Within the last year	No	Yes	1-5	Video appointment from home,Video from physician's office to another provider	Surgery follow-up,Specialty care (cardiologist, pulmonologist, nephrologist, etc)
Cell phone data only	Within the last year	No	No			
Broadband (DSL or Cable)	Within the last 6 months	No	No			

Cellular modem	Over two years ago	No	No

Broadband (DSL or Cable)	Within the last year	No		Yes	1-5	Video appointment from home	Appointment to discuss and adjust medications only

Fiber Optic	Within the last two years	No		Yes	1-5	Video appointment from home,Video from physician's office to another provider	Specialty care (cardiologist, pulmonologist, nephrologist, etc)

Unsure	Over two years ago	No		Yes	1-5	Video appointment from home,Telephone appointment	Primary Care visit (family medicine, internal medicine, etc.)
Cell phone data only	Over two years ago	No		No			

Broadband (DSL or Cable)	Over two years ago	No	No
Fiber Optic	Within the last two years	No	No

Fiber Optic		Within the last year	No					Yes	1-5	Video appointment from home	Specialty care (cardiologist, pulmonologist, nephrologist, etc)

Broadband (DSL or Cable)		Over two years ago	No					Yes	6-10	Video appointment from home,Telephone appointment	Behavioral /Mental health therapy
Fiber Optic		Within the last year	No					No			

Fiber Optic	Within the last two years	No	No

Fiber Optic		Within the last two years	Yes	1-5	Video appointment from home	Primary Care visit (family medicine, internal medicine, etc.),Specialty care (cardiologist, pulmonologist, nephrologist, etc),Appointment to discuss and adjust medications only		Yes	1-5	Video appointment from home	Specialty care (cardiologist, pulmonologist, nephrologist, etc),Appointment to discuss and adjust medications only

Satellite	Over two years ago	No	No
Fiber Optic	Over two years ago	No	No

No

No

Fiber Optic		Within the last 6 months	No					Yes	1-5	Video appointment from home	Primary Care visit (family medicine, internal medicine, etc.)
Broadband (DSL or Cable)		Within the last 6 months	No					No			

Fiber Optic		Within the last year	No					Yes	1-5	Telephone appointment	Specialty care (cardiologist, pulmonologist, nephrologist, etc),Appointment to discuss and adjust medications only

Fiber Optic	Within the last year	No		Yes	1-5	Video appointment from home,Telephone appointment	Primary Care visit (family medicine, internal medicine, etc.),Specialty care (cardiologist, pulmonologist, nephrologist, etc)

Fiber Optic	Over two years ago	No		No

No

No

Fiber Optic	Within the last year	No	No

Fiber Optic	Within the last year	No	No
Fiber Optic	Within the last year	No	No

Unsure		Over two years ago	No					Yes	1-5	Video appointment from home,Telephone appointment	Primary Care visit (family medicine, internal medicine, etc.),Specialty care (cardiologist, pulmonologist, nephrologist, etc),Appointment to discuss and adjust medications only

Broadband (DSL or Cable)		Over two years ago	No					Yes	1-5	Video appointment from home	Primary Care visit (family medicine, internal medicine, etc.)

Fiber Optic	Within the last year	No	No

Fiber Optic	Within the last year	No	No

Fiber Optic		Within the last year	No			Yes	1-5	Video appointment from home	Specialty care (cardiologist, pulmonologist, nephrologist, etc)

Cell phone data only		Over two years ago	No		Yes	1-5	Video from physician's office to another provider	Specialty care (cardiologist, pulmonologist, nephrologist, etc)

Cell phone data only	Over two years ago	No	No
Cell phone data only	Within the last year	No	No

Fiber Optic		Within the last year	Yes	6-10	Video appointment from home	Primary Care visit (family medicine, internal medicine, etc.)		Yes	6-10	Video appointment from home	Primary Care visit (family medicine, internal medicine, etc.)

Satellite	Over two years ago	No		Yes	1-5	Video from physician's office to another provider,Telephone appointment	Primary Care visit (family medicine, internal medicine, etc.),Other (please specify)	MFM-Obstetrics

Broadband (DSL or Cable)		Within the last two years	Yes	1-5	Video appointment from home,Telephone appointment	Primary Care visit (family medicine, internal medicine, etc.)		Yes	1-5	Telephone appointment	Primary Care visit (family medicine, internal medicine, etc.)

Fiber Optic	Within the last two years	No		1-5
Unsure	Within the last 6 months	No	No	

Broadband (DSL or Cable)		Over two years ago	No					No			
Fiber Optic		Over two years ago	No					Yes	6-10	Video appointment from home,Telephone appointment	Behavioral/Mental health therapy,Primary Care visit (family medicine, internal medicine, etc.)

Fiber Optic		Within the last two years	No					Yes	1-5	Video appointment from home	Primary Care visit (family medicine, internal medicine, etc.),Appointment to discuss and adjust medications only
Unsure		Over two years ago	No					No			

Fiber Optic		Over two years ago	Yes	1-5	Telephone appointment	Surgery follow-up		Yes	1-5	Video appointment from home,Telephone appointment	Surgery follow-up,Specialty care (cardiologist, pulmonologist, nephrologist, etc)
Unsure		Within the last year	No					No			

Cell phone data only		Within the last 6 months	Yes	1-5	Video appointment from home	Behavioral /Mental health therapy		Yes	11-15	Video appointment from home	Behavioral /Mental health therapy

Fiber Optic	Within the last year	No	No

Unsure		Within the last month	No				Yes	6-10	Video appointment from home	Behavioral/Mental health therapy,Appointment to discuss and adjust medications only

Cell phone data only		Within the last two years	No					Yes	1-5	Telephone appointment	Primary Care visit (family medicine, internal medicine, etc.)

Fiber Optic		Within the last year	Yes	1-5	Video appointment from home	Primary Care visit (family medicine, internal medicine, etc.)		Yes	1-5	Video appointment from home	Primary Care visit (family medicine, internal medicine, etc.)
Broadband (DSL or Cable)		Within the last 6 months	No					No			

Broadband (DSL or Cable)	Within the last two years	No	No
Fiber Optic	Over two years ago	No	No

Unsure	Over two years ago	No	No

Cellular modem	Within the last 6 months	No	No

Unsure		Within the last two years	No						Yes	1-5	Video appointment from home	Behavioral/Mental health therapy,Primary Care visit (family medicine, internal medicine, etc.)			

Cell phone data only	Over two years ago	No	No
Cell phone data only	Over two years ago	No	No

Broadband (DSL or Cable)		Within the last year	No					Yes	1-5	Video appointment from home	Primary Care visit (family medicine, internal medicine, etc.)	

Fiber Optic		Within the last two years	No					No			
Cell phone data only		Over two years ago	No					Yes	1-5	Telephone appointment	Surgery follow-up

Broadband (DSL or Cable)	Over two years ago	No	No
Unsure	Over two years ago	No	No

Fiber Optic		Within the last year	No					No			
Fiber Optic		Within the last year	No					Yes	1-5	Video appointment from home	Specialty care (cardiologist, pulmonologist, nephrologist, etc)

Cell phone data only	Within the last year	No			No
Broadband (DSL or Cable)	Over two years ago	No			No

Fiber Optic		Within the last two years	No		Yes	1-5	Video appointment from home	Primary Care visit (family medicine, internal medicine, etc.)

Fiber Optic	Within the last two years	No		1-5	Telephone appointment	Specialty care (cardiologist, pulmonologist, nephrologist, etc)
Fiber Optic	Over two years ago	No	No			

Fiber Optic	Within the last two years	No		Yes	16-20	Video appointment from home,Video from physician's office to another provider,Telephone appointment	Primary Care visit (family medicine, internal medicine, medicine, etc.)

Unsure	Within the last year	No	No

Cellular modem	Over two years ago	No	No

Cellular modem		Within the last year	No	No
Other (please specify)	Hot spot	Over two years ago	No	No

Fiber Optic		Within the last 6 months	No					Yes	11-15	Video appointment from home	Other (please specify)	Functional medicine
Fiber Optic		Within the last two years	No					No				

Satellite	Over two years ago	No	No

Fiber Optic	Over two years ago	No	No

Broadband (DSL or Cable)	Over two years ago	No	No

Fiber Optic	Over two years ago	No	Yes	1-5	Telephone appointment	Specialty care (cardiologist, pulmonologist, nephrologist, etc)
Fiber Optic	Over two years ago	No	No			

Fiber Optic	Within the last year	No	No

Fiber Optic	Within the last 6 months	No	No

Fiber Optic		Within the last year	No					Yes	6-10	Video appointment from home,Telephone appointment	Primary Care visit (family medicine, internal medicine, etc.),Specialty care (cardiologist, pulmonologist, nephrologist, etc),Appointment to discuss and adjust medications only

Broadband (DSL or Cable)	Over two years ago	No	No

Broadband (DSL or Cable)	Over two years ago	No	No
Satellite	Within the last year	No	No

Unsure	Over two years ago	No	No

Fiber Optic	Within the last year	No	No

Fiber Optic	Within the last two years	No	No

Fiber Optic	Within the last two years	No		No

Cellular modem		Over two years ago	No		Yes	6-10	Video appointment from home	Primary Care visit (family medicine, internal medicine, etc.),Other (please specify)	IU HEALTH EMPLOYEE VIRTUAL VISIT FOR COVID SCREENS/ RTW

Satellite		Within the last 6 months	No					Yes	6-10	Video appointment from home,Telephone appointment	Behavioral/Mental health therapy,Primary Care visit (family medicine, internal medicine, etc.),Appointment to discuss and adjust medications only
Unsure		Within the last two years	No					Yes	1-5	Video appointment from home	Specialty care (cardiologist, pulmonologist, nephrologist, etc)

Unsure	Over two years ago	No		Yes	1-5	Video appointment from home,Telephone appointment	Surgery follow-up,Specialty care (cardiologist, pulmonologist, nephrologist, etc),Appointment to discuss and adjust medications only

Broadband (DSL or Cable)		Over two years ago	No						1-5	Telephone appointment	Primary Care visit (family medicine, internal medicine, etc.)
Cell phone data only		Over two years ago	No					No			

Fiber Optic		Within the last year	No					Yes	1-5	Video from physician's office to another provider	Behavioral /Mental health therapy

Fiber Optic		Within the last two years	No					Yes	6-10	Video appointment from home	Specialty care (cardiologist, pulmonologist, nephrologist, etc)

Satellite	Within the last two years	No	No

Satellite	Within the last two years	No		No

Satellite		Over two years ago	No					Yes	1-5	Video appointment from home	Other (please specify)	Dermatology

Cell phone data only		Over two years ago	Yes	1-5	Telephone appointm ent	Primary Care visit (family medicine, internal medicine, etc.)		Yes	1-5	Telephone appointm ent	Primary Care visit (family medicine, internal medicine, etc.)
Satellite		Within the last two years	No					No			

Cellular modem		Within the last 6 months	No					Yes	21+	Video appointment from home	Behavioral/Mental health therapy,Appointment to discuss and adjust medications only

Broadband (DSL or Cable)	Within the last two years	No	No

Broadband (DSL or Cable)		Over two years ago	No					Yes	1-5	Video appointment from home,Telephone appointment	Primary Care visit (family medicine, internal medicine, etc.)

Fiber Optic	Within the last 6 months	No	No

Fiber Optic		Within the last year	No					Yes	1-5	Video appointment from home	Behavioral /Mental health therapy

Unsure		Within the last two years	Yes	1-5	Telephone appointment	Behavioral/Mental health therapy		Yes	1-5	Video appointment from home	Behavioral/Mental health therapy

Fiber Optic	Over two years ago	No	No
Cellular modem	Within the last month	No	No
Broadband (DSL or Cable)	Within the last year	No	No

Unsure	Within the last two years	No	No
Cellular modem	Over two years ago	No	No

Fiber Optic		Within the last year	No					Yes	1-5	Video appointment from home	Primary Care visit (family medicine, internal medicine, etc.)
Unsure		Over two years ago	No					No			

Unsure	Over two years ago	No	No
Unsure	Within the last two years	No	
Cell phone data only	Within the last year	No	No

Fiber Optic		Within the last 6 months	No					Yes	1-5	Video appointment from home	Primary Care visit (family medicine, internal medicine, etc.),Appointment to discuss and adjust medications only
Other (please specify)		Within the last year	No					No			

Fiber Optic		Within the last two years	No					No			
Fiber Optic		Within the last year	No					Yes	1-5	Telephone appointment	Behavioral/Mental health therapy,Appointment to discuss and adjust medications only
Other (please specify)	Wi-Fi and Cellphone	Within the last two years	No								
Cell phone data only		Over two years ago	Yes	1-5	Telephone appointment	Other (please specify)	Sick	Yes	1-5	Telephone appointment	Primary Care visit (family medicine, internal medicine, etc.)

Fiber Optic	Within the last year	No	No

Broadband (DSL or Cable)	Over two years ago	No	No

Fiber Optic	Over two years ago	No	No

Fiber Optic	Within the last year	No	No

Fiber Optic	Within the last 6 months	No	No

Fiber Optic		Within the last year	No					No			
Broadband (DSL or Cable)		Over two years ago	No					Yes	1-5	Telephone appointment	Primary Care visit (family medicine, internal medicine, etc.)

Broadband (DSL or Cable)	Within the last two years		
Satellite	Within the last two years	No	No

Cell phone data only	Over two years ago	No				No

Unsure		Over two years ago	No		Yes	1-5	Video appointment from home,Telephone appointment	Primary Care visit (family medicine, internal medicine, etc.),Specialty care (cardiologist, pulmonologist, nephrologist, etc)

Satellite	Within the last two years	No	No

Other (please specify)	Broadband and cell phone data	Over two years ago	No					Yes	6-10	Video appointment from home,Telephone appointment	Specialty care (cardiologist, pulmonologist, nephrologist, etc),Other (please specify)	Developmental evaluation

Broadband (DSL or Cable)	Over two years ago	No		No

Other (please specify)	WiFi jetpak through Verizon	Within the last year	No					Yes	6-10	Video appointment from home,Telephone appointment	Behavioral/Mental health therapy,Other (please specify)	Spine & Pain, Rheumatology

Fiber Optic	Within the last year	No	No

Broadband (DSL or Cable)	Over two years ago	No	No

Fiber Optic		Within the last 6 months	No					Yes	1-5	Video appointment from home	Specialty care (cardiologist, pulmonologist, nephrologist, etc),Appointment to discuss and adjust medications only
Fiber Optic		Within the last year	No					No			

Satellite	Within the last month	No			Yes	1-5	Telephone appointment	Primary Care visit (family medicine, internal medicine, etc.)
Cell phone data only	Within the last month	No			Yes	1-5	Telephone appointment	Specialty care (cardiologist, pulmonologist, nephrologist, etc)

Fiber Optic	Within the last two years	No	Yes	1-5	Video appointment from home	Primary Care visit (family medicine, internal medicine, etc.)

Broadband (DSL or Cable)		Over two years ago	No					Yes	1-5	Video appointment from home	Specialty care (cardiologist, pulmonologist, nephrologist, etc),Appointment to discuss and adjust medications only

Cell phone data only	Over two years ago	No		Yes	1-5	Video appointment from home	Specialty care (cardiologist, pulmonologist, nephrologist, etc)

Cellular modem	Within the last year	No	No

Broadband (DSL or Cable)	Over two years ago	No	No

Fiber Optic	Within the last two years	No	No
		No	No

Unsure	Within the last month	No	No			
Satellite	Within the last 6 months	No	Yes	6-10	Video appointment from home,Telephone appointment	Specialty care (cardiologist, pulmonologist, nephrologist, etc)
Cell phone data only	Over two years ago	No	No			

			No					No			
Broadband (DSL or Cable)		Over two years ago	No					No			
Broadband (DSL or Cable)		Within the last year	No					Yes	1-5	Telephone appointment	Primary Care visit (family medicine, internal medicine, etc.)

Broadband (DSL or Cable)	Within the last year	No	No

No		Yes	1-5	Video appointment from home,Telephone appointment	Primary Care visit (family medicine, internal medicine, etc.)

No

No

Q34	Q19	Q20	Q31	Q36	Q36_8_TEX	Q22	Q23	Q24	Q28	Q37	Q37_9_TEX	Q26
Since the ir	Since May :	Since May :	Since May :	Since May :	Since May :	I feel comfo	I have stabl	If suggeste	Would you	What servic	What servic	What conc
	No					Somewhat agree	Somewhat agree	Strongly agree	Yes	Behavioral/Mental health therapy,Surgery follow-up,Appointment to discuss and adjust medications only		I prefer in-person care,I lack knowledge/information on telehealth programs

Utilized telehealth more since November 2021	Yes	6-10	Video appointment from home	Behavioral/Mental health therapy		Strongly agree	Strongly agree	Strongly agree	Yes	Behavioral/Mental health therapy,Surgery follow-up,Primary Care visit (family medicine, internal medicine, etc.),Appointment to discuss and adjust medications only		None

Utilized telehealth more since November 2021	Yes	1-5	Video appointment from home	Physical therapy (guided exercises, post-operation therapy, etc.)		Somewhat disagree	Neither agree nor disagree	Somewhat agree	Maybe	Physical therapy (guided exercises, post-operation therapy, etc.),Primary Care visit (family medicine, internal medicine, etc.)		I prefer in-person care

Utilized telehealth more before November 2021	No						Strongly disagree	Neither agree nor disagree	Strongly disagree	No			I prefer in-person care,I think telehealth would have a lower quality of care

Utilized telehealth more since November 2021	Yes	6-10	Video from physician's office to another provider,Telephone appointment	Behavioral /Mental health therapy		Somewhat disagree	Strongly disagree	Neither agree nor disagree	Yes	Behavioral /Mental health therapy,Physical therapy (guided exercises, post-operation therapy, etc.),Primary Care visit (family medicine, internal medicine, etc.),Appointment to discuss and adjust medications only		I do not have quality internet service,I prefer in-person care,I have a lack of privacy at home

Utilized telehealth roughly the same before and after November 2021	Yes	1-5	Telephone appointment	Primary Care visit (family medicine, internal medicine, etc.),Appointment to discuss and adjust medications only		Strongly agree	Somewhat disagree	Somewhat agree	Yes	Physical therapy (guided exercises, post-operation therapy, etc.),Surgery follow-up,Primary Care visit (family medicine, internal medicine, etc.),Appointment to discuss and adjust medications only		I do not have quality internet service

Utilized telehealth roughly the same before and after November 2021	No					Strongly agree	Somewhat agree	Somewhat agree	Yes	Primary Care visit (family medicine, internal medicine, etc.),Appointment to discuss and adjust medications only		I prefer in-person care

No					Neither agree nor disagree	Strongly agree	Strongly agree	Maybe	Behavioral/Mental health therapy,Physical therapy (guided exercises, post-operation therapy, etc.),Surgery follow-up,Primary Care visit (family medicine, internal medicine, etc.),Substance abuse treatment program,Specialty care (cardiologist,		I prefer in-person care

No	Somewhat disagree	Somewhat agree	Somewhat disagree	Maybe	Behavioral/Mental health therapy,Appointment to discuss and adjust medications only	I prefer in-person care,I think telehealth would have a lower quality of care

No		Strongly agree	Strongly agree	Strongly agree	Yes	Behavioral/Mental health therapy,Primary Care visit (family medicine, internal medicine, etc.),Specialty care (cardiologist, pulmonologist, nephrologist, etc)	None

Utilized telehealth roughly the same before and after November 2021	No					Strongly agree	Somewhat agree	Strongly agree	Yes	Behavioral/Mental health therapy,Physical therapy (guided exercises, post-operation therapy, etc.),Surgery follow-up,Primary Care visit (family medicine, internal medicine, etc.),Appointment to discuss and adjust medications only		I do not have quality internet service

No	Neither agree nor disagree	Somewhat agree	Somewhat agree	Yes	Behavioral/Mental health therapy,Surgery follow-up,Primary Care visit (family medicine, internal medicine, etc.),Appointment to discuss and adjust medications only	I prefer in-person care

No					Somewhat agree	Somewhat agree	Neither agree nor disagree	Yes	Behavioral/Mental health therapy,Physical therapy (guided exercises, post-operation therapy, etc.),Surgery follow-up,Primary Care visit (family medicine, internal medicine, etc.),Substance abuse treatment program,Specialty care (cardiologist,		I think telehealth would have a lower quality of care

No						Neither agree nor disagree	Strongly agree	Strongly agree	Yes	Behavioral /Mental health therapy,Surgery follow-up,Appointment to discuss and adjust medications only		I think telehealth would have a lower quality of care

No	Somewhat agree	Strongly disagree	Strongly disagree	Maybe	Behavioral/Mental health therapy,Primary Care visit (family medicine, internal medicine, etc.),Specialty care (cardiologist, pulmonologist, nephrologist, etc),Appointment to discuss and adjust medications only	I do not have quality internet service

No	Strongly agree	Strongly disagree	Strongly disagree	Yes	Behavioral/Mental health therapy,Primary Care visit (family medicine, internal medicine, etc.),Appointment to discuss and adjust medications only	I do not have quality internet service
No	Somewhat disagree	Somewhat disagree	Strongly disagree	No		I prefer in-person care

No					Somewhat agree	Somewhat disagree	Somewhat disagree	Maybe	Appointment to discuss and adjust medications only		I do not have quality internet service,I prefer in-person care,I think telehealth would have a lower quality of care
No					Neither agree nor disagree	Neither agree nor disagree	Somewhat disagree	Maybe	Surgery follow-up		I prefer in-person care

Utilized telehealth roughly the same before and after November 2021	Yes	1-5	Video appointment from home	Behavioral /Mental health therapy		Strongly disagree	Somewhat disagree	Strongly disagree	No			I prefer in-person care,I think telehealth would have a lower quality of care

Utilized telehealth roughly the same before and after November 2021	Yes	1-5	Video appointment from home,Telephone appointment	Behavioral/Mental health therapy,Primary Care visit (family medicine, internal medicine, etc.)		Strongly agree	Somewhat agree	Strongly agree	Yes	Behavioral/Mental health therapy,Primary Care visit (family medicine, internal medicine, etc.)		None	

	No					Somewhat agree	Strongly agree	Strongly agree	Maybe	Behavioral/Mental health therapy,Surgery follow-up,Appointment to discuss and adjust medications only		None
Utilized telehealth roughly the same before and after November 2021	No					Somewhat disagree	Strongly agree	Somewhat agree	Maybe	Appointment to discuss and adjust medications only		I prefer in-person care

Utilized telehealth roughly the same before and after November 2021	Yes	1-5	Video appointment from home	Specialty care (cardiologist, pulmonologist, nephrologist, etc)		Strongly disagree	Neither agree nor disagree	Strongly agree	Yes	Behavioral/Mental health therapy,Specialty care (cardiologist, pulmonologist, nephrologist, etc),Appointment to discuss and adjust medications only		None

Utilized telehealth more before November 2021	No					Strongly agree	Strongly agree	Strongly agree	Yes	Primary Care visit (family medicine, internal medicine, etc.)		None

Utilized telehealth roughly the same before and after November 2021	No						Somewhat agree	Strongly agree	Somewhat agree	Yes	Behavioral/Mental health therapy,Substance abuse treatment program,Appointment to discuss and adjust medications only		Other (please specify)

Utilized telehealth more since November 2021	No					Somewhat agree	Somewhat agree	Somewhat agree	Yes	Primary Care visit (family medicine, internal medicine, etc.),Specialty care (cardiologist, pulmonologist, nephrologist, etc),Appointment to discuss and adjust medications only		None

Yes	1-5	Video appointment from home	Specialty care (cardiologist, pulmonologist, nephrologist, etc)		Somewhat agree	Strongly agree	Strongly agree	Yes	Surgery follow-up,Specialty care (cardiologist, pulmonologist, nephrologist, etc),Appointment to discuss and adjust medications only		None

	No					Somewhat agree	Strongly agree	Strongly agree	Yes	Primary Care visit (family medicine, internal medicine, etc.),Appointment to discuss and adjust medications only		None
Utilized telehealth roughly the same before and after November 2021	No					Neither agree nor disagree	Strongly agree	Strongly agree	Yes	Primary Care visit (family medicine, internal medicine, etc.),Appointment to discuss and adjust medications only		I prefer in-person care

Utilized telehealth roughly the same before and after November 2021	No					Somewhat agree	Strongly agree	Somewhat agree	Yes	Behavioral/Mental health therapy,Surgery follow-up,Primary Care visit (family medicine, internal medicine, etc.),Substance abuse treatment program,Appointment to discuss and adjust medications only		I think telehealth would have a lower quality of care

Utilized telehealth roughly the same before and after November 2021	No					Somewhat agree	Neither agree nor disagree	Somewhat agree	Yes	Behavioral/Mental health therapy,Surgery follow-up,Primary Care visit (family medicine, internal medicine, etc.),Substance abuse treatment program,Specialty care (cardiologist, pulmonologist, nephrologist, etc),Appointment to discuss		I do not have quality internet service

No		Strongly disagree	Strongly disagree	Somewhat agree	Maybe	Primary Care visit (family medicine, internal medicine, etc.)	I prefer in-person care
No		Somewhat agree	Strongly agree	Strongly agree	Yes	Appointment to discuss and adjust medications only	None
No		Neither agree nor disagree	Strongly agree	Somewhat agree	Maybe	Behavioral/Mental health therapy,Appointment to discuss and adjust medications only	I think telehealth would have a lower quality of care

Utilized telehealth more before November 2021	No					Somewhat disagree	Strongly disagree	Neither agree nor disagree	Maybe	None		I prefer in-person care,I think telehealth would have a lower quality of care

No					Somewhat agree	Strongly agree	Strongly agree	Yes	Behavioral/Mental health therapy,Physical therapy (guided exercises, post-operation therapy, etc.),Surgery follow-up,Primary Care visit (family medicine, internal medicine, etc.),Substance abuse treatment program,Specialty care (cardiologist,		I prefer in-person care,I think telehealth would have a lower quality of care

Utilized telehealth more before November 2021	No					Neither agree nor disagree	Somewhat disagree	Somewhat agree	Maybe	Substance abuse treatment program,Appointment to discuss and adjust medications only		I think telehealth would have a lower quality of care,I have a lack of privacy at home
	No					Neither agree nor disagree	Strongly agree	Neither agree nor disagree	Maybe	None		I prefer in-person care

Utilized telehealth roughly the same before and after November 2021	Yes			Behavioral/Mental health therapy,Surgery follow-up		Somewhat agree	Strongly disagree	Somewhat agree	Yes	Behavioral/Mental health therapy,Appointment to discuss and adjust medications only		I do not have quality internet service

Yes	1-5	Video appointment from home	Primary Care visit (family medicine, internal medicine, etc.)		Somewhat agree	Strongly agree	Somewhat agree	Yes	Behavioral/Mental health therapy,Surgery follow-up,Primary Care visit (family medicine, internal medicine, etc.)		I think telehealth would have a lower quality of care

No	Somewhat agree	Strongly agree	Somewhat agree	Yes	Primary Care visit (family medicine, internal medicine, etc.)	I prefer in-person care,I think telehealth would have a lower quality of care

No		Somewhat agree	Strongly agree	Somewhat agree	Yes	Behavioral/Mental health therapy,Physical therapy (guided exercises, post-operation therapy, etc.),Surgery follow-up,Primary Care visit (family medicine, internal medicine, etc.),Appointment to discuss and adjust medications only	I prefer in-person care

Utilized telehealth roughly the same before and after November 2021	No					Somewhat agree	Strongly agree	Strongly agree	Yes	Behavioral/Mental health therapy,Primary Care visit (family medicine, internal medicine, etc.),Appointment to discuss and adjust medications only		I prefer in-person care,I lack knowledge/information on telehealth programs

Utilized telehealth roughly the same before and after November 2021	No					Somewhat agree	Somewhat agree	Somewhat agree	Yes	Primary Care visit (family medicine, internal medicine, etc.),Appointment to discuss and adjust medications only		I prefer in-person care,I think telehealth would have a lower quality of care

No	Somewhat disagree	Neither agree nor disagree	Somewhat disagree	Maybe	Appointment to discuss and adjust medications only	I prefer in-person care,I think telehealth would have a lower quality of care
No	Neither agree nor disagree	Strongly agree	Strongly agree	Yes	Physical therapy (guided exercises, post-operation therapy, etc.),Primary Care visit (family medicine, internal medicine, etc.)	I think telehealth would have a lower quality of care

No	Somewhat agree	Strongly agree	Somewhat agree	Yes	Behavioral/Mental health therapy,Physical therapy (guided exercises, post-operation therapy, etc.),Primary Care visit (family medicine, internal medicine, etc.),Appointment to discuss and adjust medications only	Other (please specify)

No					Somewhat agree	Strongly agree	Strongly agree	Yes	Behavioral/Mental health therapy,Physical therapy (guided exercises, post-operation therapy, etc.),Surgery follow-up,Primary Care visit (family medicine, internal medicine, etc.),Substance abuse treatment program,Appointment to discuss and adjust		None

Utilized telehealth roughly the same before and after November 2021	No					Somewhat agree	Somewhat agree	Strongly agree	Yes	Physical therapy (guided exercises, post-operation therapy, etc.),Primary Care visit (family medicine, internal medicine, etc.),Appointment to discuss and adjust medications only		I think telehealth would have a lower quality of care
	No					Somewhat disagree	Somewhat agree	Somewhat disagree	Maybe	None		I prefer in-person care

Utilized telehealth more since November 2021	No					Somewhat agree	Somewhat agree	Somewhat agree	Yes	Behavioral/Mental health therapy,Primary Care visit (family medicine, internal medicine, etc.),Appointment to discuss and adjust medications only		I think telehealth would have a lower quality of care

Utilized telehealth more before November 2021	No					Strongly disagree	Strongly agree	Strongly disagree	No			I prefer in-person care,I think telehealth would have a lower quality of care

	No					Somewhat agree	Strongly agree	Strongly agree	Yes	Behavioral/Mental health therapy,Surgery follow-up,Primary Care visit (family medicine, internal medicine, etc.)		I prefer in-person care
Utilized telehealth roughly the same before and after November 2021	No					Neither agree nor disagree	Somewhat disagree	Somewhat agree	Yes	Behavioral/Mental health therapy,Surgery follow-up,Appointment to discuss and adjust medications only		I do not have quality internet service,I prefer in-person care

Utilized telehealth roughly the same before and after November 2021	No					Somewhat agree	Somewhat disagree	Somewhat agree	Maybe	Surgery follow-up,Primary Care visit (family medicine, internal medicine, etc.),Appointment to discuss and adjust medications only		I do not have quality internet service

Utilized telehealth roughly the same before and after November 2021	No					Strongly agree	Strongly agree	Somewhat agree	Yes	Surgery follow-up,Primary Care visit (family medicine, internal medicine, etc.),Specialty care (cardiologist, pulmonologist, nephrologist, etc),Appointment to discuss and adjust medications only		I lack knowledge/information on telehealth programs

Utilized telehealth roughly the same before and after November 2021	No					Somewhat agree	Strongly agree	Somewhat agree	Yes	Surgery follow-up,Primary Care visit (family medicine, internal medicine, etc.),Appointment to discuss and adjust medications only		I think telehealth would have a lower quality of care

Utilized telehealth roughly the same before and after November 2021	No					Neither agree nor disagree	Neither agree nor disagree	Neither agree nor disagree	Yes	Behavioral/Mental health therapy,Primary Care visit (family medicine, internal medicine, etc.),Specialty care (cardiologist, pulmonologist, nephrologist, etc),Appointment to discuss and adjust medications only		I prefer in-person care,I think telehealth would have a lower quality of care

No	Neither agree nor disagree	Neither agree nor disagree	Neither agree nor disagree	Maybe	Primary Care visit (family medicine, internal medicine, etc.),Appointment to discuss and adjust medications only	I do not have quality internet service,I prefer in-person care

Utilized telehealth roughly the same before and after November 2021	Yes	1-5	Video from physician's office to another provider	Specialty care (cardiologist, pulmonologist, nephrologist, etc)		Somewhat agree	Somewhat agree	Somewhat agree	Yes	Surgery follow-up,Primary Care visit (family medicine, internal medicine, etc.),Appointment to discuss and adjust medications only		I prefer in-person care

No		Somewhat disagree	Somewhat agree	Somewhat agree	Yes	Primary Care visit (family medicine, internal medicine, etc.),Appointment to discuss and adjust medications only	I prefer in-person care,I think telehealth would have a lower quality of care,I lack knowledge/information on telehealth programs

Utilized telehealth roughly the same before and after November 2021	No	Strongly agree	Strongly agree	Strongly agree	Yes	Behavioral/Mental health therapy,Primary Care visit (family medicine, internal medicine, etc.),Appointment to discuss and adjust medications only	I prefer in-person care,I think telehealth would have a lower quality of care

Utilized telehealth roughly the same before and after November 2021	No					Neither agree nor disagree	Strongly disagree	Neither agree nor disagree	Yes	Primary Care visit (family medicine, internal medicine, etc.),Appointment to discuss and adjust medications only		I prefer in-person care,I think telehealth would have a lower quality of care
	No					Neither agree nor disagree	Strongly agree	Somewhat disagree	Maybe	Behavioral/Mental health therapy,Surgery follow-up,Appointment to discuss and adjust medications only		I prefer in-person care

Utilized telehealth roughly the same before and after November 2021	No					Somewhat agree	Somewhat agree	Strongly agree	Yes	Behavioral/Mental health therapy,Primary Care visit (family medicine, internal medicine, etc.),Appointment to discuss and adjust medications only		None

No					Somewhat disagree	Strongly agree	Strongly agree	Yes	Other (please specify)	Limited follow up appointment	I think telehealth would have a lower quality of care

No	Somewhat agree	Strongly agree	Somewhat agree	Yes	Behavioral/Mental health therapy,Physical therapy (guided exercises, post-operation therapy, etc.),Appointment to discuss and adjust medications only		I prefer in-person care,I think telehealth would have a lower quality of care

No		Somewhat disagree	Somewhat disagree	Strongly disagree	Maybe	Primary Care visit (family medicine, internal medicine, etc.)	I prefer in-person care
No		Somewhat agree	Somewhat agree	Somewhat agree	Yes	Physical therapy (guided exercises, post-operation therapy, etc.),Surgery follow-up,Primary Care visit (family medicine, internal medicine, etc.)	None

No		Somewhat disagree	Somewhat agree	Somewhat agree	Maybe	Primary Care visit (family medicine, internal medicine, etc.)		I prefer in-person care,I think telehealth would have a lower quality of care
No		Strongly disagree	Strongly disagree	Somewhat disagree	Maybe	Surgery follow-up,Appointment to discuss and adjust medications only		I do not have quality internet service,I think telehealth would have a lower quality of care

Utilized telehealth roughly the same before and after November 2021	Yes	1-5	Video appointment from home	Behavioral/Mental health therapy		Somewhat agree	Somewhat disagree	Strongly agree	Yes	Behavioral/Mental health therapy,Surgery follow-up		I think telehealth would have a lower quality of care
Utilized telehealth roughly the same before and after November 2021	No					Neither agree nor disagree	Neither agree nor disagree	Neither agree nor disagree	No			I prefer in-person care
	No					Somewhat agree	Strongly agree	Somewhat disagree	Maybe	Surgery follow-up,Appointment to discuss and adjust medications only		I prefer in-person care

Utilized telehealth more since November 2021	Yes	1-5	Video appointment from home	Primary Care visit (family medicine, internal medicine, etc.)		Somewhat agree	Somewhat agree	Somewhat agree	Yes	Physical therapy (guided exercises, post-operation therapy, etc.),Primary Care visit (family medicine, internal medicine, etc.),Appointment to discuss and adjust medications only		I prefer in-person care,I think telehealth would have a lower quality of care

Utilized telehealth more before November 2021	No					Strongly agree	Somewhat agree	Somewhat agree	Yes	Primary Care visit (family medicine, internal medicine, etc.)		I prefer in-person care,I think telehealth would have a lower quality of care
	No					Neither agree nor disagree	Strongly disagree	Neither agree nor disagree	Yes	Surgery follow-up		None

Utilized telehealth more before November 2021	Yes	1-5	Video from physician's office to another provider	Specialty care (cardiologist, pulmonologist, nephrologist, etc)		Somewhat disagree	Strongly agree	Somewhat disagree	Maybe	Appointment to discuss and adjust medications only		I prefer in-person care
	No					Somewhat agree	Somewhat agree	Somewhat agree	Maybe	Primary Care visit (family medicine, internal medicine, etc.)		I prefer in-person care
	No					Somewhat agree	Somewhat agree	Neither agree nor disagree	Maybe	Primary Care visit (family medicine, internal medicine, etc.)		I prefer in-person care

No		Neither agree nor disagree	Strongly agree	Somewhat agree	Yes	Primary Care visit (family medicine, internal medicine, etc.)	None

Utilized telehealth roughly the same before and after November 2021	No					Strongly disagree	Strongly disagree	Strongly disagree	Yes	Behavioral /Mental health therapy,Surgery follow-up,Primary Care visit (family medicine, internal medicine, etc.),Appointment to discuss and adjust medications only		None

Utilized telehealth more since November 2021	Yes	1-5	Video appointment from home,Video from physician's office to another provider	Specialty care (cardiologist, pulmonologist, nephrologist, etc)		Strongly agree	Strongly agree	Somewhat agree	Yes	Primary Care visit (family medicine, internal medicine, etc.),Specialty care (cardiologist, pulmonologist, nephrologist, etc),Appointment to discuss and adjust medications only		Other (please specify)

Utilized telehealth roughly the same before and after November 2021	No	Somewhat agree	Strongly agree	Somewhat agree	Yes	Primary Care visit (family medicine, internal medicine, etc.)	I prefer in-person care,I think telehealth would have a lower quality of care
	No	Strongly disagree	Strongly disagree	Strongly disagree	No		I do not have quality internet service,I prefer in-person care,I think telehealth would have a lower quality of care

No					Neither agree nor disagree	Somewhat agree	Somewhat agree	Maybe	Behavioral/Mental health therapy,Appointment to discuss and adjust medications only		I lack knowledge/information on telehealth programs
No					Somewhat agree	Somewhat agree	Somewhat agree	Yes	Other (please specify)	Unsure	None

Utilized telehealth roughly the same before and after November 2021	No					Strongly agree	Strongly agree	Strongly agree	Yes	Behavioral/Mental health therapy,Physical therapy (guided exercises, post-operation therapy, etc.),Surgery follow-up,Specialty care (cardiologist, pulmonologist, nephrologist, etc),Appointment to discuss and adjust medications only		I think telehealth would have a lower quality of care

Utilized telehealth roughly the same before and after November 2021	Yes	1-5	Video appointment from home	Behavioral /Mental health therapy		Strongly agree	Strongly agree	Strongly agree	Yes	Behavioral /Mental health therapy		None
	No					Neither agree nor disagree	Neither agree nor disagree	Neither agree nor disagree	No			None

No	Somewhat disagree	Strongly agree	Somewhat disagree	Maybe	None		I prefer in-person care,I think telehealth would have a lower quality of care

Utilized telehealth more since November 2021	No					Strongly agree	Strongly agree	Strongly agree	Yes	Primary Care visit (family medicine, internal medicine, etc.),Specialty care (cardiologist, pulmonologist, nephrologist, etc)		None

Yes	1-5	Telephone appointment	Other (please specify)	None	Somewhat agree	Somewhat disagree	Somewhat agree	Maybe	Primary Care visit (family medicine, internal medicine, etc.),Appointment to discuss and adjust medications only		None
No					Neither agree nor disagree	Strongly agree	Somewhat disagree	Maybe	Primary Care visit (family medicine, internal medicine, etc.),Appointment to discuss and adjust medications only		I prefer in-person care,I think telehealth would have a lower quality of care,I lack knowledge/information on telehealth programs

Somewhat disagree	Strongly disagree	Somewhat agree	Yes	Behavioral/Mental health therapy,Physical therapy (guided exercises, post-operation therapy, etc.),Appointment to discuss and adjust medications only	I do not have quality internet service,I think telehealth would have a lower quality of care

Utilized telehealth roughly the same before and after November 2021	No					Somewhat disagree	Strongly agree	Somewhat agree	Maybe	Surgery follow-up,Primary Care visit (family medicine, internal medicine, etc.),Appointment to discuss and adjust medications only		I prefer in-person care,I think telehealth would have a lower quality of care
	No					Neither agree nor disagree	Strongly agree	Neither agree nor disagree	Maybe	Appointment to discuss and adjust medications only		I prefer in-person care

Utilized telehealth roughly the same before and after November 2021	No					Strongly agree	Strongly agree	Strongly agree	Yes	Physical therapy (guided exercises, post-operation therapy, etc.),Surgery follow-up,Primary Care visit (family medicine, internal medicine, etc.)		None

Utilized telehealth roughly the same before and after November 2021	No					Strongly agree	Strongly agree	Strongly agree	Yes	Physical therapy (guided exercises, post-operation therapy, etc.),Surgery follow-up,Primary Care visit (family medicine, internal medicine, etc.),Specialty care (cardiologist, pulmonologist, nephrologist, etc),Appointment to discuss and adjust medicatio		None

No	Strongly agree	Strongly agree	Strongly agree	Yes	Behavioral/Mental health therapy,Primary Care visit (family medicine, internal medicine, etc.),Appointment to discuss and adjust medications only	None

No		Somewhat agree	Somewhat disagree	Somewhat agree	Yes	Primary Care visit (family medicine, internal medicine, etc.)	I prefer in-person care

Yes	1-5	Video appointment from home,Telephone appointment	Specialty care (cardiologist, pulmonologist, nephrologist, etc),Appointment to discuss and adjust medications only		Somewhat agree	Somewhat agree	Somewhat agree	Yes	Behavioral/Mental health therapy,Specialty care (cardiologist, pulmonologist, nephrologist, etc),Appointment to discuss and adjust medications only		I prefer in-person care,I think telehealth would have a lower quality of care

No					Strongly disagree	Strongly disagree	Strongly disagree	No			I prefer in-person care
Yes	1-5	Telephone appointment	Other (please specify)	Tel-A-Doc appointment	Somewhat agree	Somewhat agree	Somewhat agree	Yes	Behavioral/Mental health therapy,Physical therapy (guided exercises, post-operation therapy, etc.),Surgery follow-up,Primary Care visit (family medicine, internal medicine, etc.)		I prefer in-person care

Utilized telehealth roughly the same before and after November 2021	No					Strongly agree	Somewhat agree	Strongly agree	Yes	Primary Care visit (family medicine, internal medicine, etc.),Appointment to discuss and adjust medications only		I prefer in-person care

Utilized telehealth roughly the same before and after November 2021	No					Somewhat disagree	Strongly agree	Somewhat agree	Yes	Behavioral/Mental health therapy,Appointment to discuss and adjust medications only		I prefer in-person care,I think telehealth would have a lower quality of care

No					Somewhat agree	Somewhat agree	Neither agree nor disagree	Maybe	Behavioral/Mental health therapy,Primary Care visit (family medicine, internal medicine, etc.),Appointment to discuss and adjust medications only		I prefer in-person care

No		Somewhat agree	Strongly agree	Somewhat agree	Yes	Physical therapy (guided exercises, post-operation therapy, etc.),Surgery follow-up,Appointment to discuss and adjust medications only	I think telehealth would have a lower quality of care

Utilized telehealth roughly the same before and after November 2021	No	Somewhat agree	Strongly agree	Somewhat agree	Maybe	Behavioral/Mental health therapy,Physical therapy (guided exercises, post-operation therapy, etc.),Appointment to discuss and adjust medications only	I prefer in-person care,I think telehealth would have a lower quality of care,Other (please specify)

Utilized telehealth roughly the same before and after November 2021	No					Somewhat agree	Neither agree nor disagree	Somewhat agree	Yes	Behavioral/Mental health therapy,Primary Care visit (family medicine, internal medicine, etc.),Appointment to discuss and adjust medications only		I prefer in-person care,I think telehealth would have a lower quality of care,I lack knowledge/information on telehealth programs

No					Neither agree nor disagree	Strongly agree	Neither agree nor disagree	Maybe	None		I prefer in-person care
Yes	1-5	Video appointment from home	Behavioral/Mental health therapy		Strongly disagree	Neither agree nor disagree	Strongly disagree	Yes	Behavioral/Mental health therapy,Primary Care visit (family medicine, internal medicine, etc.),Appointment to discuss and adjust medications only		None

Utilized telehealth more before November 2021	Yes	1-5	Video appointment from home	Primary Care visit (family medicine, internal medicine, etc.)		Strongly agree	Strongly agree	Strongly agree	Yes	Primary Care visit (family medicine, internal medicine, etc.),Specialty care (cardiologist, pulmonologist, nephrologist, etc)		None

Utilized telehealth roughly the same before and after November 2021	Yes	1-5	Video from physician's office to another provider	Other (please specify)	MFM-Obstetrics	Somewhat disagree	Strongly disagree	Somewhat agree	Yes	Primary Care visit (family medicine, internal medicine, etc.),Appointment to discuss and adjust medications only		I do not have quality internet service,Other (please specify)

Utilized telehealth roughly the same before and after November 2021	Yes	1-5	Telephone appointment	Primary Care visit (family medicine, internal medicine, etc.)		Somewhat agree	Somewhat agree	Somewhat agree	Yes	Primary Care visit (family medicine, internal medicine, etc.)		I prefer in-person care

No		Neither agree nor disagree	Neither agree nor disagree	Somewhat disagree	Maybe	None		I prefer in-person care
No		Somewhat agree	Strongly agree	Somewhat agree	Maybe	Behavioral/Mental health therapy,Primary Care visit (family medicine, internal medicine, etc.)		I think telehealth would have a lower quality of care

	No					Neither agree nor disagree	Strongly agree	Strongly agree	Yes	Primary Care visit (family medicine, internal medicine, etc.)		None
Utilized telehealth roughly the same before and after November 2021	Yes	1-5	Video appointment from home	Other (please specify)	Class to go over upcoming surgery.	Strongly agree	Strongly agree	Strongly agree	Yes	Behavioral/Mental health therapy,Surgery follow-up,Primary Care visit (family medicine, internal medicine, etc.)		None

Utilized telehealth more since November 2021	No					Somewhat disagree	Somewhat disagree	Neither agree nor disagree	Yes	Appointment to discuss and adjust medications only		I prefer in-person care,I think telehealth would have a lower quality of care
	No					Strongly disagree	Somewhat agree	Somewhat agree	Maybe	Surgery follow-up,Primary Care visit (family medicine, internal medicine, etc.)		I prefer in-person care,I think telehealth would have a lower quality of care

Utilized telehealth roughly the same before and after November 2021	No					Somewhat disagree	Strongly agree	Somewhat disagree	No			I prefer in-person care,I think telehealth would have a lower quality of care
	No					Neither agree nor disagree	Neither agree nor disagree	Strongly agree	Yes	Physical therapy (guided exercises, post-operation therapy, etc.),Surgery follow-up		I prefer in-person care

Utilized telehealth roughly the same before and after November 2021	Yes	1-5	Video appointment from home	Behavioral /Mental health therapy		Somewhat agree	Somewhat agree	Strongly agree	Yes	Behavioral /Mental health therapy,Primary Care visit (family medicine, internal medicine, etc.),Substance abuse treatment program		I prefer in-person care

No	Somewhat agree	Strongly agree	Strongly agree	Yes	Primary Care visit (family medicine, internal medicine, etc.),Appointment to discuss and adjust medications only	I prefer in-person care,I think telehealth would have a lower quality of care

Utilized telehealth roughly the same before and after November 2021	Yes	1-5	Video appointment from home	Behavioral/Mental health therapy,Appointment to discuss and adjust medications only		Somewhat agree	Somewhat disagree	Strongly agree	Yes	Behavioral/Mental health therapy,Appointment to discuss and adjust medications only		I do not have quality internet service

Utilized telehealth roughly the same before and after November 2021	No					Strongly disagree	Somewhat disagree	Strongly disagree	No			I do not have quality internet service,I prefer in-person care,I think telehealth would have a lower quality of care

Utilized telehealth more since November 2021	Yes	1-5	Video appointment from home	Primary Care visit (family medicine, internal medicine, etc.)		Somewhat agree	Strongly agree	Somewhat agree	Yes	Surgery follow-up,Primary Care visit (family medicine, internal medicine, etc.),Appointment to discuss and adjust medications only		I prefer in-person care
	No					Somewhat disagree	Somewhat disagree	Somewhat agree	Maybe	Appointment to discuss and adjust medications only		I do not have quality internet service,I prefer in-person care,I think telehealth would have a lower quality of care

No					Somewhat disagree	Somewhat agree	Strongly disagree	Maybe	Surgery follow-up,Appointment to discuss and adjust medications only		I prefer in-person care
Yes	1-5	Video appointment from home	Specialty care (cardiologist, pulmonologist, nephrologist, etc),Other (please specify)	Urgent care visit	Somewhat agree	Strongly agree	Somewhat agree	Yes	Other (please specify)	Travel to specialist is greater than 3hrs, when urgent care is closed or Out of town and need appointment	I prefer in-person care

Yes	1-5	Video appointment from home	Primary Care visit (family medicine, internal medicine, etc.)		Strongly agree	Strongly agree	Strongly agree	Yes	Behavioral/Mental health therapy,Primary Care visit (family medicine, internal medicine, etc.),Substance abuse treatment program,Appointment to discuss and adjust medications only		I prefer in-person care,I have a lack of privacy at home

No	Somewhat disagree	Somewhat agree	Strongly agree	Maybe	Behavioral/Mental health therapy,Physical therapy (guided exercises, post-operation therapy, etc.),Primary Care visit (family medicine, internal medicine, etc.),Appointment to discuss and adjust medications only	I think telehealth would have a lower quality of care,I lack knowledge/information on telehealth programs, I have a lack of privacy at home

Utilized telehealth roughly the same before and after November 2021	Yes	1-5	Video appointment from home,Telephone appointment	Behavioral/Mental health therapy,Primary Care visit (family medicine, internal medicine, etc.)		Strongly agree	Strongly agree	Strongly agree	Yes	Behavioral/Mental health therapy,Surgery follow-up,Primary Care visit (family medicine, internal medicine, etc.),Specialty care (cardiologist, pulmonologist, nephrologist, etc)		None

No	Somewhat agree	Somewhat agree	Strongly agree	Yes	Behavioral/Mental health therapy,Surgery follow-up,Primary Care visit (family medicine, internal medicine, etc.)	I prefer in-person care
No	Strongly disagree	Strongly disagree	Strongly disagree	No		I do not have quality internet service,I prefer in-person care

Utilized telehealth roughly the same before and after November 2021	No					Somewhat agree	Somewhat agree	Somewhat agree	Yes	Behavioral/Mental health therapy,Primary Care visit (family medicine, internal medicine, etc.),Appointment to discuss and adjust medications only		I think telehealth would have a lower quality of care

No	Strongly agree	Strongly agree	Strongly agree	Yes	Behavioral/Mental health therapy,Surgery follow-up,Appointment to discuss and adjust medications only		I prefer in-person care
No	Neither agree nor disagree	Strongly disagree	Strongly disagree	No			I do not have quality internet service,I prefer in-person care

No					Somewhat agree	Somewhat agree	Somewhat agree	Yes	Physical therapy (guided exercises, post-operation therapy, etc.),Surgery follow-up,Appointment to discuss and adjust medications only		I lack knowledge/information on telehealth programs
No					Somewhat disagree	Strongly disagree	Somewhat disagree	No			I do not have quality internet service,I prefer in-person care,I think telehealth would have a lower quality of care

	No					Neither agree nor disagree	Somewhat agree	Strongly agree	Yes	Primary Care visit (family medicine, internal medicine, etc.)		I lack knowledge/information on telehealth programs
Utilized telehealth more before November 2021	No					Somewhat agree	Strongly agree	Strongly agree	Yes	Behavioral/Mental health therapy,Primary Care visit (family medicine, internal medicine, etc.)		I prefer in-person care,I have a lack of privacy at home

No					Somewhat disagree	Strongly agree	Somewhat agree	Maybe	Surgery follow-up,Primary Care visit (family medicine, internal medicine, etc.)		I prefer in-person care,I think telehealth would have a lower quality of care,I have a lack of privacy at home
No					Strongly disagree	Strongly agree	Strongly disagree	No			I lack knowledge/information on telehealth programs

Utilized telehealth more since November 2021	No					Strongly agree	Strongly agree	Strongly agree	Yes	Behavioral /Mental health therapy,Physical therapy (guided exercises, post-operation therapy, etc.),Surgery follow-up,Primary Care visit (family medicine, internal medicine, etc.),Substance abuse treatment program,Specialty care (cardiologist,		None

Utilized telehealth roughly the same before and after November 2021	No					Somewhat agree	Somewhat agree	Somewhat agree	Maybe	Primary Care visit (family medicine, internal medicine, etc.)		I prefer in-person care
	No					Neither agree nor disagree	Strongly agree	Strongly agree	Maybe	Appointment to discuss and adjust medications only		I prefer in-person care,Other (please specify)

Utilized telehealth more since November 2021	Yes	6-10	Video appointment from home,Telephone appointment	Primary Care visit (family medicine, internal medicine, etc.)		Strongly agree	Strongly agree	Strongly agree	Yes	Behavioral/Mental health therapy,Primary Care visit (family medicine, internal medicine, etc.),Appointment to discuss and adjust medications only		None

Yes	1-5	Video appointment from home,Telephone appointment	Behavioral/Mental health therapy,Primary Care visit (family medicine, internal medicine, etc.)		Strongly agree	Strongly agree	Neither agree nor disagree	Yes	Behavioral/Mental health therapy,Primary Care visit (family medicine, internal medicine, etc.)		None

No					Somewhat agree	Somewhat agree	Strongly agree	Maybe	Surgery follow-up,Primary Care visit (family medicine, internal medicine, etc.),Appointment to discuss and adjust medications only		I do not have quality internet service,I prefer in-person care

No	Neither agree nor disagree	Somewhat disagree	Somewhat agree	Yes	Behavioral/Mental health therapy,Surgery follow-up,Primary Care visit (family medicine, internal medicine, etc.),Appointment to discuss and adjust medications only	I do not have quality internet service,I prefer in-person care,I have a lack of privacy at home
No	Neither agree nor disagree	Somewhat disagree	Neither agree nor disagree	Maybe	Appointment to discuss and adjust medications only	I prefer in-person care

Utilized telehealth more since November 2021	Yes	1-5	Video appointment from home	Other (please specify)	Functional medicine	Somewhat agree	Strongly agree	Somewhat agree	Yes	Primary Care visit (family medicine, internal medicine, etc.)		I think telehealth would have a lower quality of care
	No					Somewhat agree	Strongly agree	Strongly agree	Yes	Primary Care visit (family medicine, internal medicine, etc.),Appointment to discuss and adjust medications only		Other (please specify)

No	Somewhat agree	Somewhat agree	Somewhat agree	Maybe	Behavioral/Mental health therapy,Surgery follow-up,Appointment to discuss and adjust medications only	I do not have quality internet service,I think telehealth would have a lower quality of care

No	Strongly disagree	Strongly agree	Somewhat disagree	No	I prefer in-person care

Yes	1-5	Video appointment from home	Other (please specify)	Sinus infection	Strongly agree	Strongly agree	Strongly agree	Yes	Physical therapy (guided exercises, post-operation therapy, etc.),Surgery follow-up,Primary Care visit (family medicine, internal medicine, etc.),Specialty care (cardiologist, pulmonologist, nephrologist, etc),Appointment to discuss and adjust medicatio		None

No	Somewhat disagree	Strongly disagree	Neither agree nor disagree	Maybe	Appointment to discuss and adjust medications only		I prefer in-person care,I think telehealth would have a lower quality of care
	Somewhat disagree	Somewhat agree	Somewhat agree	Yes	Surgery follow-up,Appointment to discuss and adjust medications only		I prefer in-person care

No		Strongly agree	Strongly agree	Strongly agree	Yes	Behavioral/Mental health therapy,Primary Care visit (family medicine, internal medicine, etc.),Appointment to discuss and adjust medications only	None

No	Neither agree nor disagree	Strongly agree	Somewhat agree	No	I lack knowledge/information on telehealth programs

Utilized telehealth more since November 2021	Yes	1-5	Video appointment from home	Specialty care (cardiologist, pulmonologist, nephrologist, etc)		Somewhat agree	Strongly agree	Strongly agree	Yes	Behavioral/Mental health therapy,Primary Care visit (family medicine, internal medicine, etc.),Specialty care (cardiologist, pulmonologist, nephrologist, etc),Appointment to discuss and adjust medications only		None

No	Somewhat agree	Somewhat agree	Somewhat agree	Yes	Surgery follow-up,Appointment to discuss and adjust medications only	I lack knowledge/information on telehealth programs

No	Somewhat disagree	Strongly disagree	Somewhat disagree	No		I do not have quality internet service,I prefer in-person care,I lack knowledge/information on telehealth programs
No	Neither agree nor disagree	Neither agree nor disagree	Somewhat agree	Yes	Primary Care visit (family medicine, internal medicine, etc.)	I lack knowledge/information on telehealth programs

No	Somewhat agree	Strongly agree	Somewhat agree	Yes	Primary Care visit (family medicine, internal medicine, etc.)		I prefer in-person care,I lack knowledge/information on telehealth programs

No					Strongly agree	Strongly agree	Neither agree nor disagree	Maybe	Primary Care visit (family medicine, internal medicine, etc.),Appointment to discuss and adjust medications only		None

No		Somewhat disagree	Strongly agree	Somewhat agree	Maybe	Appointment to discuss and adjust medications only	I prefer in-person care,I think telehealth would have a lower quality of care

No		Somewhat disagree	Strongly agree	Neither agree nor disagree	Maybe	Physical therapy (guided exercises, post-operation therapy, etc.),Appointment to discuss and adjust medications only	I prefer in-person care

Utilized telehealth more since November 2021	Yes	1-5	Video appointment from home	Primary Care visit (family medicine, internal medicine, etc.)		Somewhat agree	Strongly agree	Somewhat agree	Yes	Behavioral/Mental health therapy,Primary Care visit (family medicine, internal medicine, etc.),Appointment to discuss and adjust medications only		I prefer in-person care

Utilized telehealth roughly the same before and after November 2021	Yes	1-5	Video appointment from home	Behavioral/Mental health therapy		Neither agree nor disagree	Somewhat disagree	Somewhat agree	Yes	Behavioral/Mental health therapy,Appointment to discuss and adjust medications only		I do not have quality internet service
Utilized telehealth roughly the same before and after November 2021	No					Somewhat disagree	Strongly agree	Somewhat disagree	Maybe	Appointment to discuss and adjust medications only		I prefer in-person care,I think telehealth would have a lower quality of care

Utilized telehealth roughly the same before and after November 2021	Yes	1-5	Telephone appointment	Surgery follow-up		Somewhat disagree	Strongly disagree	Neither agree nor disagree	Maybe	Surgery follow-up,Appointment to discuss and adjust medications only		I do not have quality internet service,I prefer in-person care,I think telehealth would have a lower quality of care,I have a lack of privacy at home

Utilized telehealth roughly the same before and after November 2021	No					Neither agree nor disagree	Somewhat disagree	Somewhat disagree	No			I do not have quality internet service,I prefer in-person care,I lack knowledge/information on telehealth programs
	No					Somewhat agree	Somewhat agree	Somewhat agree	Yes	Primary Care visit (family medicine, internal medicine, etc.)		None

Utilized telehealth more since November 2021	No					Somewhat disagree	Strongly agree	Strongly disagree	No			I prefer in-person care,I think telehealth would have a lower quality of care,I have a lack of privacy at home,Other (please specify)

Utilized telehealth more since November 2021	Yes	1-5	Video appointment from home	Specialty care (cardiologist, pulmonologist, nephrologist, etc)		Somewhat agree	Strongly agree	Somewhat agree	Yes	Primary Care visit (family medicine, internal medicine, etc.),Specialty care (cardiologist, pulmonologist, nephrologist, etc),Appointment to discuss and adjust medications only		I prefer in-person care,I think telehealth would have a lower quality of care

No		Neither agree nor disagree	Strongly agree	Somewhat agree	Maybe	Surgery follow-up,Specialty care (cardiologist, pulmonologist, nephrologist, etc)	None

No					Neither agree nor disagree	Strongly disagree	Neither agree nor disagree	Maybe	Behavioral/Mental health therapy,Primary Care visit (family medicine, internal medicine, etc.)		I do not have quality internet service,I prefer in-person care,I think telehealth would have a lower quality of care

Utilized telehealth roughly the same before and after November 2021	No					Somewhat agree	Somewhat disagree	Somewhat agree	Yes	Behavioral /Mental health therapy,Physical therapy (guided exercises, post-operation therapy, etc.),Surgery follow-up,Primary Care visit (family medicine, internal medicine, etc.),Substance abuse treatment program,Specialty care (cardiologist,		I do not have quality internet service

No		Strongly disagree	Strongly disagree	Strongly disagree	No	I do not have quality internet service,I prefer in-person care,I think telehealth would have a lower quality of care
No		Somewhat disagree	Somewhat disagree	Somewhat disagree	No	I do not have quality internet service,I prefer in-person care,I think telehealth would have a lower quality of care

Yes	1-5	Video appointment from home	Behavioral/Mental health therapy		Somewhat agree	Strongly agree	Somewhat agree	Yes	Behavioral/Mental health therapy,Surgery follow-up,Appointment to discuss and adjust medications only		I prefer in-person care

No		Somewhat agree	Somewhat agree	Strongly agree	Yes	Behavioral/Mental health therapy,Primary Care visit (family medicine, internal medicine, etc.),Appointment to discuss and adjust medications only	I do not have quality internet service,I think telehealth would have a lower quality of care,I lack knowledge/information on telehealth programs

Utilized telehealth roughly the same before and after November 2021	No						Somewhat agree	Somewhat disagree	Somewhat agree	Yes	Primary Care visit (family medicine, internal medicine, etc.),Appointment to discuss and adjust medications only		I do not have quality internet service,I prefer in-person care,I think telehealth would have a lower quality of care	

No	Neither agree nor disagree	Strongly disagree	Strongly agree	Yes	Behavioral/Mental health therapy,Physical therapy (guided exercises, post-operation therapy, etc.),Surgery follow-up,Primary Care visit (family medicine, internal medicine, etc.)	None

Utilized telehealth more before November 2021	No					Somewhat agree	Strongly agree	Somewhat agree	Yes	Behavioral/Mental health therapy		I prefer in-person care,I think telehealth would have a lower quality of care,Other (please specify)

Utilized telehealth roughly the same before and after November 2021	No					Strongly agree	Strongly agree	Strongly agree	Yes	Behavioral/Mental health therapy,Primary Care visit (family medicine, internal medicine, etc.),Substance abuse treatment program,Appointment to discuss and adjust medications only		None

No					Strongly disagree	Neither agree nor disagree	Strongly disagree	No			I prefer in-person care
No					Somewhat disagree	Somewhat agree	Somewhat disagree	Maybe	Appointment to discuss and adjust medications only		I prefer in-person care
No					Somewhat agree	Strongly agree	Neither agree nor disagree	Maybe	Behavioral/Mental health therapy,Appointment to discuss and adjust medications only		I prefer in-person care

No	Somewhat agree	Somewhat agree	Somewhat agree	Maybe	Physical therapy (guided exercises, post-operation therapy, etc.)	I prefer in-person care,I think telehealth would have a lower quality of care
No	Neither agree nor disagree	Somewhat agree	Strongly agree	Yes	Primary Care visit (family medicine, internal medicine, etc.)	I lack knowledge/information on telehealth programs

No		Strongly agree	Strongly agree	Strongly agree	Yes	Behavioral/Mental health therapy,Surgery follow-up,Primary Care visit (family medicine, internal medicine, etc.),Specialty care (cardiologist, pulmonologist, nephrologist, etc)		None
Yes								

No	Somewhat agree	Neither agree nor disagree	Somewhat agree	Maybe
No				

Utilized telehealth more since November 2021	Yes											
	No					Strongly disagree	Somewhat agree	Strongly disagree	No			I prefer in-person care

	No			
Utilized telehealth roughly the same before and after November 2021	No			
	Yes	1-5	Telephone appointm ent	Primary Care visit (family medicine, internal medicine, etc.)

No	Somewhat disagree	Somewhat agree	Somewhat disagree	Maybe	None		I think telehealth would have a lower quality of care

No	Somewhat agree	Somewhat agree	Strongly agree	Yes	Primary Care visit (family medicine, internal medicine, etc.)	None

No	Somewhat disagree	Somewhat agree	Somewhat disagree	Maybe	Appointment to discuss and adjust medications only		I prefer in-person care,I think telehealth would have a lower quality of care

Yes	1-5	Video appointment from home	Primary Care visit (family medicine, internal medicine, medicine, etc.)		Somewhat disagree	Strongly agree	Somewhat disagree	No	I prefer in-person care,I think telehealth would have a lower quality of care

No	Somewhat agree	Somewhat agree	Somewhat agree	Yes	Behavioral/Mental health therapy,Primary Care visit (family medicine, internal medicine, etc.),Appointment to discuss and adjust medications only	None

	No					Neither agree nor disagree	Somewhat agree	Somewhat agree	Maybe	Appointment to discuss and adjust medications only		I prefer in-person care,I think telehealth would have a lower quality of care
Utilized telehealth roughly the same before and after November 2021	No					Neither agree nor disagree	Somewhat agree	Neither agree nor disagree	Yes	Primary Care visit (family medicine, internal medicine, etc.),Appointment to discuss and adjust medications only		None

No

No	Neither agree nor disagree	Neither agree nor disagree	Somewhat agree	Maybe	Primary Care visit (family medicine, internal medicine, etc.),Appointment to discuss and adjust medications only	None

Utilized telehealth more since November 2021	Yes	1-5	Video appointment from home	Primary Care visit (family medicine, internal medicine, etc.),Specialty care (cardiologist, pulmonologist, nephrologist, etc)		Somewhat agree	Somewhat agree	Strongly agree	Yes	Behavioral /Mental health therapy,Primary Care visit (family medicine, internal medicine, etc.),Specialty care (cardiologist, pulmonologist, nephrologist, etc),Appointment to discuss and adjust medications only		None

No	Somewhat disagree	Strongly agree	Somewhat disagree	No	I prefer in-person care

Utilized telehealth roughly the same before and after November 2021	No					Somewhat agree	Strongly agree	Strongly agree	Yes	Behavioral/Mental health therapy,Physical therapy (guided exercises, post-operation therapy, etc.),Specialty care (cardiologist, pulmonologist, nephrologist, etc),Appointment to discuss and adjust medications only		I think telehealth would have a lower quality of care

No					Somewhat disagree	Somewhat agree	Neither agree nor disagree	Yes	Behavioral/Mental health therapy,Appointment to discuss and adjust medications only,Other (please specify)	Something such as a cold or flu symptoms	I prefer in-person care,I think telehealth would have a lower quality of care

Utilized telehealth more before November 2021	Yes	1-5	Video appointment from home	Other (please specify)	Rheumatology	Somewhat agree	Somewhat agree	Somewhat agree	Yes	Behavioral/Mental health therapy,Appointment to discuss and adjust medications only		I do not have quality internet service

No	Neither agree nor disagree	Strongly agree	Somewhat agree	Yes	Appointment to discuss and adjust medications only	I prefer in-person care,I think telehealth would have a lower quality of care,Other (please specify)

No					Somewhat agree	Strongly agree	Strongly agree	Yes	Surgery follow-up,Primary Care visit (family medicine, internal medicine, etc.),Appointment to discuss and adjust medications only		I prefer in-person care,I think telehealth would have a lower quality of care

Utilized telehealth more since November 2021	Yes	1-5	Video appointment from home	Appointment to discuss and adjust medications only		Somewhat agree	Strongly agree	Somewhat agree	Yes	Behavioral/Mental health therapy,Primary Care visit (family medicine, internal medicine, etc.),Substance abuse treatment program,Appointment to discuss and adjust medications only		None
	No					Neither agree nor disagree	Strongly agree	Somewhat agree	Maybe			

Utilized telehealth more before November 2021	No					Strongly agree	Somewhat agree	Strongly agree	Yes	Primary Care visit (family medicine, internal medicine, etc.)		None	
Utilized telehealth roughly the same before and after November 2021	No					Strongly agree	Strongly agree	Strongly agree	Yes	Surgery follow-up,Specialty care (cardiologist, pulmonologist, nephrologist, etc)		None	

Utilized telehealth roughly the same before and after November 2021	No					Strongly agree	Strongly agree	Strongly agree	Yes	Behavioral/Mental health therapy,Surgery follow-up,Primary Care visit (family medicine, internal medicine, etc.),Specialty care (cardiologist, pulmonologist, nephrologist, etc),Appointment to discuss and adjust medications only		None

Utilized telehealth roughly the same before and after November 2021	Yes	1-5	Video appointment from home	Specialty care (cardiologist, pulmonologist, nephrologist, etc)		Somewhat agree	Somewhat agree	Somewhat agree	Yes	Behavioral/Mental health therapy,Appointment to discuss and adjust medications only		I prefer in-person care,I think telehealth would have a lower quality of care,I have a lack of privacy at home

Utilized telehealth roughly the same before and after November 2021	No					Somewhat agree	Strongly disagree	Somewhat disagree	Maybe	Primary Care visit (family medicine, internal medicine, etc.),Specialty care (cardiologist, pulmonologist, nephrologist, etc),Appointment to discuss and adjust medications only		I do not have quality internet service

No						Strongly agree	Somewhat disagree	Strongly agree	Yes	Physical therapy (guided exercises, post-operation therapy, etc.),Specialty care (cardiologist, pulmonologist, nephrologist, etc),Appointment to discuss and adjust medications only		I do not have quality internet service	

No	Neither agree nor disagree	Strongly agree	Somewhat agree	Maybe	Primary Care visit (family medicine, internal medicine, etc.),Specialty care (cardiologist, pulmonologist, nephrologist, etc),Appointment to discuss and adjust medications only	I prefer in-person care

Yes	1-5	Telephone appointment	Other (please specify)	Telephone wellness check for Humana	Neither agree nor disagree	Strongly agree	Strongly disagree	Yes	Surgery follow-up,Primary Care visit (family medicine, internal medicine, etc.),Specialty care (cardiologist, pulmonologist, nephrologist, etc),Other (please specify)	Wellness check	I prefer in-person care
No					Somewhat disagree	Somewhat disagree	Strongly agree	No			I do not have quality internet service,I prefer in-person care

	No					Strongly disagree	Somewhat agree	Strongly disagree	No			I prefer in-person care
Utilized telehealth roughly the same before and after November 2021	Yes	1-5	Video from physician's office to another provider	Specialty care (cardiologist, pulmonologist, nephrologist, etc)		Somewhat disagree	Somewhat agree	Neither agree nor disagree	Maybe	Primary Care visit (family medicine, internal medicine, etc.),Appointment to discuss and adjust medications only		I prefer in-person care,I think telehealth would have a lower quality of care,I lack knowledge/information on telehealth programs
	Yes	1-5	Telephone appointment	Primary Care visit (family medicine, internal medicine, etc.)		Neither agree nor disagree	Somewhat disagree	Neither agree nor disagree	Maybe	Primary Care visit (family medicine, internal medicine, etc.)		I prefer in-person care

	No					Somewhat agree	Strongly disagree	Somewhat agree	Yes	Specialty care (cardiologist, pulmonologist, nephrologist, etc)		I do not have quality internet service,I lack knowledge/information on telehealth programs
	No					Strongly agree	Strongly agree	Strongly agree	Yes	Primary Care visit (family medicine, internal medicine, etc.)		I do not have quality internet service
Utilized telehealth roughly the same before and after November 2021	Yes	1-5	Telephone appointment	Primary Care visit (family medicine, internal medicine, etc.)		Strongly agree	Strongly agree	Strongly agree	Yes	Surgery follow-up,Primary Care visit (family medicine, internal medicine, etc.)		None

No					Somewhat agree	Strongly agree	Strongly agree	Yes	Surgery follow-up,Primary Care visit (family medicine, internal medicine, etc.),Specialty care (cardiologist, pulmonologist, nephrologist, etc),Appointment to discuss and adjust medications only		I lack knowledge/information on telehealth programs		

Utilized telehealth roughly the same before and after November 2021	No		Somewhat disagree	Neither agree nor disagree	Neither agree nor disagree	Yes	Primary Care visit (family medicine, internal medicine, etc.)	I do not have quality internet service,I think telehealth would have a lower quality of care

No	Neither agree nor disagree	Strongly disagree	Somewhat agree	No	I do not have quality internet service,I do not have internet capable devices (smartphone, iPad, laptop/computer, etc.),I lack knowledge/information on telehealth programs

Q26_8_TEX	Q27	Q27_8_TEX	Q30
What conc	What poter	What poter	OPTIONALWhat is your overall opinion on utilizing telehealth in Martin County? You can list concerns, benefits, com
	Improved access to specialty care,I can avoid people I know seeing me in the waiting room,I can access different providers		I believe telehealth could really have a positive impact on the health of Martin County. I am just not sure if it is feasible in terms of internet access

Improved access to primary care,Improved access to specialty care,I can access different providers

I can avoid
people I
know
seeing me
in the
waiting
room

None	Telehealth can only be used for test results or general checking in appts. If I have problem, the doctor needs to see me in person, as a video or telephone cannot give a full picture of what is going on.

I can avoid/minimize travel,There is reduced exposure to sick individuals

I would rather see more in person options in Martin County, but in the absence of in person options it will do. Internet options need major improvements.

I can avoid/minimize travel,There is reduced exposure to sick individuals	Enjoy having the option to use telehealth for my appointment, however sometimes have issues with our DSL service in our area staying connected

There is reduced exposure to sick individuals

Improved access to primary care,Improved access to specialty care,I can avoid people I know seeing me in the waiting room,I can avoid/minimize travel,There is reduced exposure to sick individuals,I can access different providers	I think it would be great!

I can
avoid/mini
mize
travel,Ther
e is
reduced
exposure
to sick
individual
s,I can
access
different
providers

I think we
have a lot
of citizens
who do
not like
change
and have
routine of
going to
see the
doctor. I
think
many,
especially
the aging
populatio
n, will
have a
hard time
adjusting
to taking
to the
doctor
through a
screen. I,
as a young
individual,
would still
rather go

Improved access to primary care,I can avoid/minimize travel,There is reduced exposure to sick individuals	It is needed

Improved access to primary care,Improved access to specialty care,I can avoid/minimize travel,There is reduced exposure to sick individuals,I can access different providers

I can avoid/minimize travel,There is reduced exposure to sick individuals	I don’t think a lot of people in Martin County have access to strong enough internet to have telehealth appointments.

Improved access to primary care,I can avoid/minimize travel,There is reduced exposure to sick individuals

I feel I would participate. Concerns would be how chest pain, blood pressure problems, things that would need to be assessed. It would be nice though for things such as sinus infections and that type of thing. Something you just don't feel like

Improved access to primary care,Improved access to specialty care,There is reduced exposure to sick individuals,I can access different providers	I think it would be beneficial for specific appointments such as therapy or changing medication. If I would have to choose in-person or telehealth I would choose in-person. Doctors cannot run tests or even check vitals through online and you

Improved access to primary care,Impr oved access to specialty care,I can avoid/mini mize travel,Ther e is reduced exposure to sick individual s	Those of us who are rural with limited cell service and not adequate internet service do not have the ability to utilize tele health

Improved access to primary care,Improved access to specialty care,There is reduced exposure to sick individuals	Ilimitex telehealth and poor internet in rural areas
None	

I can avoid people I know seeing me in the waiting room,There is reduced exposure to sick individuals,I can access different providers There is reduced exposure to sick individuals

I can avoid people I know seeing me in the waiting room,I can avoid/minimize travel,There is reduced exposure to sick individuals	I think telehealth has benefits. But in Martin county I feel that several don’t have the Internet or high speed Internet to do so. If we eventually go all telehealth, I believe in Martin Co. would loose a lot of patients who really need vital care.

Improved access to specialty care,I can avoid people I know seeing me in the waiting room,I can avoid/minimize travel,There is reduced exposure to sick individuals

Improved
access to
primary
care,I can
avoid/mini
mize
travel

There is
reduced
exposure
to sick
individual
s

Improved access to primary care,Improved access to specialty care,I can avoid/minimize travel,There is reduced exposure to sick individuals,I can access different providers

There is reduced exposure to sick individual s	i would use it because i don't want to be around a lot of sick people and i'm sure other people feel the same.

Feel that I need to see a cardiologist in person so the provider can actually listen to my heart.	Improved access to primary care,Improved access to specialty care,I can avoid people I know seeing me in the waiting room,I can avoid/minimize travel,There is reduced exposure to sick individuals		I think telehealth in Martin County would be beneficial.

Improved access to specialty care,I can avoid/minimize travel,There is reduced exposure to sick individuals,I can access different providers

Improved access to specialty care,I can avoid/minimize travel,There is reduced exposure to sick individuals,I can access different providers	There will be a cultural adjustment based on rural setting.

I can avoid people I know seeing me in the waiting room,I can avoid/minimize travel,There is reduced exposure to sick individuals

None

I can
avoid/mini
mize
travel

Improved access to primary care,Improved access to specialty care,I can avoid people I know seeing me in the waiting room,I can avoid/minimize travel,There is reduced exposure to sick individuals,I can access different providers

None
Improved access to primary care,There is reduced exposure to sick individuals

I can access different providers

I can
avoid/mini
mize
travel

Improved access to primary care,Improved access to specialty care,I can avoid/minimize travel,There is reduced exposure to sick individuals	It's a smaller community with an older population that may not prefer, or know how to use the telehealth. I think overall that it is s great option.

I can avoid people I know seeing me in the waiting room,I can avoid/minimize travel,There is reduced exposure to sick individuals,I can access different providers	
None	I am more comfortable speaking to a physician in person.

I can
avoid/mini
mize
travel

Improved
access to
primary
care,Impr
oved
access to
specialty
care,I can
avoid/mini
mize
travel,Ther
e is
reduced
exposure
to sick
individual
s,I can
access
different
providers

Improved access to primary care,Improved access to specialty care,I can avoid people I know seeing me in the waiting room,I can avoid/minimize travel,There is reduced exposure to sick individuals,I can access different providers

Improved access to primary care,Improved access to specialty care,I can avoid/minimize travel	I have not used telehealth as I was fortunate to not require healthcare that I couldn’t do in person. But I was employed in a mental health provider clinic that utilized telehealth. I do think it was beneficial during the pandemic. That being

Improved
access to
primary
care,I can
avoid/mini
mize
travel,I
can
access
different
providers

I can avoid/minimize travel,There is reduced exposure to sick individuals,Other (please specify)	Change of medication	I only had to use it once. I was sick with respiratory symptoms. The one thing I didn't like was the doctor couldn't listen to my lungs but she could hear my congested cough over the phone. I don't think a doctor can do as thorough exam over the phone

There is reduced exposure to sick individual s

Improved access to primary care

I feel on certain things it would be fine. But in some cases I feel like seeing a Dr in person would be better.	Improved access to primary care,I can avoid/minimize travel,There is reduced exposure to sick individuals,I can access different providers

Improved access to primary care,Improved access to specialty care,I can avoid/minimize travel,There is reduced exposure to sick individuals,I can access different providers	I think it would be a great benefit for Martin County, although many individuals in Martin County would not have the appropriate internet connection/services.

I can
avoid/mini
mize
travel,Ther
e is
reduced
exposure
to sick
individual
s
There is
reduced
exposure
to sick
individual
s

I can avoid/minimize travel,There is reduced exposure to sick individuals

None	Teel health and phone visits cannot replace in person visits. Often times the types of visits I have require lab work and specialized equipment.

I can
avoid/mini
mize
travel,Ther
e is
reduced
exposure
to sick
individual
s,I can
access
different
providers

I can
avoid/mini
mize
travel

Improved access to specialty care

Improved access to primary care,Improved access to specialty care,I can avoid/minimize travel,There is reduced exposure to sick individuals	The one time I used telehealth I was pleased with how smoothly it went. The doctor was patient with me and addressed any questions I had. He was on time and I felt that we were able to complete the appointment in a timely manner.

I can avoid people I know seeing me in the waiting room,I can avoid/minimize travel,There is reduced exposure to sick individuals,I can access different providers

None

I can avoid/minimize travel,There is reduced exposure to sick individuals

There is
reduced
exposure
to sick
individual
s

Improved access to primary care,I can avoid/minimize travel,There is reduced exposure to sick individuals,I can access different providers	It would save time and travel.I would prefer in person.

None

Improved access to primary care,I can avoid/minimize travel

Improved access to primary care

I can avoid
people I
know
seeing me
in the
waiting
room

Other (please specify)	less hassle	I don’t believe a telehealth visit would be adequate for dealing with any serious issues as no actual physical exam can be done. It would probably be adequate for follow up visits, reviewing blood work, renewing prescriptions, etc.

Improved access to primary care,Improved access to specialty care,I can avoid people I know seeing me in the waiting room,I can avoid/minimize travel,There is reduced exposure to sick individuals,I can access different providers

I can avoid people I know seeing me in the waiting room

I can avoid/minimize travel,There is reduced exposure to sick individuals

I can avoid people I know seeing me in the waiting room,I can avoid/minimize travel,There is reduced exposure to sick individuals

I can avoid/minimize travel

I can
avoid/mini
mize
travel

I can
avoid/mini
mize
travel

I can
avoid/mini
mize
travel

I can avoid/minimize travel,There is reduced exposure to sick individuals

I can avoid people I know seeing me in the waiting room,I can avoid/minimize travel

Improved access to primary care,I can avoid/minimize travel,There is reduced exposure to sick individuals,I can access different providers

There is reduced exposure to sick individuals

I can avoid people I know seeing me in the waiting room

Improved access to specialty care,I can avoid/minimize travel

Improved access to primary care,I can avoid/minimize travel,There is reduced exposure to sick individuals

Improved access to primary care,Improved access to specialty care,I can avoid people I know seeing me in the waiting room,I can avoid/minimize travel,There is reduced exposure to sick individuals,I can access different providers	I would love it!

Using telehealth when in-person should be used for the physical aspect.	I can avoid/minimize travel,There is reduced exposure to sick individuals		Provides opportunity for better/more health care. Concerned that some patients would receive telehealth when they should’ve met in-person, thinking that if telehealth was offered they (the patient) didn’t have much choice.

I can avoid people I know seeing me in the waiting room

I was having a STROKE in the office of my DOCTOR and she failed to properly diagnose me. Why EVER would I trust someone that sees me on a video monitor?

I can avoid/minimize travel,There is reduced exposure to sick individuals

I can
avoid/mini
mize
travel,I
can
access
different
providers
I can
avoid/mini
mize
travel,Ther
e is
reduced
exposure
to sick
individual
s

Improved
access to
primary
care,Impr
oved
access to
specialty
care,I can
avoid/mini
mize
travel,Ther
e is
reduced
exposure
to sick
individual
s,I can
access
different
providers

I can avoid/minimize travel,There is reduced exposure to sick individuals

None

Improved access to specialty care,I can avoid/minimize travel	[illegible] something people could receive daily telehealth appointments in the form of possible questionable emergency's or concerns regarding specific circumstances I could see a real benefits ! Other than that there's to many variables that could

Improved access to primary care,I can avoid/minimize travel,There is reduced exposure to sick individuals

I can avoid/minimize travel,There is reduced exposure to sick individuals

I think it is beneficial for med changes or weekend appointments.

I can avoid/minimize travel,There is reduced exposure to sick individuals

I can avoid/minimize travel,There is reduced exposure to sick individuals

There is
reduced
exposure
to sick
individual
s

Improved
access to
primary
care

Improved access to primary care

Improved access to primary care,Improved access to specialty care,I can avoid/minimize travel,There is reduced exposure to sick individuals,I can access different providers	I am open to telehealth for convenience, travel expense, and I have good experience with it in the past. Of course there are times when you need tests and face to face appointments.

I can avoid/minimize travel,There is reduced exposure to sick individuals

I can avoid people I know seeing me in the waiting room,I can avoid/minimize travel,There is reduced exposure to sick individuals

Improved access to primary care,Improved access to specialty care,I can avoid/minimize travel,There is reduced exposure to sick individuals,I can access different providers	My daughter has a feeding tube and some other specialty healthcare needs. She is seen At Riley Children’s every 4-6 weeks. Because of this telehealth has benefited us because it has cut our road time down considerably. But I also

I can avoid/minimize travel

Improved access to primary care,I can avoid/minimize travel,There is reduced exposure to sick individuals

I can avoid/minimize travel,There is reduced exposure to sick individuals,I can access different providers

I can avoid people I know seeing me in the waiting room,I can avoid/minimize travel,There is reduced exposure to sick individuals,I can access different providers

None	I prefer my in person provider. I would use it if needed., I wouldn't say I would never use it

Improved access to primary care,I can avoid people I know seeing me in the waiting room,I can avoid/minimize travel,There is reduced exposure to sick individuals	I think it would be a wonderful service for Martin County. Given that we are in a desert for health providers, and we don’t really have a good use for rural transportation, it would be great for those that don’t have access to transportation.

Doctor not being able to be hands on.	I can avoid people I know seeing me in the waiting room,I can avoid/minimize travel,There is reduced exposure to sick individuals

Improved access to primary care,I can avoid/minimize travel,There is reduced exposure to sick individuals,I can access different providers	Fiber optic is not available to everyone in the county and there is no timeframe when it will be. My one telehealth appointment I had was a little difficult to set up. I had the school nurse, at work, take my vitals prior, so I could tell the doctor. I was at

None	I probably wouldn't use it.
I can avoid people I know seeing me in the waiting room,I can avoid/minimize travel,There is reduced exposure to sick individuals	

Improved access to primary care,Improved access to specialty care,I can avoid people I know seeing me in the waiting room,I can avoid/minimize travel,There is reduced exposure to sick individuals

I feel there are certain circumstances where being seen in person is crucial for proper diagnosis/care	There is reduced exposure to sick individuals,Other (please specify)	Quick care for minor issues

There is reduced exposure to sick individuals	In my experience and with my husband's it seems like if you call and have a fever they always want you to go to the ER. This is my only complaint ...you don't need to go to er Everytime for fever that kinda defeats the purpose of teledoc.

None
Improved access to primary care,Improved access to specialty care,I can avoid people I know seeing me in the waiting room,There is reduced exposure to sick individuals

I can avoid people I know seeing me in the waiting room,There is reduced exposure to sick individuals	None
I can avoid/minimize travel,There is reduced exposure to sick individuals	

I can
avoid/mini
mize
travel

I can avoid/minimize travel,There is reduced exposure to sick individuals

I can avoid people I know seeing me in the waiting room,I can avoid/minimize travel,There is reduced exposure to sick individuals

Improved
access to
specialty
care,I can
avoid/mini
mize
travel,I
can
access
different
providers

I can
avoid/mini
mize
travel,I
can
access
different
providers

I can avoid people I know seeing me in the waiting room,I can avoid/minimize travel,There is reduced exposure to sick individuals

I think it would be great for the elderly that need to talk to a medical professional and can't drive. For my own person experience it has just been easier to make appointments and minimize travel times.

None	I think due to our socioeconomic status for our area, access to in person Healthcare is important in our residents being able to properly care for themselves. They need education

Improved access to primary care,Improved access to specialty care,I can access different providers

I can avoid/minimize travel

None

Improved
access to
specialty
care,I can
avoid/mini
mize
travel

Improved access to primary care,Improved access to specialty care,I can avoid/minimize travel,There is reduced exposure to sick individuals

Improved access to primary care,I can avoid/minimize travel,There is reduced exposure to sick individuals,I can access different providers	Most don't have dependable internet, I only do because I pay out the nose for it.

Improved access to primary care,Improved access to specialty care,I can avoid people I know seeing me in the waiting room,I can avoid/minimize travel,There is reduced exposure to sick individuals,I can access different providers

Improved access to specialty care,I can avoid/minimize travel,There is reduced exposure to sick individuals,I can access different providers

There is reduced exposure to sick individuals

I can avoid/minimize travel,There is reduced exposure to sick individuals	The main benefit is not having to take your child that is sick with a fever/virus/sinus infection outside the home to drive, wait, see the doctor, drive back, and wait for pharmacy to fill medicine. 2-3 hours just right there when all your child wants to do is rest.

There is reduced exposure to sick individuals

I can avoid/minimize travel,There is reduced exposure to sick individuals

I can avoid/minimize travel,There is reduced exposure to sick individuals

I can avoid/minimize travel,There is reduced exposure to sick individuals

Improved access to primary care,I can avoid/minimize travel,There is reduced exposure to sick individuals,I can access different providers

I can avoid people I know seeing me in the waiting room,I can avoid/minimize travel

I can avoid people I know seeing me in the waiting room,I can avoid/minimize travel,I can access different providers	
None	Elderly and want to see the doctor in person.

Improved access to primary care,Improved access to specialty care,I can avoid/minimize travel,There is reduced exposure to sick individuals

	There is reduced exposure to sick individuals
Not being about to examine you the way they could in a doctors office or hospital	I can avoid/minimize travel

Improved access to primary care,Improved access to specialty care,I can avoid people I know seeing me in the waiting room,I can avoid/minimize travel,There is reduced exposure to sick individuals,I can access different providers	Myself and several individuals from Martin Co. have voiced enjoying telehealth because we can access medical care from the comfort of our homes without interrupting our work schedule or lives.

Improved access to primary care,Improved access to specialty care,I can avoid people I know seeing me in the waiting room,I can avoid/minimize travel,There is reduced exposure to sick individuals

Improved access to primary care,I can avoid/minimize travel,There is reduced exposure to sick individuals	A lot of Martin county has poor or no internet service.

I can avoid people I know seeing me in the waiting room,I can avoid/minimize travel,There is reduced exposure to sick individuals

None

I can avoid/minimize travel

Again I think for simple visits it would be great. I do worry about the elderly knowing how to use new technology and I worry about many not have an internet in these areas or fast enough service.

Just depends on what the appointment is for. If it's something simple I would not mind, but if it's more complicated I would prefer an in person provider visit.

Improved access to primary care,There is reduced exposure to sick individuals

Improved access to primary care,Improved access to specialty care,I can avoid/minimize travel,There is reduced exposure to sick individuals

Improved access to primary care,Improved access to specialty care,I can avoid/minimize travel,There is reduced exposure to sick individuals,I can access different providers	I prefer not to use it. I would rather see my provider in person.

Improved access to primary care,Improved access to specialty care,I can avoid/minimize travel,There is reduced exposure to sick individuals,I can access different providers	I think it would be great especially when it's cold and flu season and you just need a antibiotic to get over a sinus infection.

There is reduced exposure to sick individuals

It would be beneficial for some populations. Cost may be less. Might be able to get health services sooner than waiting for an in person appointment.

There is reduced exposure to sick individuals

Improved access to primary care,There is reduced exposure to sick individuals	Might be beneficial as heath care here is minimal and hard to get into at times.

Improved access to specialty care,I can avoid/minimize travel,There is reduced exposure to sick individuals,I can access different providers

It would be very convenient for me as I travel to Jasper for cardiology and digestive issues, Bloomington for dermatology, and Washington for diabetes and thyroid problems. At my age, it is sometimes difficult to travel.

Improved access to primary care,Improved access to specialty care,I can avoid/minimize travel,There is reduced exposure to sick individuals	I do like telehealth appointments and wish that there wasn't a limitation in the type of visits or medicines that can be prescribed. For example, muscle pain- muscle relaxer. Maybe it's the first option and you can get a short prescription until you can

Improved access to specialty care,I can avoid/minimize travel	I recently made the trip from Martin County to Carmel, Indiana to see a specialist. While I really like the doctor, I hate the long drive to the city and back. The trip made for a very long day and added a great deal of stress to an already stressful situation. Any follow-

None	I prefer face to face appointments, I feel the health care provider can better assess my issues and conditions.
There is reduced exposure to sick individuals	I haven’t used it yet so I can’t answer if it is a benefit or not

Improved access to primary care,Improved access to specialty care,There is reduced exposure to sick individuals	It would probably be very beneficial, since it’s so hard to get into the doctor at a reasonable time. Especially if you are sick and need medication quickly.

Improved access to primary care,Improved access to specialty care,I can avoid/minimize travel,There is reduced exposure to sick individuals,I can access different providers	Bring us to into the current century.

There is reduced exposure to sick individuals	I prefer to have one on one time with my healthcare provider. I feel I receive better care. At the beginning of the pandemic as a nurse I used telehealth for some of my residents at a nursing home. I just felt it was rushed, and their needs

I can avoid/minimize travel,I can access different providers

I rather have personal face to face interaction.

I can
avoid/mini
mize
travel

I can avoid/minimize travel,There is reduced exposure to sick individuals

There is reduced exposure to sick individuals,I can access different providers

Improved
access to
specialty
care,I can
avoid/mini
mize
travel,I
can
access
different
providers

Improved access to primary care,I can avoid/minimize travel,There is reduced exposure to sick individuals

I can access different providers

Appointments don’t feel as personal, especially for mental health.	I can avoid/minimize travel,There is reduced exposure to sick individuals

I can avoid/minimize travel,There is reduced exposure to sick individuals

I can
access
different
providers

Improved access to specialty care	Not good enough internet in this rural area even though RTC has fiber optic it’s 2 miles from me and they will not cross Boggs Creek to give the rest of the residence pass that creek fiber optic our Internet is really bad. We are signed up for Starlink that’s

Improved access to primary care,Improved access to specialty care,I can avoid people I know seeing me in the waiting room,I can avoid/minimize travel,There is reduced exposure to sick individuals,I can access different providers

None

I can avoid/minimize travel,There is reduced exposure to sick individuals

There is reduced exposure to sick individual s	Telehealth seems not personal care. I feel I lose a lot of care .

Improved access to primary care,Improved access to specialty care,I can avoid people I know seeing me in the waiting room,I can avoid/minimize travel,There is reduced exposure to sick individuals,I can access different providers

Improved access to primary care,I can avoid/minimize travel,There is reduced exposure to sick individuals	internet in martin county sucks!

Improved access to primary care,I can avoid people I know seeing me in the waiting room,I can avoid/minimize travel,There is reduced exposure to sick individuals,I can access different providers	I think it's great but should also be a patient's right to choose how they like to visit their doctor. I'd worry about elderly trying to do telehealth.

I believe physical examinations, having blood pressure and heart rate checks, and monitoring weight are all important part of physical exams. I believe several illness would only be able to be diagnosed by the doctor doing a complete	Improved access to primary care,I can avoid/minimize travel,There is reduced exposure to sick individuals	While I believe there are some cases Telehealth is a good idea (especially for mental/behavioral therapy), there are many times i believe Telehealth would be inappropriate. I believe physical examinations, having blood pressure and heart

Improved access to primary care,Improved access to specialty care,I can avoid people I know seeing me in the waiting room,I can avoid/minimize travel,There is reduced exposure to sick individuals,I can access different providers	I have no concerns using telehealth or video appointments. I am a healthcare worker with 21 years experience with family practice and I think 90% of all the appointments we see could be handled via phone or video. I think as far as mental

None	
I can avoid/minimize travel	
Improved access to specialty care	Internet service is poor in Martin county. While my internet works well it can often be out for days at a time.

There is reduced exposure to sick individual s

Improved access to primary care

Improved access to primary care,Improved access to specialty care,I can avoid/minimize travel,There is reduced exposure to sick individuals	I’ve only lived in Martin County a few months, having moved back from Greene County.

Improved
access to
primary
care

None

Improved access to primary care,There is reduced exposure to sick individuals	Overall internet access in rural areas is dismal in Martin county. While Loogootee has two high speed providers, rtc and sparklight, if you live on 550 after bridge shoals side, it’s frontier. The speed does not even register on a speed test.

There is reduced exposure to sick individuals	My concern is the quality and thoroughness of a telehealth appointment. The physician would not be able to run tests or make a full examination from the computer. I prefer in-person appointments.

I can avoid people I know seeing me in the waiting room,I can avoid/minimize travel,There is reduced exposure to sick individuals

Improved access to primary care,Improved access to specialty care,There is reduced exposure to sick individuals,I can access different providers

There is reduced exposure to sick individuals

I can avoid/minimize travel,There is reduced exposure to sick individuals

I can
access
different
providers

Improved access to primary care,Improved access to specialty care,I can avoid people I know seeing me in the waiting room,I can avoid/minimize travel,There is reduced exposure to sick individuals,I can access different providers

I can avoid/minimize travel	I would rather see my provider in person and ensure that I'm getting care face to face. I don't know what else is going on in the provider's office that may be distracting and keep them from providing me excellent quality care.

Improved access to primary care,Improved access to specialty care,I can avoid/minimize travel,There is reduced exposure to sick individuals	I feel that using telehealth in most circumstances is a good idea due to transportation barriers. However, I do believe that telehealth should never and will never take the place of in person visits with your health care provider.

I can avoid
people I
know
seeing me
in the
waiting
room,I
can
avoid/mini
mize
travel

I can avoid/minimize travel,There is reduced exposure to sick individuals	I have to use my iPhone for video telehealth appts but at times I don’t have quality or lose my provider halfway through. I prefer my appts with my Indy rheumatologist via telehealth due to long travel times & not feeling well enough to travel at times.

I believe that if the appointment was in regards to mental health, an in person visit would have a greater impact on feeling a connection or safe.

I can avoid/minimize travel,There is reduced exposure to sick individuals,I can access different providers

I think telehealth would help with more access to medical providers. Martin county only has a few doctors and nurses. This could potentially cut back on wait times and the community could potentially have issues treated that have been put

Improved access to primary care,Improved access to specialty care,I can avoid people I know seeing me in the waiting room,I can avoid/minimize travel,There is reduced exposure to sick individuals,I can access different providers

Improved access to primary care,Improved access to specialty care,I can avoid/minimize travel,There is reduced exposure to sick individuals,I can access different providers

Improved access to primary care,Improved access to specialty care,I can avoid/minimize travel,There is reduced exposure to sick individuals,I can access different providers

I can avoid people I know seeing me in the waiting room

Improved access to specialty care,I can avoid people I know seeing me in the waiting room,I can avoid/minimize travel,There is reduced exposure to sick individuals,I can access different providers

	I do it out of necessity. Would prefer in-person care. I want my Dr. to be able to actually see me & assess me.
Improved access to specialty care,I can avoid/minimize travel,There is reduced exposure to sick individuals,I can access different providers	It is hard as a stay-at-home-mom to really be free to talk while at home. Trying to occupy busy kiddos while talking to a dr. in my

Improved access to primary care,Improved access to specialty care

Improved access to primary care,Improved access to specialty care,There is reduced exposure to sick individuals	Great service this would be!

I can
access
different
providers

Improved access to primary care,Improved access to specialty care,I can avoid people I know seeing me in the waiting room,There is reduced exposure to sick individuals	Many items can be diagnosed and dealt with over the phone on computer, especially minor flu & cold items
None	

I can access different providers	Better internet More providers - Doctor, not NP
I can avoid/minimize travel,There is reduced exposure to sick individuals,I can access different providers	We need better internet access
I can avoid/minimize travel,There is reduced exposure to sick individuals	Not a bad idea

I can access different providers	
Improved access to primary care	Love it! Better internet service for use
Improved access to primary care,Improved access to specialty care,I can avoid/minimize travel	Love it!

I can avoid/minimize travel,There is reduced exposure to sick individuals,I can access different providers

None	It's not personal with the family medicine doctor

I can avoid/minimize travel,I can access different providers

There is a lack of reliable and affordable internet. Some places still do not have coverage!

plaints, or personal experiences.
